# The role of aging on endothelial cell–cell junctions and pulmonary microvascular permeability in male mice

**DOI:** 10.14814/phy2.70686

**Published:** 2025-12-19

**Authors:** Aminmohamed Manji, Lefeng Wang, Cynthia Pape, Sanjay Mehta, Preya Patel, Samuel‐Caleb Yeung, Eric K. Patterson, Antoine Dufour, Daniel Young, Ruud A. W. Veldhuizen, Sean E. Gill

**Affiliations:** ^1^ Centre for Critical Illness Research, London Health Sciences Centre Research Institute London Ontario Canada; ^2^ Department of Physiology and Pharmacology Schulich School of Medicine and Dentistry, Western University London Ontario Canada; ^3^ Department of Medicine Schulich School of Medicine and Dentistry, Western University London Ontario Canada; ^4^ Department of Physiology and Pharmacology Cumming School of Medicine, University of Calgary Calgary Alberta Canada

**Keywords:** aging, cell–cell junctions, pulmonary microvascular endothelial cells, vascular permeability

## Abstract

Pulmonary microvascular endothelial cell (PMVEC) intercellular junctions are critical for maintaining barrier function and mitigating pulmonary edema. Previously, we demonstrated that aging exacerbated pulmonary microvascular permeability in a model of lung injury. Based on this, we hypothesized that aging was associated with increased PMVEC barrier dysfunction due to impaired cell–cell junction integrity. PMVEC were isolated from young and aged mice and cultured to confluence in vitro. Barrier function, junctional integrity, alterations in the proteome, markers of inflammation, and actin cytoskeleton organization were all assessed. To model injurious conditions, PMVEC were stimulated with inflammatory cytokines. PMVEC from aged mice exhibited increased permeability, both under basal and inflammatory conditions, which was associated with disrupted cell‐surface localization of the adherens junction protein, vascular endothelial (VE)‐cadherin. Protein abundance of VE‐cadherin was increased with age, while levels of the adapter protein, γ‐catenin, and the tight junction protein, claudin‐5, were decreased. Measures of inflammation, including cytokine expression and cell surface abundance of adhesion molecules, did not differ with age. Augmented presence of actin stress fibers was observed in aged PMVEC. We conclude that aging predisposes PMVEC to elevated injury, due to inherent deficiencies in cell–cell junctions and barrier function, potentially mediated through altered actin cytoskeleton organization.

## Graphical abstract

Aging contributes to impairments in pulmonary microvascular endothelial cell‐cell junctions and barrier function, leading to an increased predisposition to injury. Created in BioRender. Gill, S. (2025) https://BioRender.com/l14r5v8

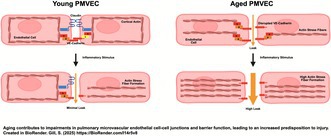

## 1 INTRODUCTION

Under healthy conditions, the pulmonary microvasculature serves as a selectively permeable barrier that prevents the leak of fluids and large macromolecules from circulation into the lung interstitium (Komarova et al., 2017). However, during lung injury, such as with mechanical ventilation‐induced lung injury or acute respiratory distress syndrome (ARDS), widespread inflammation occurs within the lungs, leading to activation and injury of the pulmonary microvascular endothelial cells (PMVEC) (Abadie, 2005; Dudek & Garcia, 2001; Lai & Huang, 2021; London et al., 2010). This results in microvascular barrier dysfunction and the accumulation of protein‐rich edema fluid within the alveolar spaces, leading to impaired gas exchange and respiratory failure (Matthay et al., 2019).

A critical component of the normal pulmonary microvascular barrier is the presence of inter‐PMVEC cell–cell junctions (Wettschureck et al., 2019). This includes adherens junctions, which provide mechanical anchorage between adjacent cells, and tight junctions, which seal paracellular gaps (Komarova et al., 2017; Radeva & Waschke, 2018). Both junctions are necessary to limit permeability and maintain PMVEC barrier function (Wettschureck et al., 2019). The junctions are composed of transmembrane proteins, including vascular endothelial (VE)‐cadherin for the adherens junctions, and claudin‐5 for the tight junctions (Radeva & Waschke, 2018). Additionally, the transmembrane proteins are linked with the underlying actin cytoskeleton via several adapter proteins, such as α‐catenin, β‐catenin, and γ‐catenin for the adherens junctions (Radeva & Waschke, 2018). Binding of the junctional proteins to the cytoskeleton, particularly to thick cortical actin bundles, is crucial for maintaining junctional integrity (Dejana & Vestweber, 2013; Hong et al., 2013; Liu et al., 2010). Damage to the microvasculature, due to the inflammatory processes taking place during lung injury, results in disruption of endothelial cell–cell junctions, formation of actin stress fibers that lead to cell retraction, and cell apoptosis (Abadie, 2005; Dudek & Garcia, 2001; Fujita et al., 1998; London et al., 2010). This subsequently leads to endothelial barrier dysfunction, gap formation within the microvascular walls, and the characteristic augmentation in microvascular macromolecular permeability that is a hallmark of ARDS.

While pulmonary microvascular hyperpermeability is a common feature during lung injury and occurs in all ARDS patients, the elderly population is disproportionately impacted. In fact, the incidence of ARDS rises almost exponentially for every decade of age, and both severity and mortality are known to be exacerbated in elderly ARDS patients (Behrendt, 2000; Ely et al., 2002; Eworuke et al., 2018; Rubenfeld et al., 2005; Schouten et al., 2019; Song et al., 2020; Suchyta et al., 1997). Enhancing our understanding of the role that aging plays in contributing to the increased morbidity and mortality during lung injury will be paramount in improving outcomes in older patients.

Our group has previously demonstrated an age‐associated predisposition to increased pulmonary microvascular permeability in a model of direct lung injury, induced by mechanical ventilation, which was associated with altered endothelial cell–cell junction transcriptional signaling (Manji et al., 2025). Based on this, our current study aimed to further assess the role of aging on pulmonary microvascular barrier function in an in vitro model to directly examine mechanisms at the level of PMVEC. While other studies have examined endothelial barrier function with age and mechanisms associated with cell–cell junction disruption in vitro, they have relied on the induction of senescence as their model of aging (Cheung et al., 2012, 2013; Krouwer et al., 2012; Najari Beidokhti et al., 2025; Stamatovic et al., 2019; Ting et al., 2023; Venkatesh et al., 2011; Ya et al., 2023). Examples of induction of senescence have included repeated cell passaging (Cheung et al., 2012, 2013; Krouwer et al., 2012; Stamatovic et al., 2019; Ya et al., 2023), activation of certain signaling pathways (Venkatesh et al., 2011), and cell‐mediated injury (Najari Beidokhti et al., 2025; Ting et al., 2023), and while useful, these models may not accurately capture the biomolecular processes taking place with natural aging. Notably, the current study was performed in microvascular endothelial cells that were isolated directly from the lungs of young and aged mice, with subsequent analyses performed to assess barrier function. We hypothesized that aging would be associated with increased PMVEC barrier dysfunction due to impaired cell–cell junction integrity.

## 2 METHODS

### 2.1 Model of aging and pulmonary microvascular endothelial cell (PMVEC) isolation

All animal procedures were performed in accordance with the Canadian Council on Animal Care guidelines and approved by the Western University Animal Care Committee (Approval #2020–054). C57BL/6 male mice from Charles River Laboratories were aged to 22 months under controlled conditions (standard light–dark cycles, temperature, humidity, and group housing) within our institutional animal facility. Obesity has been shown to be associated with augmented proinflammatory responses, changes in respiratory mechanics, elevated risk of ARDS, and vascular damage (Anderson & Shashaty, 2021; Bondareva et al., 2022; Gong et al., 2010; Wu et al., 2021). Based on this, the mice were fed (Teklad Irradiated 2019 Envigo chow diet 2919) a food‐restricted diet with a gradual reduction to 70% ad libitum to maintain body weight, as described previously (Tyml et al., 2017). For these studies, PMVEC were isolated from the lungs of young (3‐month‐old) and aged (18–22‐month‐old) mice as previously described (Razavi et al., 2004; Wang et al., 2017). For more detail, please see “Appendix S1” document for this manuscript. Cells from passages 4–10 were used, with growth media being changed every 2 days and at least 16 h prior to experiments. All experiments were performed with independent PMVEC isolations from 2 to 3 young and aged mice, with cells given 72 h to grow to confluence. Cells were isolated exclusively from male mice for practical reasons related to the maintenance of our aging mouse colony during the corona virus disease 2019 (COVID‐19) pandemic. The limitation of using only male mice is addressed in the limitations of the study.

### 2.2 In vitro stimulation of PMVEC

These studies aimed to assess the impact of aging on PMVEC barrier function under basal conditions as well as inflammatory conditions that model ARDS in vitro. An equimolar mixture of murine pro‐inflammatory cytokines (cytomix), including tumor necrosis factor (TNF)α (Peprotech 315‐01A‐20 μg), interleukin (IL)1 β (Peprotech 211‐11B‐10 μg), and interferon (IFN)𝛾 (Peprotech 315‐05‐100 μg), diluted in phosphate‐buffered saline (PBS), was used to model the inflammatory conditions. These cytokines are relevant to inflammatory diseases such as sepsis or COVID‐19, which are known to induce ARDS (Karki et al., 2021; Takahama et al., 2024). Cells were treated by adding cytomix to the growth media at a final concentration of 30 ng/mL for each cytokine, as has been established previously in our lab using mouse PMVEC (Wang et al., 2017). Following 72 h of growth to confluence, stimulation occurred for 4 h to represent an acute time‐point that our lab has previously demonstrated to cause increases in PMVEC permeability, as well as a more long‐term time‐point of 24 h, which has been shown to induce PMVEC cell death (Wang et al., 2017). For control conditions, an equal volume of PBS was added to the cell culture media.

### 2.3 Real‐time assessment of PMVEC monolayer barrier integrity

PMVEC were seeded in duplicates at 5 × 10^4^ cells per well in eight‐well arrays (Applied Biophysics, 8W10E+ PET 72040) coated with 1% gelatin. Cells were grown in complete media, and resistance was continuously monitored at 4000 Hz with an electric cell‐substrate impedance sensing (ECIS) instrument over 72 h (Model Z θ, Applied Biophysics). Resistance values from the duplicates were then averaged, and the data were plotted.

### 2.4 PMVEC monolayer paracellular permeability visualization

To directly assess the relationship between PMVEC monolayer permeability and junctional disruption, a fluorescence technique was employed involving visualization of localized leak based on a modification of the published XPerT permeability assay (Dubrovskyi et al., 2013). For these experiments, PMVEC were seeded at a density of 2.5–3 × 10^4^ cells in 48‐well plates (Corning Falcon 353078) that were coated with biotin (Thermo Scientific 21343)‐conjugated gelatin (Sigma‐Aldrich G2500) overnight at 4°C, just as described in the published XPerT study (Dubrovskyi et al., 2013). Oregon green 488‐conjugated NeutrAvidin (Invitrogen A6374) was added to the cell culture media at a final concentration of 25 μg/mL for 3 min. The cells were then fixed with 200 μL of ice‐cold methanol for 15 min, followed by three washes with PBS to remove residual methanol and remaining unbound avidin. Unlike the initial study describing the XPerT assay (Dubrovskyi et al., 2013), the three PBS washes were performed following fixation rather than after the addition of NeutrAvidin to prevent disturbances to the PMVEC and induction of artificial junctional disruption. Our group has previously shown in human PMVEC that almost all paracellular leak was associated with VE‐cadherin disruption (Wang et al., 2019). Furthermore, disruption of the adherens junction induced by compromised VE‐cadherin adhesion has been shown to be the leading cause of tissue edema in many pathological conditions (Corada, 2001; Crosby et al., 2005; Komarova et al., 2017; Vittet et al., 1997). Based on this, examination of NeutrAvidin leak was performed concurrently with VE‐cadherin staining. Immunocytochemistry for VE‐cadherin staining was carried out as described below. Quantification of NeutrAvidin leak was performed by measuring fluorescence in ImageJ; values were normalized to average baseline levels in the PMVEC isolated from young mice.

### 2.5 Immunocytochemistry of junctional proteins

Cells were fixed as described above, permeabilized with 0.1% Triton X‐100 (VWR), and blocked using 3% bovine serum albumin (BSA) in PBS. The cells were then incubated with antibodies against VE‐cadherin (rabbit polyclonal, 1:200 dilution, Abcam 33168, RRID: AB_870662) or claudin‐5 (mouse monoclonal, 1:100 dilution, Invitrogen 35‐2500, RRID: AB_2533200) in 1% BSA in PBS, followed by incubation with Alexa Fluor 594‐conjugated IgG secondary antibody (donkey anti‐rabbit polyclonal, 1:500 dilution, Invitrogen A11037, RRID: AB_2534095) or Alexa Fluor 488‐conjugated IgG1 secondary antibody (goat anti‐mouse polyclonal, 1:500 dilution, Invitrogen A21121, RRID:AB_2535764). Nuclei were visualized by counterstaining with Hoechst 33342 in PBS (1:5000 dilution, Invitrogen H3570, RRID: AB_3675235). Cells were then imaged at 200× magnification (20× objective) by fluorescent microscopy (Zeiss Axiovert 200 M Inverted Microscope; Carl Zeiss Canada, Toronto, Canada) using the Zeiss Axiovision software for acquisition. Exposure times and contrast levels were kept consistent between the young and aged PMVEC. Four to five images were taken at random locations within each experimental well, excluding the very edges of the well. Image amalgamation and analysis were performed using ImageJ.

### 2.6 PMVEC protein isolation and western blot analysis

PMVEC from young and aged mice were cultured at a density of 2.5 × 10^5^ cells on 6‐well plates (Corning Falcon 353046) coated with 1% gelatin. On the experimental day, media from the wells was aspirated and the cells were washed with ice‐cold PBS. A 200 μL mixture of ice‐cold Cell Lytic M lysis buffer (Sigma‐Aldrich C2978), protease inhibitor cocktail (Sigma‐Aldrich P8340), and 2 mM sodium orthovanadate (phosphatase inhibitor) was added to each well for 20 minutes on ice, with rocking every 5 min. Cell lysates were then sonicated for 10 s (power output of 15% on the Cole Parmer 4710 Series Ultrasonic Homogenizer). Samples were centrifuged at 20,000 × *g* for 15 min at 4°C, the supernatant transferred to a new chilled tube, and protein concentrations assessed using a detergent‐compatible protein assay (Bio‐Rad 5000119) and BSA standards in Cell Lytic M. Protein concentrations were normalized across the samples and gel loading buffer (60 mM Tris pH 6.8, 25% glycerol, 2% sodium dodecyl sulfate [SDS], 14.4 μM β‐mercaptoethanol, 0.1% BromoPhenol Blue, 0.1% H_2_O) was added. Samples were stored at −20°C until ready to use for Western blotting.

For Western blot analysis, samples were thawed on ice, heated to 95°C for 5 min, then loaded onto a 10% or 12% polyacrylamide gel for electrophoresis for approximately 1–2 h (depending on protein of interest). Proteins were then transferred for 45 min to 1 h (depending on protein of interest) to a polyvinylidene fluoride membrane (Bio‐Rad 162‐0177). Membranes were blocked using 3% BSA or 5% skim milk, both diluted in tris‐buffered saline with 0.05% tween‐20 (TBS‐T) for 1 h. Following blocking, the membranes were incubated overnight at 4°C with primary antibodies targeting VE‐cadherin (IgG rabbit polyclonal, 1:2000, Abcam 33168, RRID: AB_870662), α‐catenin (IgG1 mouse monoclonal, 1:1000, BD Transduction Laboratories 610194, RRID: AB_397593), β‐catenin (IgG1 mouse monoclonal, 1:4000, BD Transduction Laboratories Biosciences 610154, RRID: AB_397555), γ‐catenin (IgG2a mouse monoclonal, 1:6000, BD Transduction Laboratories Biosciences 610,254, RRID: AB_397649), claudin‐5 (IgG1 mouse monoclonal, 1:1000, Invitrogen 35‐2500, RRID: AB_2533200), and glyceraldehyde‐3‐phosphate dehydrogenase (GAPDH) (IgG1 mouse monoclonal, 1:5000, Thermo Fisher Scientific MA5‐15738, RRID: AB_10977387), which was used as a loading control. All antibodies were diluted in 1% BSA in TBS‐T, with the exception of claudin‐5, which was diluted in 5% skim milk in TBS‐T. Following antibody incubation, membranes were washed with TBS‐T, then incubated with their appropriate secondary antibodies, including goat anti‐mouse polyclonal IgG1 horseradish peroxidase (HRP) (1:10,000, Abcam Ab98693, RRID: AB_10674928), goat anti‐mouse polyclonal IgG2a HRP (1:10,000, Abcam Ab98698, RRID: AB_10672783), goat anti‐rabbit polyclonal IgG (H + L) HRP (1:10,000, Invitrogen 65‐6120, RRID: AB_2533967), and goat anti‐mouse recombinant polyclonal IgG (H + L) HRP (1:5000 for claudin‐5; 1:10,000 for GAPDH, Invitrogen A28177, RRID: AB_2536163). All secondary antibodies were diluted in 1% BSA in TBS‐T. Membranes were subsequently washed with TBS‐T and then visualized using enhanced chemiluminescence reagent (ECL) (Bio‐Rad Clarity Western ECL Substrate 170‐5060 or Bio‐Rad Clarity Max Western ECL Substrate 1705062) on a MicroChemi camera system (FroggaBio). Densitometry was then performed using ImageJ to calculate protein abundance relative to GAPDH.

### 2.7 PMVEC protein isolation for proteomics analysis

PMVEC from young and aged mice were cultured at a density of 2.5 × 10^5^ cells and grown to confluence on 6‐well plates coated with 1% gelatin. Media from the wells were aspirated, and the cells washed three times with sterile PBS. Cells were lysed with 200 L of ice‐cold sterile lysis buffer (2% SDS, 100 mM Ammonium bicarbonate, 1 mM EDTA, and protease inhibitor cocktail [Sigma‐Aldrich P8340]). Cell lysates were then immediately scraped and collected into ice‐cold Eppendorf tubes. Samples were sonicated for 10 s, put back on ice, then sonicated for another 10 s. Centrifugation was then performed at 14,000 × *g* for 10 min at 4°C and the supernatant transferred to a new chilled tube, which was stored at −80°C. Samples were sent to a core facility at the University of Calgary (Canada) for processing, as has been described previously (Das et al., 2021; Kapilan et al., 2025; Krawetz et al., 2023; Kulle et al., 2024). A detailed description of the proteomics workflow is included in “Appendix S1” document.

### 2.8 PMVEC gene expression analysis

PMVEC from young and aged mice were grown at a density of 2.5 × 10^5^ cells on 6‐well plates coated with 1% gelatin. Media from the wells were aspirated, and cells lysed for RNA collection using the RNeasy Mini Kit (Qiagen 74104), as per the manufacturer's instructions. An on‐column DNA digestion was performed during RNA purification using the RNAse‐Free DNase set, according to the manufacturer's protocol (Qiagen 79254). The RNA was reverse transcribed to cDNA using a high‐capacity cDNA reverse transcription kit (Applied Biosystems 4368814), as per the manufacturer's instructions. Quantitative polymerase chain reaction (qPCR) was then performed on the samples using TaqMan Master Mix (ThermoFisher Scientific 4369016) and probes (all from ThermoFisher Scientific) for interleukin (*Il*)*6* (Mm00446190_m1), CXC motif chemokine ligand (*Cxcl*)*1* (Mm04207460_m1), *Il1* β (Mm00434228_m1), C‐C motif chemokine ligand (*Ccl*)*2* (Mm00441242_m1), *Cxcl2* (Mm00436450_m1), colony stimulating factor (*Csf*)*2* (Mm01290062_m1), intercellular adhesion molecule‐1 (*Icam1*) (Mm00516023_m1), vascular cell adhesion molecule‐1 (*Vcam1*) (Mm01320970_m1), E‐selectin (*Sele*) (Mm01310197_m1), and hypoxanthine‐guanine phosphoribosyltransferase (*Hprt*)*1* (Mm03024075_m1). Subsequent analysis was then performed using the ΔΔCt method, with *Hprt1* used as the housekeeping gene and specific samples normalized to average young PMVEC values. All qPCR experiments were performed with technical duplicates or triplicates, with the average data of the replicates shown.

### 2.9 Assessment of cell adhesion molecule cell surface abundance

The cell surface abundance of ICAM1, VCAM1, and E‐selectin in PMVEC from young and aged mice was assessed using the Guava easyCyte HT Flow Cytometer (EMD Millipore, Billerica, MA). PMVEC were seeded at a density of 2.5 × 10^5^ cells and grown to confluence on 6‐well cell culture plates coated with 1% gelatin. Growth media was aspirated from each well and the cells were gently detached using Accutase (Gibco A1110501) for 30 min at 37°C. The cells were then centrifuged at 300 × *g* for 5 min at room temperature and resuspended in 3 mL of 0.1% BSA/PBS to a concentration of 500,000 cells/mL. Next, 500 μL of the cell suspension was added into three Eppendorf tubes, along with 1 μL of fluorescently labeled antibodies targeted against ICAM1 (rat monoclonal, 0.5 mg/mL, BioLegend 116112, RRID: AB_493493), VCAM1 (rat monoclonal, 0.5 mg/mL, BioLegend 105712, RRID: AB_493429), and E‐selectin (rat monoclonal, 0.2 mg/mL, BD Biosciences 553751, RRID: AB_395031). An additional 500 μL cell aliquot was also taken for an unstained control where no antibody was added. Each tube was incubated with rotation for 1 h at room temperature, followed by three washes with 0.1% BSA/PBS. Cells were then resuspended in 250 μL of 0.1% BSA/PBS and transferred to a clear‐bottom 96‐well round bottom plate to run in the Guava easyCyte HT Flow Cytometer. Live cell gates were set based on forward and side scatter, with the first 10,000 events collected and analyzed per sample. Mean fluorescent intensity was calculated for each sample.

### 2.10 Assessment of PMVEC actin cytoskeleton arrangement

Following growth in 48‐well plates coated with 1% gelatin, PMVEC were fixed with 200 μL of 4% paraformaldehyde for 20 min, washed three times with PBS, permeabilized with 0.1% Triton X‐100 (VWR), and blocked using 3% BSA in PBS. Cells were then incubated in the dark with 200 μL of Phalloidin Tetramethylrhodamine B isothiocyanate (Sigma‐Aldrich P1951; Excitation: 540‐545 nm; Emission: 570–573 nm, RRID: AB_2315148) at a final concentration of 2 μg/mL in PBS for 1 h at room temperature. Counterstaining and fluorescent microscopy imaging were carried out as described above (see “Immunocytochemistry of junctional proteins”). Quantification of actin stress fibers in the PMVEC under basal conditions was performed using ImageJ, based on similar methodology used in the literature (Schulze et al., 2014; Sherrard et al., 2021) (a full description of the methodology is outlined in “Appendix S1”).

### 2.11 Statistical analysis

Unless otherwise specified, data are presented as mean ± standard error of the mean. Statistical analyses were carried out using the Student unpaired *t*‐test and two‐way ANOVA with Tukey's test. Results were considered significant at *p* < 0.05. For the proteomics analysis, the MaxQuant software was used with a peptide FDR of 0.01. Only proteins with a Q‐value <0.05 were used for Metascape analysis. Analyses were performed with a fold change cutoff of 2, as well as a less stringent cutoff of 1.1.

## 3 RESULTS

### 3.1 Assessment of the role of aging on PMVEC barrier function under basal conditions

**FIGURE 1:**
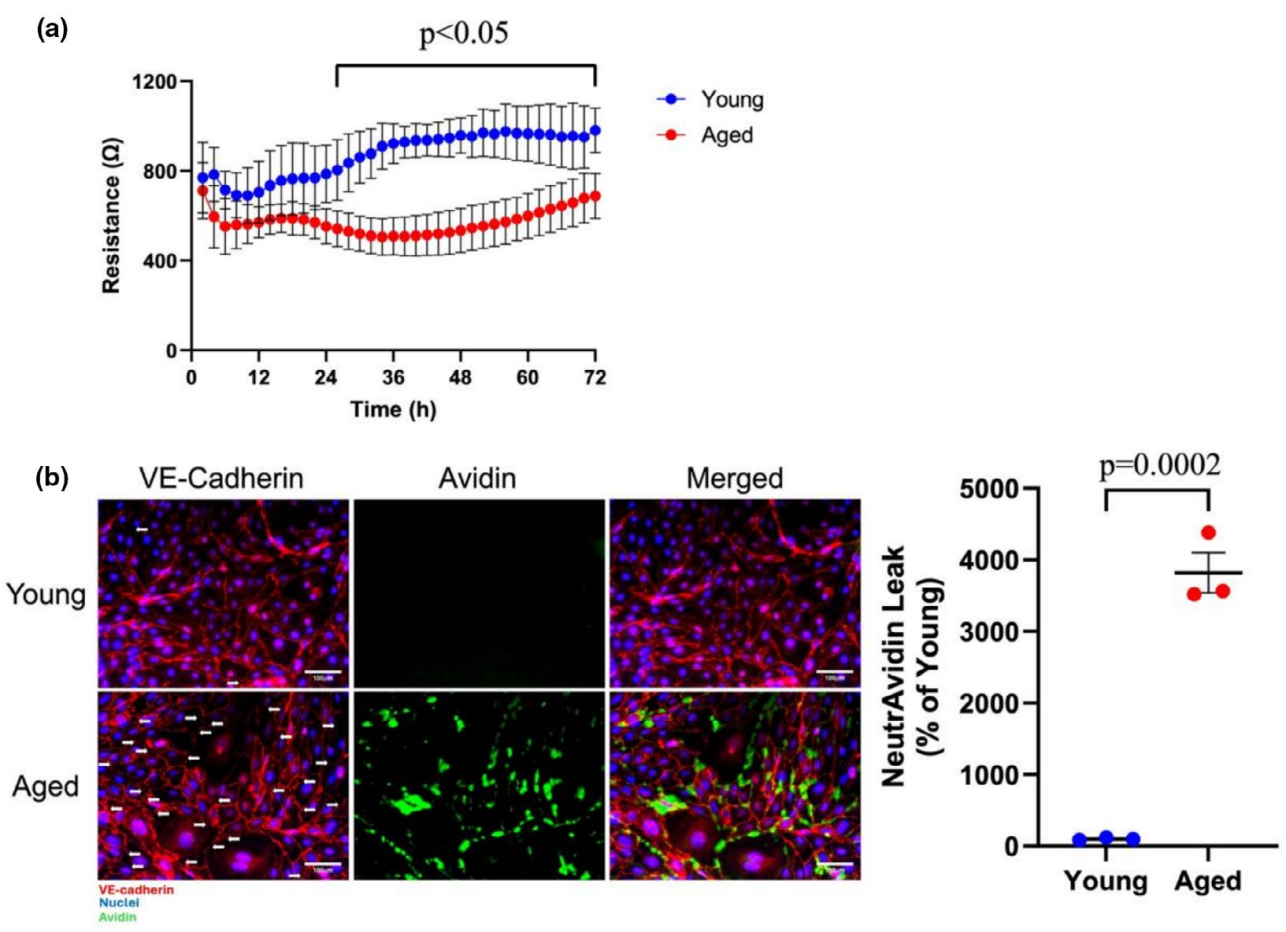
Effect of aging on pulmonary microvascular endothelial cell (PMVEC) permeability under basal conditions. (a) Monolayer resistance was assessed in PMVEC from young and aged mice by electric cell‐substrate impedance sensing (ECIS). Compared to young, PMVEC from aged mice exhibited significantly decreased monolayer resistance 26 h after seeding. (b) Immunofluorescent (IF) staining of vascular endothelial (VE)‐cadherin (RED) and local leak of NeutrAvidin (GREEN) was carried out in PMVEC monolayers from young and aged animals. PMVEC from young mice exhibited continuous VE‐cadherin IF staining around the periphery of the cells, and this was associated with minimal leak. In contrast, PMVEC from aged mice exhibited discontinuous VE‐cadherin IF staining (white arrows) and significantly increased avidin leak, with areas of leak colocalized directly at paracellular regions of VE‐cadherin discontinuity. For ECIS experiments, *n* = 4; Error bars represent standard deviation; Repeated measures two‐way ANOVA. For NeutrAvidin Leak experiments, *n* = 3; Unpaired *t*‐test. Scale bar = 100 μm.

PMVEC isolated from young and aged mice were grown to confluence, and barrier function was assessed by ECIS and the XPerT permeability assay. Following 26 h after plating, the ECIS data revealed that PMVEC from aged animals exhibited a significantly lower overall resistance compared to PMVEC from young animals (Figure 1a). In order to directly examine the relationship between junctional disruption and PMVEC permeability, the XPerT permeability assay was employed concomitantly with VE‐cadherin staining. PMVEC from young animals exhibited minimal avidin leak staining along with continuous cell‐surface VE‐cadherin localization (Figure 1b). In contrast, PMVEC from aged animals exhibited significantly greater avidin staining, which was all co‐localized to paracellular regions with discontinuous VE‐cadherin staining (Figure 1b).

### 3.2 Quantification of junctional proteins in PMVEC from young and aged mice

**FIGURE 2:**
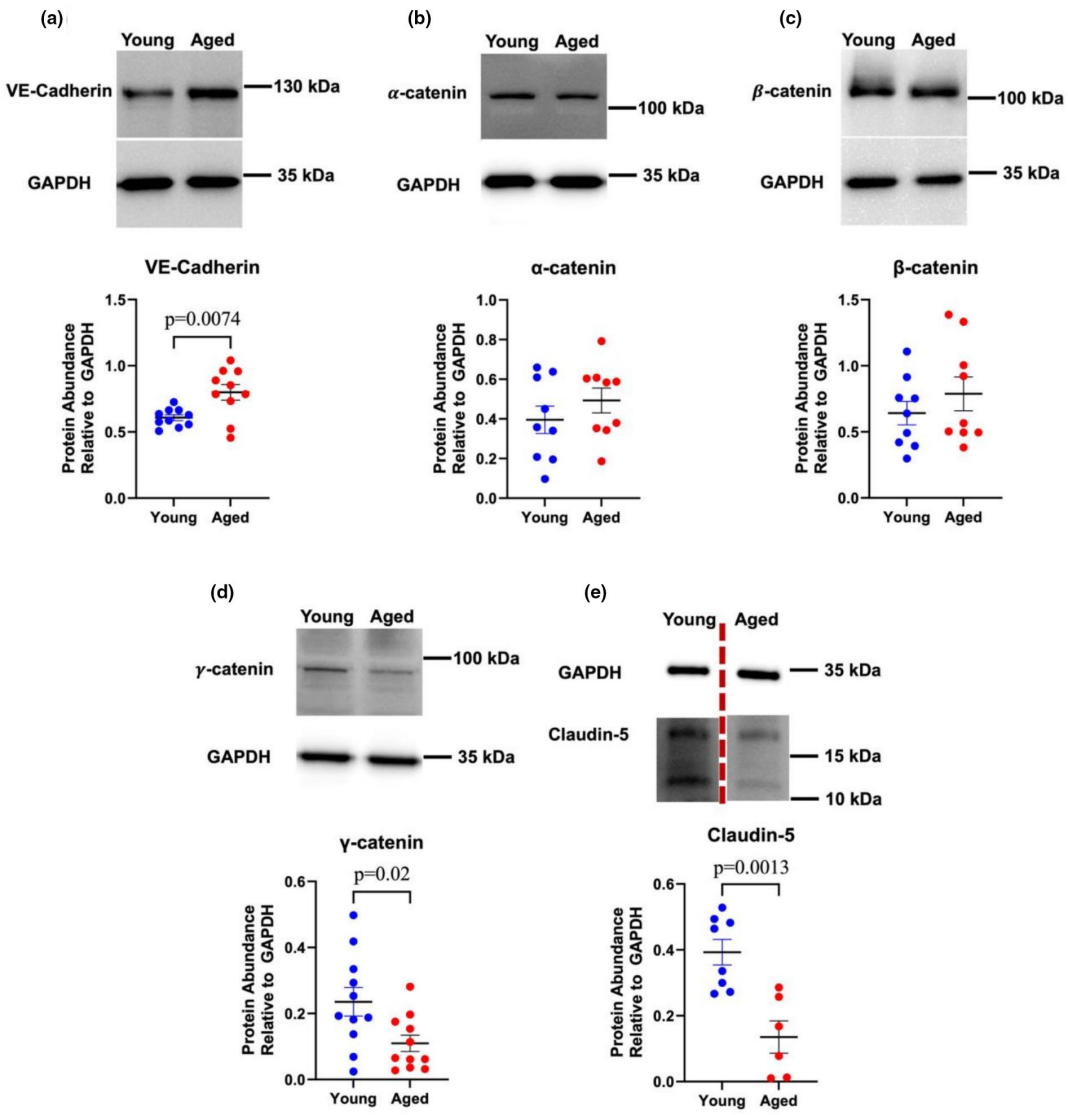
Effect of aging on cell–cell junction protein abundance in pulmonary microvascular endothelial cells (PMVEC). Western blot analyses were performed to quantify the abundance of vascular endothelial (VE)‐cadherin (a), α‐catenin (b), β‐catenin (c), γ‐catenin (d), and claudin‐5 (e). Compared with PMVEC from young mice, PMVEC from aged mice exhibited a significant increase in VE‐cadherin, but a significant reduction in γ‐catenin and claudin‐5. *n* = 6–11; Unpaired *t*‐test. The western blot for claudin‐5 (E) has been spliced to remove an experimental group that was not pertinent to the comparison presented.

To continue to explore the impact of age on PMVEC cell–cell junctional proteins, the abundance of the transmembrane proteins, as well as the adherens junction adapter proteins, was assessed using Western blot analysis under basal conditions. Compared with young, PMVEC from aged mice exhibited significantly greater VE‐cadherin abundance, but lower claudin‐5 and γ‐catenin abundance (Figure 2). No differences were detected in the other adherens junction adapter proteins, α‐catenin and β‐catenin (Figure 2).

### 3.3 Proteomics analysis of PMVEC from young and aged mice

**FIGURE 3:**
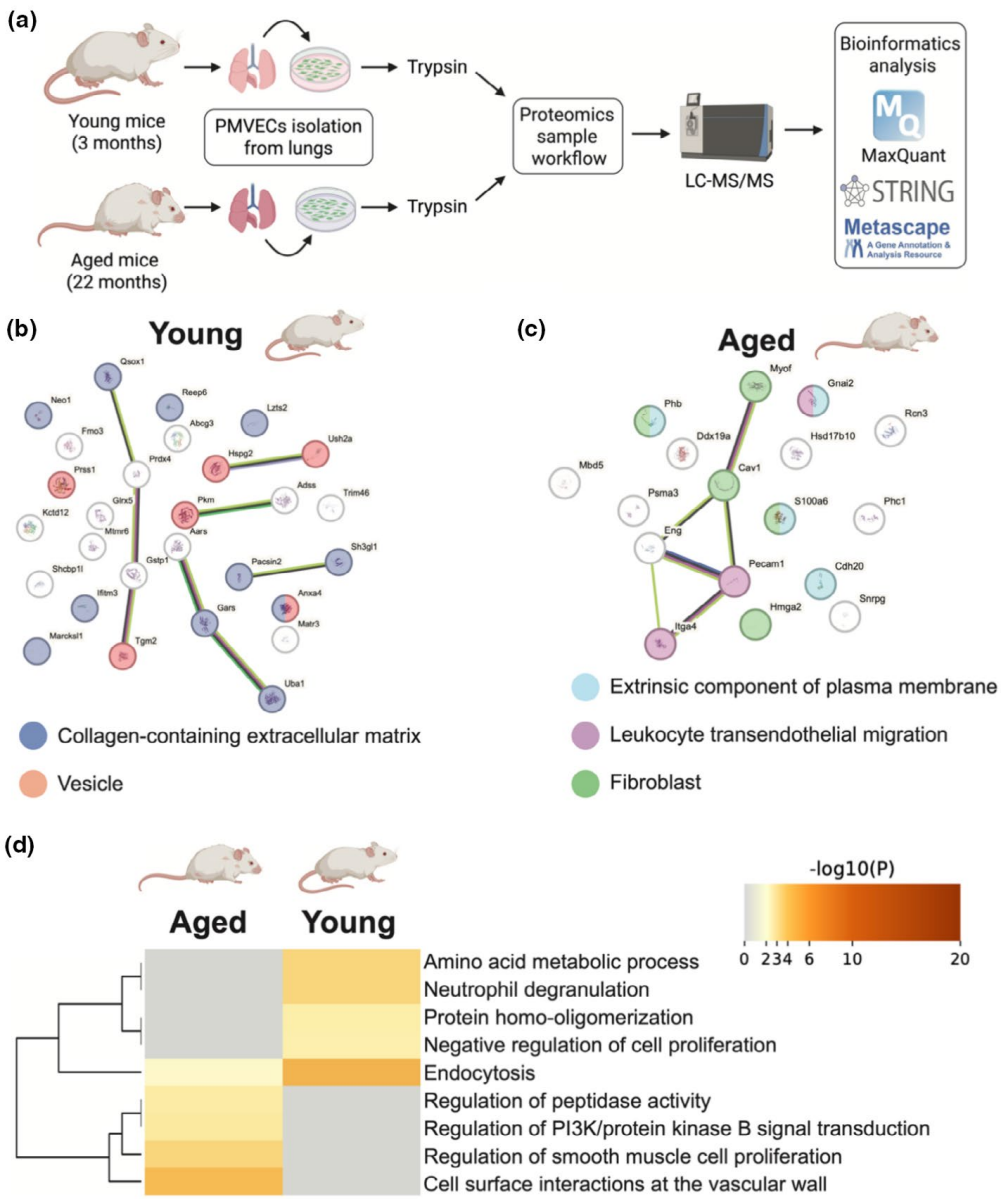
Proteomics analysis of pulmonary microvascular endothelial cells (PMVEC) from young and aged mice. (a) Workflow schematic of proteomics experimental design. PMVEC were isolated from young and aged mice, cultured to confluency in vitro, and lysed for protein collection. Proteins were digested with trypsin, labeled with light or heavy formaldehyde, subjected to LC–MS/MS, and analyzed using MaxQuant. (b and c) STRING‐db analysis (Szklarczyk et al., 2019) of enriched proteins in PMVEC from young and aged mice. Enrichment of collagen‐containing extracellular matrix (DARK BLUE) and vesicle (ORANGE) in young, and extrinsic component of plasma membrane (LIGHT BLUE), leukocyte transendothelial migration (PURPLE) and fibroblasts (GREEN) in aged, are shown in colored spheres. (d) Metascape analysis (Zhou et al., 2019) revealed a number of biological pathways that were altered between PMVEC from young and aged animals, including endocytosis, regulation of peptidase activity, regulation of PI3K/protein kinase B signal transduction, and cell surface interactions at the vascular wall. Schematic graphics used in this figure were created using BioRender. Datasets were derived from *n* = 4 per age group.

Shotgun proteomics analysis was employed to examine global changes in the proteome between PMVEC isolated from young and aged mice (Figure 3a). Using a fold change cutoff of 2, a total of 29 proteins were enriched in PMVEC from young animals, while 17 proteins were enriched in the aged (Table S1). STRING‐db analysis (Szklarczyk et al., 2019) revealed an enrichment of collagen‐containing extracellular matrix and vesicle pathways in the young PMVEC (Figure 3b), and extrinsic component of plasma membrane, leukocyte transendothelial migration, and fibroblast pathways in the aged PMVEC (Figure 3c). Gene ontology analysis revealed many pathways that were significantly differentially enriched in both groups; these included pathways such as endocytosis, regulation of PI3K/protein kinase B signal transduction, regulation of smooth muscle cell proliferation, cell surface interactions at the vascular wall, and cell activation pathways associated with inflammation, such as neutrophil degranulation (Figure 3d). A common feature of each of these pathways is actin cytoskeleton modification or rearrangement (Bogatcheva & Verin, 2008; Jiménez et al., 2000; Tang, 2018; Wu & Chan, 2022; Yadunandanan Nair et al., 2022; Zarbock & Ley, 2008). Additional analysis was conducted on the proteomics dataset, with a less stringent fold change cutoff of 10% variance, in order to extend the search to other proteins that may be relevant to the above pathways. Examination of the significantly differentially enriched proteins revealed 218 proteins enriched in young and 114 proteins enriched in aged; this led to pathways associated with the actin cytoskeleton that appeared in both groups, driven by different proteins enriched in each group (Figure S1). Specifically, these pathways were driven by 23 proteins from the young PMVEC, such as beta actin, actin related protein 2/3 complex subunit 3, and actin related protein 2 (Table S2), and 28 proteins from the aged PMVEC, including actinin alpha 4, destrin, and adducin 1 (Table S3).

### 3.4 Assessment of actin cytoskeleton arrangement in PMVEC from young and aged mice

**FIGURE 4:**
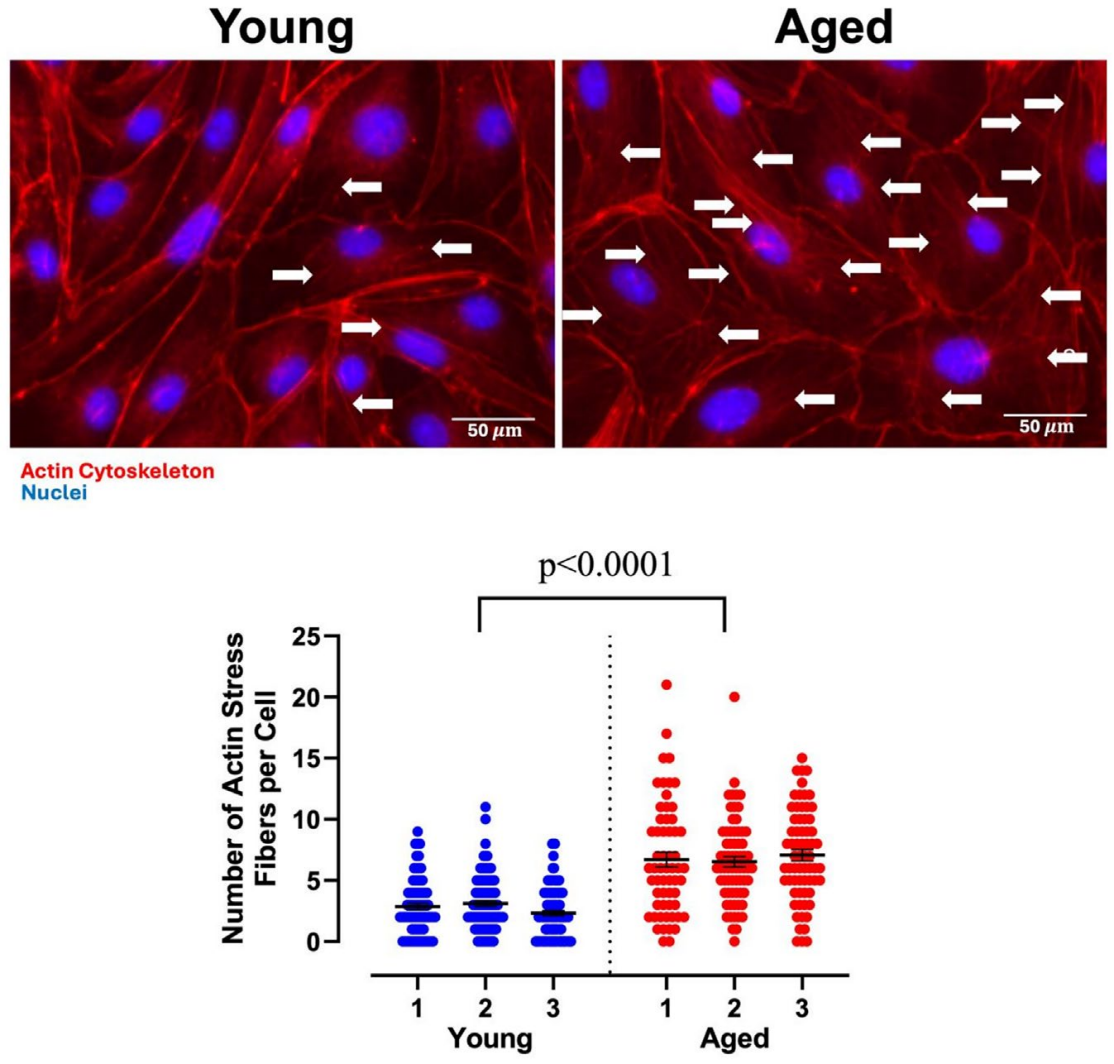
Effect of aging on Actin cytoskeleton organization in pulmonary microvascular endothelial cells (PMVEC). Phalloidin staining was performed to examine the Actin cytoskeleton in PMVEC from young and aged mice. Compared to PMVEC from young animals, which exhibit primarily cortical Actin staining, PMVEC from aged animals has significantly higher stress fiber formation under basal conditions. Above are representative images from *n* = 3 experiments. Arrows indicate Actin stress fibers. Scale bar = 50 μm.

To follow up on the pathways identified in the proteomics analysis associated with alterations in the actin cytoskeleton (Figure 3 and Figure S1), further analysis was conducted to assess the arrangement of the actin cytoskeleton in PMVEC from young and aged mice (Figure 4). This analysis revealed minimal stress fiber formation with strong cortical actin staining in the PMVEC from young animals. In contrast, PMVEC from aged animals exhibited significantly higher stress fiber formation under basal conditions (Figure 4). Additionally, when examining the distribution of stress fiber quantity among the PMVEC, cells with a greater abundance of stress fibers (5 or more quantifiable stress fibers/cell) were predominantly in the aged group; meanwhile, a larger percentage of cells with few to no stress fibers were observed in the young (Figure S2).

### 3.5 Impact of aging on inflammatory mediator expression in PMVEC from young and aged mice

**FIGURE 5:**
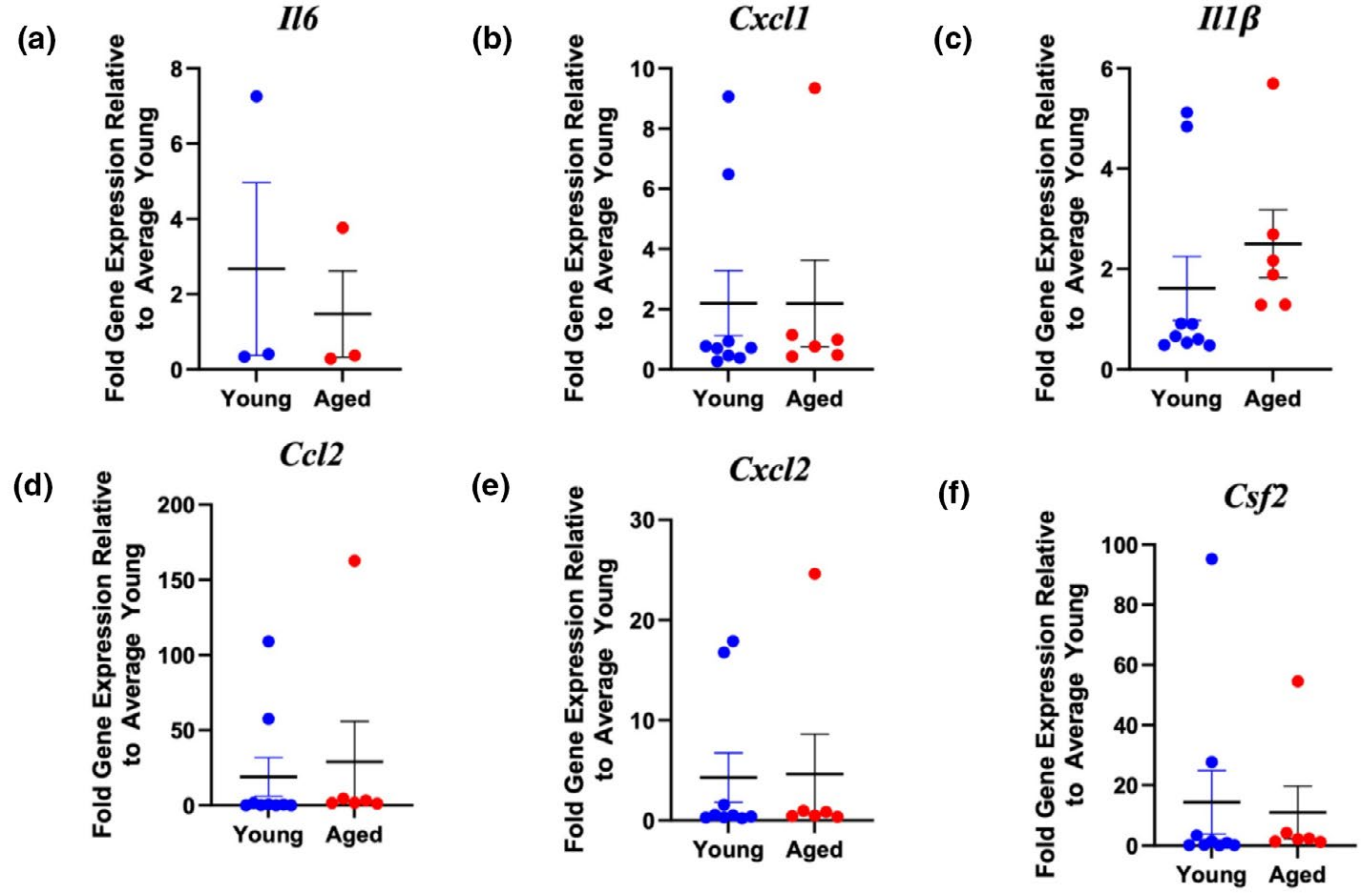
Analysis of proinflammatory cytokine expression in pulmonary microvascular endothelial cells (PMVEC) from young and aged animals under basal conditions. Expression levels of interleukin (*Il*)*6* (a), CXC motif chemokine ligand (*Cxcl*)*1* (b), Il1β (c), C‐C motif chemokine ligand (*ccl*)*2* (d), *Cxcl2* (e), and colony stimulating factor (*Csf*)*2* (f) were assessed by qPCR. No significant differences were observed between young and aged PMVEC across all cytokines. *n* = 3–9; Unpaired *t*‐test.

To follow up on other key findings from the proteomics analysis associated with inflammatory signaling, assessment of inflammation was conducted in the PMVEC. To examine the potential impact of age on the expression of pro‐inflammatory mediators under basal conditions, qPCR analysis was employed to quantify expression of a variety of cytokines and chemokines, including *Il6*, *Cxcl1*, *Il1* β, *Ccl2*, *Cxcl2*, and *Csf2*, in PMVEC isolated from young and aged mice. Across all cytokines, no significant differences were observed between young and aged PMVEC under basal conditions (Figure 5).

### 3.6 Effect of aging on PMVEC cell adhesion molecules

**FIGURE 6:**
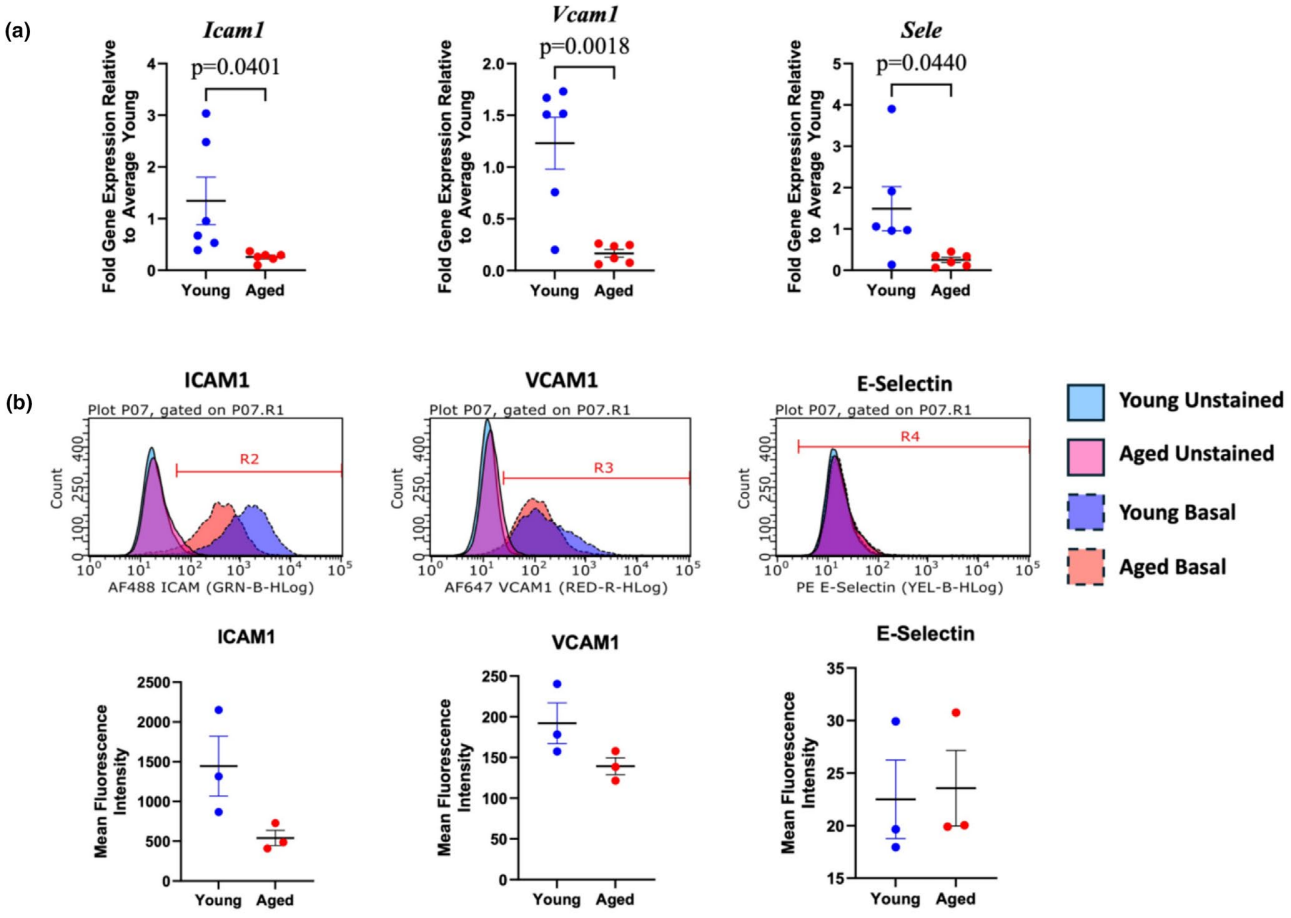
Assessment of cell adhesion molecule expression and cell surface abundance in pulmonary microvascular endothelial cells (PMVEC) from young and aged mice. Intercellular adhesion molecule‐1 (*Icam1*/ICAM1), vascular cell adhesion molecule 1 (*Vcam1*/VCAM1), and E‐selectin (*Sele*) expression and cell surface abundance were assessed by qPCR and flow cytometry, respectively. Relative to young, PMVEC from aged mice exhibited significantly decreased *Icam1*, *Vcam1*, and *Sele* expression (a). While there was a trend towards decreased ICAM1 and VCAM1, no statistically significant differences were observed in cell surface abundance mean fluorescence intensity for the three cell adhesion molecules (b). For qPCR experiments, *n* = 6; for flow cytometry experiments, *n* = 3; Unpaired *t*‐test.

Additional assessment of PMVEC activation was performed through analysis of cell adhesion molecule expression and cell surface abundance. qPCR analysis revealed significantly decreased expression of *Icam1*, *Vcam1*, and *Sele* in PMVEC from aged mice compared to young (Figure 6a). While cell surface presence of ICAM1 and VCAM1 was detected with flow cytometry under basal conditions in both young and aged PMVEC, there was no discernible presence of E‐selectin, as indicated by the overlap of the basal signal with the unstained control cells (Figure 6b). Further, analysis of mean fluorescence intensity revealed that cell surface abundance of all the adhesion molecules was not significantly different between young and aged; notably, there was a trend towards a decrease in ICAM1 and VCAM1 (Figure 6b).

### 3.7 Assessment of the role of aging on PMVEC barrier function under inflammatory conditions

**FIGURE 7:**
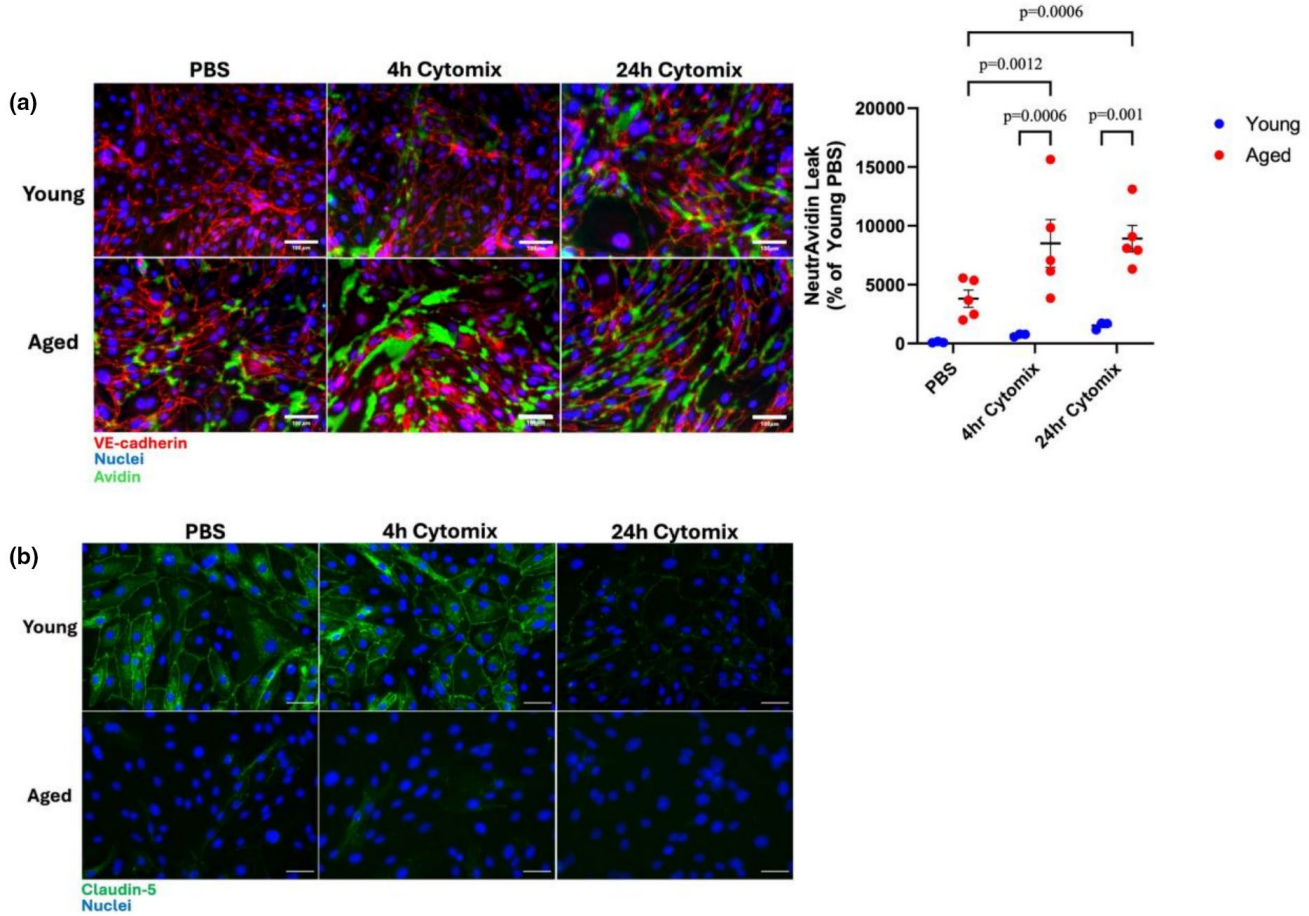
Effect of aging on localized leak and cell–cell adherens and tight junctions in pulmonary microvascular endothelial cells (PMVEC) under inflammatory conditions. (a) Immunofluorescent (IF) staining of vascular endothelial (VE)‐cadherin (RED) and local leak of NeutrAvidin (GREEN) was assessed in PMVEC from young and aged mice stimulated with PBS and cytomix. Compared to PBS, PMVEC from young animals treated with cytomix exhibited disruption of VE‐cadherin and a trend towards increased avidin leak. PMVEC from aged animals had increased leak and VE‐cadherin disruption under basal conditions, which was exacerbated, and significantly increased compared to young, following cytomix stimulation. (b) IF staining of claudin‐5 (GREEN) revealed strong and continuous cell‐surface localization in PMVEC from young mice under basal conditions, with disrupted and decreased staining following cytomix stimulation, particularly at 24 h. PMVEC from aged animals exhibited sparse claudin‐5 cell‐surface localization under control conditions, which persisted following cytomix stimulation. Above are representative images from *n* = 3–5 experiments. For NeutrAvidin Leak experiments, *n* = 3–5; Two‐way ANOVA. Scale bar = 100 μm.

To explore whether the greater basal leak in aged PMVEC led to augmented PMVEC barrier dysfunction under pathological conditions, PMVEC from young and aged mice were treated with PBS (control) or cytomix (pro‐inflammatory), and VE‐cadherin staining and local leak were assessed. While not significant, an increase in permeability in the PMVEC from young animals was observed at 4 h following cytomix treatment, which persisted at 24 h (Figure 7a). Furthermore, the increase in leak was associated with disrupted and discontinuous VE‐cadherin cell‐surface localization (Figure 7a). In aged PMVEC, treatment with cytomix led to a significant increase in avidin leak compared to baseline (Figure 7a). Moreover, compared to young, the PMVEC dysfunction in response to cytomix, including VE‐cadherin disruption and local leak, was significantly increased in the PMVEC isolated from the aged animals (Figure 7a). Assessment of tight junctions was also performed by immunocytochemical analysis of claudin‐5. Similar to VE‐cadherin, PMVEC from young mice exhibited continuous claudin‐5 around the periphery of the cells under basal conditions, with localization appearing to become disrupted as early as 4 h post‐cytomix stimulation (Figure 7b). This disruption persisted and was associated with an apparent loss of claudin‐5 localization in young PMVEC at 24 h post‐cytomix stimulation (Figure 7b). As we have shown previously, PMVEC from the aged animals appeared to have sparse claudin‐5 localization around the periphery of the cells under control conditions, which persisted following cytomix stimulation (Figure 7b) (Manji et al., 2025).

### 3.8 Effect of aging on actin cytoskeleton arrangement in PMVEC under inflammatory conditions

**FIGURE 8:**
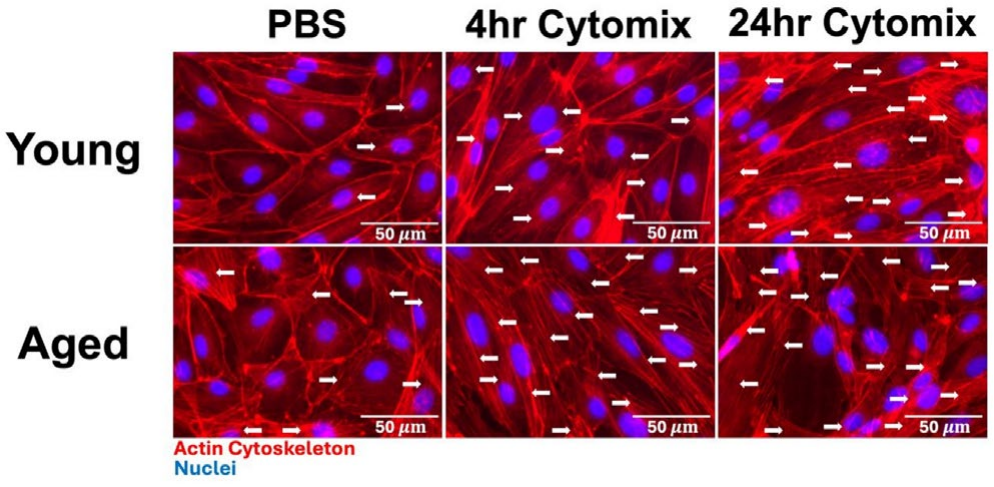
Actin cytoskeleton organization (phalloidin staining) in pulmonary microvascular endothelial cells (PMVEC) from young and aged mice under inflammatory conditions. PMVEC from young animals exhibited increased stress fiber formation beginning at 4 h post‐cytomix stimulation (RED), which persisted at 24 h. In comparison, PMVEC from aged animals exhibited stress fibers under basal conditions with augmented stress fiber formation at both 4 and 24 h post‐cytomix stimulation. Above are representative images from *n* = 3 experiments. Arrows indicate Actin stress fibers. Scale bar = 50 μm.

Given the differences observed in the actin cytoskeleton arrangement in young and aged PMVEC under basal conditions, subsequent analysis was conducted under inflammatory conditions. Just like in Figure 4, PMVEC from the aged mice exhibited stress fiber formation under basal (PBS‐treated) conditions (Figure 8). Similar to what we have shown previously, stress fiber formation began to occur 4 h post‐cytomix stimulation in the PMVEC from young animals, with a further increase being observed after 24 hours (Figure 8) (Arpino et al., 2016). PMVEC from the aged animals exhibited stress fiber formation under basal conditions, which was further augmented following stimulation with cytomix at both the 4‐ and 24‐h time points (Figure 8).

## 4 DISCUSSION

The objective of this study was to investigate the impact of aging on PMVEC barrier function, both under basal and inflammatory conditions. To assess this, we conducted studies in isolated PMVEC from mice at 2–3 months of age and at 18–22 months of age, which correspond to approximately 20‐ and 65‐years of age in humans, respectively (Barros et al., 2021). Overall, our findings supported our general hypothesis that aging was associated with PMVEC barrier dysfunction due to impairments in cell–cell junction integrity. Furthermore, we propose that the deficiencies in PMVEC barrier function with age may be mediated by actin cytoskeletal alterations, particularly the presence of stress fibers. To our knowledge, this is the first in vitro study to demonstrate impaired endothelial barrier function of the pulmonary microvasculature directly with age due to disrupted cell–cell junctions. Our findings may begin to elucidate the underlying mechanisms associated with pulmonary microvascular injury, including augmented permeability, which underpins the worse clinical outcomes with age during lung injury.

The primary outcome of this study was the observation of an increase in permeability in PMVEC monolayers isolated from the aged mice compared to young mice under basal and inflammatory conditions, and its direct association with cell–cell junctional disruption. The use of the XPerT macromolecule permeability assay in the current study, which enables the visualization of localized leak concomitantly with immunocytochemical analysis of junctional proteins, demonstrated that the increased leak with age was directly localized to paracellular regions of VE‐cadherin discontinuity. Furthermore, following treatment with cytomix, the disrupted VE‐cadherin and leak were further exacerbated. Additionally, immunocytochemical analysis of claudin‐5 revealed minimal staining in the PMVEC from aged mice, which persisted following cytomix stimulation. Based on these findings, we conclude that the age‐associated impairment in cell–cell junction integrity was directly contributing to the elevated microvascular permeability.

Other studies in the literature have observed similar findings with aging being associated with endothelial barrier dysfunction and impairments in cell junctions. A number of in vivo animal studies assessing blood–brain barrier permeability following injury demonstrated augmented microvascular permeability, along with disruptions in the cell–cell junctions with age (Cheng et al., 2024; Lee et al., 2012; Shen et al., 2019). Furthermore, additional in vivo studies examining endothelial barrier function in aged rodents under healthy conditions have demonstrated increased vascular permeability within the blood–brain barrier, blood‐retinal barrier, and thoracic aorta, concomitant with reduced junctional protein abundance or disrupted and reduced junctional protein localization (Bake et al., 2009; Chandra et al., 2023; Chan‐Ling et al., 2007; Elahy et al., 2015; Huynh et al., 2011; Liang et al., 2020; Propson et al., 2021; Wen et al., 2020). In vitro studies have also found similar outcomes in primary brain microvascular endothelial cells, human umbilical vein endothelial cells, human aortic endothelial cells, and human umbilical cord blood‐derived endothelial cells (Cheung et al., 2012; Krouwer et al., 2012; Stamatovic et al., 2019; Ting et al., 2023; Venkatesh et al., 2011; Ya et al., 2023). Specific alterations were observed with VE‐cadherin, claudin‐5, occludin, zona occludens (ZO)‐1, and ZO‐2, including disrupted immunofluorescent staining or fragmentation of the proteins (Stamatovic et al., 2019; Venkatesh et al., 2011; Ya et al., 2023), decreased protein abundance (Krouwer et al., 2012; Ting et al., 2023; Venkatesh et al., 2011), and decreased protein localization at the cell junctions (Cheung et al., 2012; Ting et al., 2023; Ya et al., 2023). A more recent study using drug‐induced senescence in primary human lung microvascular endothelial cells demonstrated reduced claudin‐5, ZO‐1, and VE‐cadherin protein abundance and localization at the junctions (Najari Beidokhti et al., 2025). Notably, these previous in vitro studies that have examined endothelial barrier function with age have relied on the induction of senescence to model aging, which may not accurately recapitulate the microenvironment observed in elderly individuals (Cheung et al., 2012; Krouwer et al., 2012; Najari Beidokhti et al., 2025; Stamatovic et al., 2019; Ting et al., 2023; Venkatesh et al., 2011; Ya et al., 2023). Our current work expands upon these previous studies through the use of primary endothelial cells from the microvasculature of the lungs, as well as the fact that these cells were isolated directly from young and aged mice. Furthermore, our in vitro data support the notion that the barrier dysfunction observed with age is intrinsic to the endothelial cells.

While the aforementioned discussion offers insights into the general behavior of aged PMVEC with respect to permeability, our subsequent analysis of inflammation, junctional protein abundance, and proteomics provides some molecular insight into the various proteins and pathways involved in this altered response. Inflammation is well established as a contributor to endothelial cell–cell junction damage, thereby inducing endothelial barrier dysfunction (Aveleira et al., 2010; Clark et al., 2015; Huber et al., 2001; Jayawardena et al., 2023; Liu et al., 2019). Furthermore, healthy aging is associated with elevated markers of senescence and inflammation, including increased cytokine expression and abundance, and increased activation of inflammatory cells; this increase in basal inflammatory signaling with age has been well characterized and is referred to as “inflammaging” (Ferrucci & Fabbri, 2018; Franceschi et al., 2000; Linge et al., 2015; Meyer et al., 1996; Saavedra et al., 2023). However, examining inflammatory signaling in PMVEC, through quantification of inflammatory mediator expression and cell surface abundance of endothelial cell adhesion molecules, revealed no significant differences between young and aged PMVEC. In fact, there was a trend towards significantly lower cell‐surface abundance of certain adhesion molecules, like ICAM1 and VCAM1. It is possible that our model of aging, which utilized animals on a food‐restricted diet and minimized the development of obesity, may have been protective against the development of the senescence‐associated secretory phenotype typically observed with age. Very few studies in the literature assessing aging and pulmonary vascular dysfunction have accounted for obesity in their models, making this a particularly relevant area for future study. Another potential reason for the observed lack of a hyperinflammatory phenotype in our aged PMVEC is the idea that the endothelial cells of the lungs may not be a major source of the heightened baseline inflammation that is typically associated with older age.

With regard to the junctional proteins, surprisingly, we observed a significant increase in VE‐cadherin abundance with age, but a significant reduction in claudin‐5 and γ‐catenin. The inclusion of claudin‐5 and γ‐catenin within cell–cell junctions is thought to occur in mature endothelial junctions (Lampugnani et al., 1995; Liebner et al., 2000; Taddei et al., 2008; Vestweber et al., 2010). Based on this, we speculate that the PMVEC from aged mice have deficiencies in forming mature cell–cell junctions, and to compensate for this deficit, there is an increase in VE‐cadherin abundance. There is evidence to support this; for instance, VE‐cadherin adhesion is known to be the primary adhesion event during vessel development, and VE‐cadherin adhesion is known to promote activation of claudin‐5 expression (Breier et al., 1996; Taddei et al., 2008). The disrupted VE‐cadherin localization at the cell membrane observed in the PMVEC from aged mice may explain the subsequent reduction in proteins like claudin‐5 and γ‐catenin, as these latter proteins rely on the preliminary, early formation of the adherens junction. Collectively, we speculate that with age, PMVEC exhibit deficiencies in the formation of mature and stable junctions, and that elevated inflammatory signaling is not driving the barrier dysfunction observed.

Further interrogation of our proteomics analysis revealed several biological pathways that were altered in the PMVEC, including pathways associated with the actin cytoskeleton. Consistent with this observation, our recent publication, assessing the transcriptomic differences in pulmonary microvascular cells from young and aged mice using single‐cell RNA sequencing (Manji et al., 2025), found this same pathway was also differentially enriched. The actin cytoskeleton is known to play a pivotal role in modulating endothelial barrier function (Dejana & Vestweber, 2013; Hong et al., 2013; Liu et al., 2010; Nelson & Chen, 2003; Wettschureck et al., 2019). The presence of cortical actin is known to enhance barrier function through the generation of intracellular tension, which enhances clustering, assembly, development, and integrity of cell–cell junctions (Dejana & Vestweber, 2013; Hong et al., 2013; Liu et al., 2010; Nelson & Chen, 2003; Oldenburg & De Rooij, 2014). Conversely, actin depolymerization and the formation of actin stress fibers lead to reduced junctional binding within the membrane, ultimately resulting in intercellular gap formation and endothelial barrier dysfunction; importantly, this has been suggested to occur during ARDS (Abadie, 2005; Dudek & Garcia, 2001; Kása et al., 2015; Mikelis et al., 2015; Radeva & Waschke, 2018; Van Buul & Timmerman, 2016; Wettschureck et al., 2019). In the present study, PMVEC from young animals exhibited cortical actin staining with minimal stress fiber formation under basal conditions. Cytomix stimulation induced stress fiber formation, which was consistent with previous work conducted by our group (Arpino et al., 2016). Interestingly, PMVEC from the aged animals exhibited actin stress fiber formation under basal conditions, which was subsequently augmented following stimulation with cytomix. This aligns with the literature, which suggests that aging and senescence are associated with altered actin cytoskeleton protein expression, distribution, and protein–protein interactions, ultimately leading to changes in cytoskeletal dynamics and actin disorganization (Chandra et al., 2024; Jenkins et al., 1989; Kajuluri et al., 2021; Kim et al., 2022; Najari Beidokhti et al., 2025; Nicholson et al., 2018). While further research is warranted, the presence of actin stress fibers may provide molecular insights into the loss of cell–cell junctional integrity and subsequent barrier dysfunction observed in the aged PMVEC. It also provides a putative mechanism for future studies to further pursue and explore as a potential therapeutic target to mitigate age‐related vascular damage and elevated morbidity during lung injury.

Speculating on the clinical implications of our data in humans, we postulate that the endothelial barrier dysfunction observed with age may contribute to augmented morbidity during lung injury. We have previously demonstrated in a mouse model of mechanical ventilation that aging was associated with augmented vascular permeability (Manji et al., 2025). The work from the current study provides insight into differences in the ability of PMVEC from aged mice to reform a barrier once this barrier is disrupted, which is what is required during the formation of an intact monolayer under basal conditions in a cell culture model. These observed alterations may predispose the cells to greater injury following exposure to an additional insult. Specifically, we suggest that the impaired PMVEC cell–cell junctions and weakened barrier function with age lead to an inherent predisposition to pulmonary vascular damage, including following a secondary insult such as sepsis. Furthermore, we suggest that the deficiencies in vascular barrier function could underpin the elevated morbidity and mortality in older patients suffering from lung injury, such as during ARDS.

Our study has some limitations. First, the PMVEC isolated from young and aged mice were all male, which disregards the role of biological sex in mediating barrier function. Second, our in vitro model utilized PMVEC cultured alone, in the absence of other cell types. In particular, the presence of immune cells and/or immune cell factors has been shown to modulate PMVEC barrier function, particularly under inflammatory conditions (Wang et al., 2018). Third, our analysis of junctional protein abundance did not include an assessment of VE‐cadherin phosphorylation status. VE‐cadherin phosphorylation is known to regulate adherens junction stability and endothelial permeability, particularly under inflammatory conditions. Although we observed increased total VE‐cadherin abundance and disrupted localization in aged PMVEC, it is possible that age‐related changes in phosphorylation may contribute to the impaired barrier function. The phosphorylation state of VE‐cadherin and its role in age‐associated endothelial dysfunction will be explored in future studies. Fourth, a limited sample size was used for some experiments (*n* = 3–5). However, this limitation was mitigated by subsequent repeat experiments using different cell populations and cell isolations that validated earlier findings. For example, findings from the permeability experiments under basal conditions (Figure 1) corroborated with data in later studies using cells exposed to PBS (Figure 7). Furthermore, a key strength of our work was the use of multiple complementary techniques that supported similar conclusions, including ECIS and the XPerT assay to assess permeability, and immunocytochemistry and Western blotting to assess junctional integrity. Lastly, our in vitro model relied on static conditions, which are not representative of in vivo vascular flow conditions. Previous studies have indicated that flow promotes endothelial barrier function (McQueen & Warboys, 2023). Despite these limitations, the findings from the current study support our previous work assessing the role of aging on PMVEC barrier function and cell–cell junctions in an animal model of mechanical ventilation. Specifically, aging was shown to promote PMVEC barrier dysfunction, which was associated with transcriptional changes to cell–cell junction proteins, as determined by single‐cell RNA‐sequencing (Manji et al., 2025).

Future studies will aim to address the above limitations. This will include isolating PMVEC from both male and female animals, employing co‐culture systems with other cell types such as neutrophils, developing a system that facilitates the flow of growth media across the luminal side of the PMVEC, and using plasma from patients diagnosed with ARDS as a more pathologically relevant injurious/inflammatory stimulus. Additionally, future studies will delve deeper into the role of aging on the actin cytoskeleton and its modulation of PMVEC barrier function, including investigating the efficacy of known treatments or inhibitors of actin stress fiber formation to elucidate the effects on cell–cell junction disruption and barrier dysfunction.

In summary, this study aimed to assess the effect of aging on PMVEC barrier function, under both basal and inflammatory conditions. Our overall conclusion is that aging was associated with heightened PMVEC barrier dysfunction due to impaired cell–cell junctions associated with augmented actin cytoskeleton disorganization. These findings offer insights into microvascular‐specific mechanisms underlying the increase in age‐related susceptibility during lung injury and provide possible avenues, including those associated with actin cytoskeleton disorganization, for future research to target and evaluate for therapeutic potential.

## AUTHOR CONTRIBUTIONS

A.M., L.W., C.P., P.P., S.Y., and A.D., performed experiments; A.M., C.P., P.P., S.Y., A.D., D.Y., and S.E.G., analyzed data; A.M., P.P., S.Y., R.A.W.V., A.D., E.K.P., and S.E.G. interpreted results of experiments; A.M., and A.D. prepared figures; A.M., R.A.W.V., A.D., and S.E.G. drafted the manuscript; A.M., A.D., R.A.W.V., S.M., and S.E.G., edited and revised the manuscript; A.M., L.W., C.P., S.M., P.P., S.Y., E.P., R.A.W.V., A.D., D.Y., and S.E.G., approved the final version of the manuscript; A.M., S.M., and S.E.G., conceived and designed the research.

## FUNDING INFORMATION

This study was financially supported by the Canadian Institutes of Health Research (#PRR‐177919, #PJT‐185943), London Health Sciences Centre Research Institute (#IRF‐12‐22), Western University (#50658), the Ontario Graduate Scholarship, and the Dean's Research Scholarship.

## CONFLICT OF INTEREST STATEMENT

The authors have declared that no conflict of interest exists.

## ETHICS STATEMENT

All animal procedures were approved by the Western University Animal Care Committee (Approval #2020‐054) in accordance with the Canadian Council on Animal Care Guidelines.

## Supporting information


Appendix S1.

Figures S1–S2.

Table S1.

Table S2.

Table S3.

## Data Availability

The proteomics datasets are available in the PRIDE repository (project accession: PXD065409). All additional data can be made available from the corresponding author upon reasonable request.
