# Supplementary material for: The role of aging on endothelial cell–cell junctions and pulmonary microvascular permeability in male mice
Source: Physiol Rep. 2025 Dec 19;13(24):e70686. doi: 10.14814/phy2.70686 (PMC12717451; doi:10.14814/phy2.70686)
Supplement: Supplementary file 1 — Appendix S1. [file PHY2-13-e70686-s002.docx]

**Pulmonary microvascular endothelial cell (PMVEC) isolation**

Briefly, excised lungs were finely minced, digested with collagenase type 2 (Worthington-Biochem LS004176), and the resulting suspension incubated with magnetic microbeads coated with anti‑CD31 (platelet endothelial cell adhesion molecule) antibody (Miltenyi Biotec 130-097-418, RRID: AB_2814657). Bead‑bound PMVEC were isolated, washed, and resuspended in complete Dulbecco’s Modified Eagle Medium (DMEM; Gibco 11885084, 1 g/L D‑glucose, L‑glutamine, 110 mg/L sodium pyruvate, phenol red) supplemented with 20% fetal bovine serum (FBS; Gibco 12483‑020), Penicillin‑Streptomycin (Gibco 15140122), and 4‑(2‑hydroxyethyl)piperazine‑1‑ethane‑sulfonic acid (HEPES; Gibco 15630‑080). Cells were seeded onto gelatin (Granular Laboratory grade Gelatin 100 Bloom G-7, 74489 Fisher Scientific Company)‑coated flasks (Falcon tissue culture treated T-25 flask, Fischer Scientific Company 1012610) and expanded to ~90 % confluence. Purity was confirmed by staining with antibodies against CD31 (BioLegend 102422), CD34 (BioLegend 128/610), CD146 (BioLegend 134706), and CD202b (BioLegend 124010), conjugated to Pacific Blue, Phycoerythrin, Fluorescein Isothiocyanate, or Allophycocyanin, respectively (VWR Scientific Inc., Radnor, PA), and analyzed on an easyCyte Guava 12HT flow cytometer (Millipore, Billerica, MA, USA). This protocol consistently yielded 99% homogeneous PMVEC populations.

**High performance liquid chromatography (HPLC), mass spectrometry (MS), and bioinformatics analysis for Proteomics Data**

A BCA assay was used to calculate protein concentrations for each sample, according to the manufacturer’s instructions (ThermoFisher Scientific 23227). Total protein (500 µg) from each sample was removed for analysis. Samples were topped up to 1 mL with lysis buffer, followed by the addition of 500 µL of 6M GuHCl containing dithiothreitol (DTT; 5 mM) and incubation for 1 hour at 37°C to reduce disulphide bonds. Free thiols on cysteines were blocked by adding 15 mM of iodoacetamide for 25 minutes in the dark at room temperature. The thiol blocking reaction was quenched by adding an additional 5mM of DTT.

Samples were labeled with either light (control) or isotopically heavy formaldehyde (40 mM; VWD; Cambridge Isotope Laboratories, Inc. DLM-805-PK). This was followed by addition of sodium cyanoborohydride to a final concentration of 20 mM. The pH was adjusted to 6.5 before incubation overnight at 37°C. The next day, an additional 10 mM of light or heavy formaldehyde was added, followed by 10 mM of sodium cyanoborohydride. Samples were incubated at 37°C for 1 hour. After incubation, samples were transferred into a 50 ml Falcon tube with 50 ml of 8:1 ice-cold acetone methanol. Samples were incubated at -20°C for 4 hours. Samples were centrifuged at 9,000 x g for 15 minutes, then washed in 100% methanol 3 times. Following the last wash, the supernatant was discarded. Samples were air-dried in a fume hood for 5 minutes to remove residual methanol. Samples were then resuspended in 200 µl of 200 µM NaOH, and transferred to a 1.5 ml low-binding protein tube before vortexing for 5 minutes. After the protein pellet was completely resuspended, 300 µl of 200 mM HEPES buffer was added. This was followed by the addition of 50 µg of mass spec-grade trypsin. The pH was adjusted to ~8 before incubation overnight at 37°C. Samples were then acidified to a pH of less than 3 with trifluoroacetic acid (TFA) and stored at 4°C overnight for SEP PAK (c18) clean up (Waters WAT020515). Conditioning solution (3mL; 90% v/v methanol, 0.1% TFA v/v 10% v/v high performance liquid chromatography [HPLC] water) was passed through the solid phase extraction (SPE), followed by 2 mL of load solution (100% HPLC water, 0.1% v/v TFA). The sample was loaded into the SPE column followed by 1 mL of additional load solution. Samples were desalted by passing 3 mL of desalt solution (5% v/v. methanol, 95% v/v HPLC water, 0.15 TFA) across the SPE, followed by sample collection into a new tube by passing 1 mL of elution solution (50% v/v HPLC water, 50% v/v acetonitrile, 0.1% TFA). Samples were frozen at -80 before submission for LCMS.

**High performance liquid chromatography (HPLC) and mass spectrometry (MS)**

As we have described previously, tryptic peptides were analyzed on an Orbitrap Fusion Lumos Tribrid mass spectrometer (Thermo Scientific) operated with Xcalibur (version 4.4.16.14) and coupled to a Thermo Scientific Easy-nLC (nanoflow Liquid Chromatography) 1200 system (1–4). A total mass of 2 μg tryptic peptides were loaded onto a C18 trap (75 um x 2 cm; Acclaim PepMap 100, P/N 164946; ThermoScientific) at a flow rate of 2 µL/min of solvent A (0.1% formic acid in LC-MS grade water). Peptides were eluted using a 120 min gradient from 5 to 40% (5% to 28% in 105 min followed by an increase to 40% B in 15 min) of solvent B (0.1% formic acid in 80% LC-MS grade acetonitrile) at a flow rate of 0.3 μL/min and separated on a C18 analytical column (75 um x 50 cm; PepMap RSLC C18; P/N ES803; ThermoScientific). Peptides were then electrosprayed using 2.1 kV into the ion transfer tube (300°C) of the Orbitrap Lumos operating in positive mode. The Orbitrap first performed a full MS scan at a resolution of 120,000 FWHM to detect the precursor ion having a *m*/*z* between 375 and 1575 and a +2 to +7 charge. The Orbitrap AGC (Auto Gain Control) and the maximum injection time were set at 4e5 and 50 ms, respectively. The Orbitrap was operated using the top speed mode with a 3 second cycle time for precursor selection. The most intense precursor ions presenting a peptidic isotopic profile and having an intensity threshold of at least 5,000 were isolated using the quadrupole and fragmented with HCD (30% collision energy) in the ion routing multipole. The fragment ions (MS^2^) were analyzed in the ion trap at a rapid scan rate. The AGC and the maximum injection time were set at 1e4 and 35 ms, respectively, for the ion trap. Dynamic exclusion was enabled for 45 seconds to avoid acquisition of the same precursor ion having a similar *m/z* (plus or minus 10 ppm).

**Proteomic data and bioinformatics analysis**

Spectra data obtained during mass spectrometry were matched to peptide sequences from a mouse FASTA reference file obtained from Uniprot on February 24^th^, 2022, using MaxQuant (v1.6.0.1). MaxQuant settings were set to default except for the following: variable modifications included oxidation (M), acetyl (N-term), deamidation (NQ); label-free quantification was turned on; first search peptide tolerance was set to 10 under the Orbitrap settings; the identifier rule for the FASTA file was set to the Uniprot; maximum peptide mass was set to 6,600 Daltons; minimum peptide length was set to 5 amino acids; match between runs was turned on. Lists containing the identified proteins and peptides were returned and used for subsequent analysis. Gene Ontology pathway analysis was performed using Metascape (5). Only proteins with a Q-value less than 0.05 between groups were included. Analyses were performed with a fold change cut-off of 2, as well as a less stringent cut-off of 1.1.

**Quantification of actin stress fibers in PMVEC under basal conditions**

Following image acquisition, the images were imported into ImageJ for analysis. Images were converted to 8-bit, background subtraction was performed using a rolling ball radius of 100 pixels, and contrast was enhanced using the “Enhance Contrast” function at a 0.3% saturation with the “Normalize” option checked. The “Straight Line” tool was selected with a line width of 2 pixels, and a line was drawn across each cell in the field of view perpendicular to the observed stress fibers. If fibers appeared random for a given cell, a consistent orientation was chosen. Cells were ignored if they appeared to be dividing (multiple nuclei observed), if they were overly confluent, or if they were partially excluded from the field of view. The “Plot Profile” function was performed, which allowed for the visualization of the intensity peaks where stress fibers crossed the drawn line. Manual counting of distinct peaks in the intensity plot was performed to quantify stress fibers. Data shown were the number of actin stress fibers quantified per cell for all cells. A sample example using the aged PMVEC is included below.


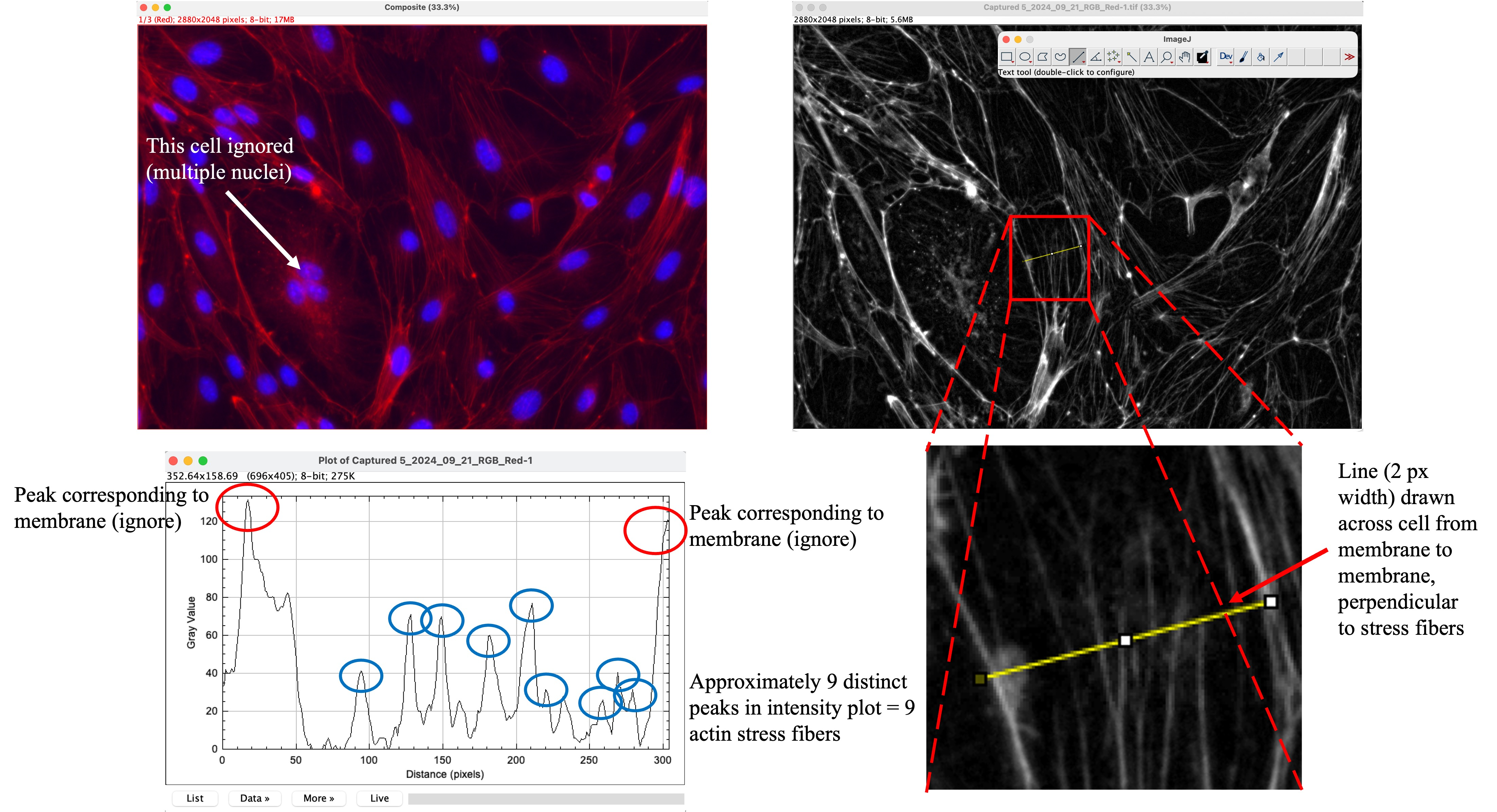


1. Kapilan A, et al. N-terminomics and proteomics analysis of Calpain-2 reveal key proteolytic processing of metabolic and cell adhesion proteins. *Protein Sci Publ Protein Soc*. 2025;34(5):e70144.

2. Kulle A, et al. Alveolar macrophage function is impaired following inhalation of berry e-cigarette vapor. *Proc Natl Acad Sci U S A*. 2024;121(40):e2406294121.

3. Krawetz RJ, et al. Mesenchymal progenitor cells from non-inflamed versus inflamed synovium post-ACL injury present with distinct phenotypes and cartilage regeneration capacity. *Stem Cell Res Ther*. 2023;14(1):168.

4. Das N, et al. Proteomics Analysis of Tears and Saliva From Sjogren’s Syndrome Patients. *Front Pharmacol*. 2021;12:787193.

5. Zhou Y, et al. Metascape provides a biologist-oriented resource for the analysis of systems-level datasets. *Nat Commun*. 2019;10(1):1523.
