# Supplementary material for: The role of aging on endothelial cell–cell junctions and pulmonary microvascular permeability in male mice
Source: Physiol Rep. 2025 Dec 19;13(24):e70686. doi: 10.14814/phy2.70686 (PMC12717451; doi:10.14814/phy2.70686)
Supplement: Supplementary file 2 — Figures S1–S2. [file PHY2-13-e70686-s004.docx]

**Supplemental Figures**


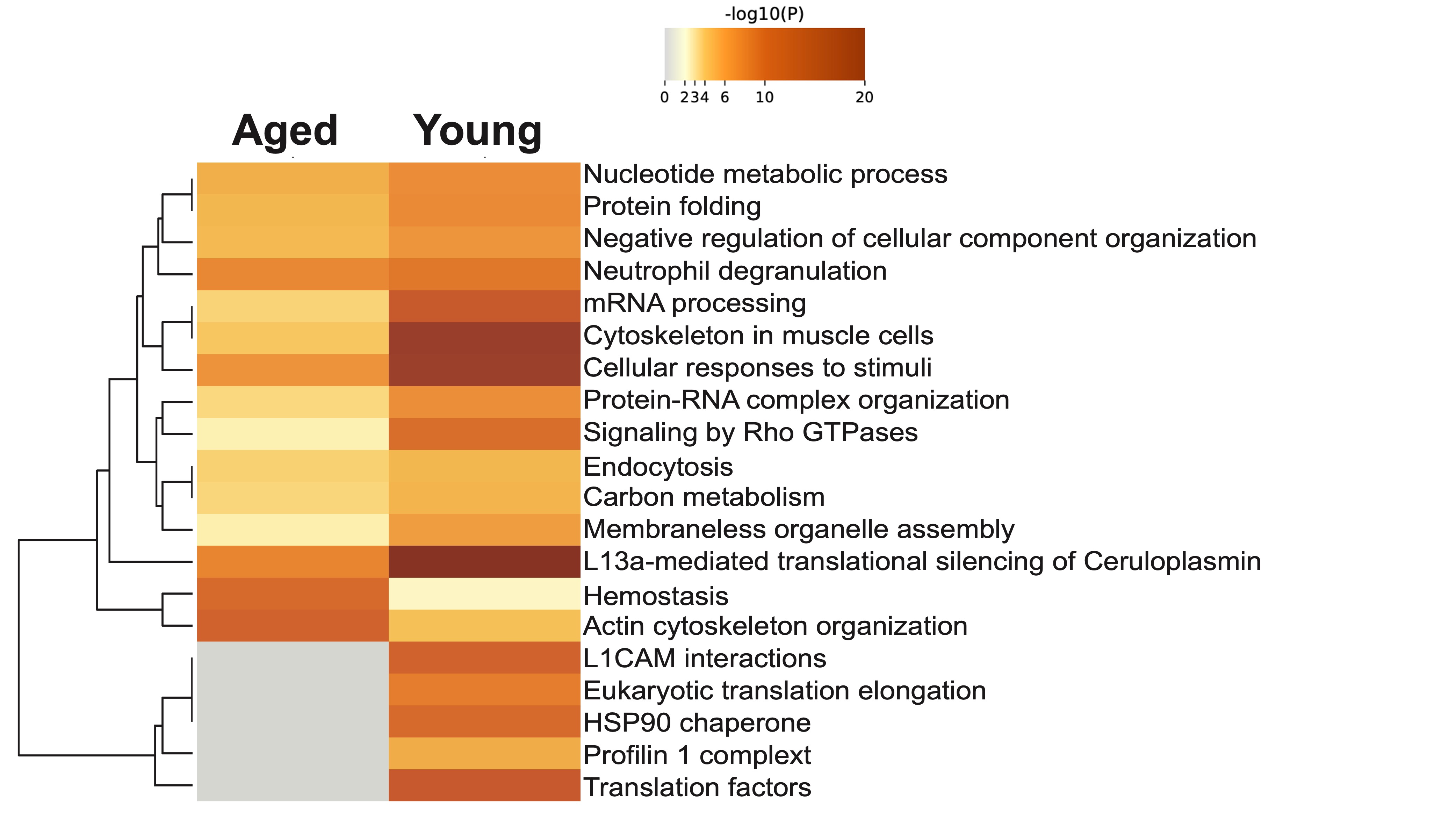


**Supplementary Figure 1:** Metascape analysis of the effect of age on the proteome of pulmonary microvascular endothelial cells (PMVEC). Accumulative hypergeometric p-values and enrichment factors were calculated and used for filtering as performed as a two-sided analysis. Remaining significant terms were then hierarchically clustered into a tree based on Kappa-statistical similarities among their gene's memberships. Then, 0.3 kappa score was applied as the threshold to cast the tree into term clusters. The analysis revealed a number of biological pathways that were altered between PMVEC from young and aged mice, including mRNA processing and translation, HSP90 chaperone signaling, neutrophil degranulation, and actin cytoskeleton organization.

**Supplementary Figure 2:** Histogram of the distribution of stress fiber quantity in pulmonary microvascular endothelial cells (PMVEC) isolated from young and aged mice. A large percentage of PMVEC from young mice exhibit few to no stress fibers. In contrast, PMVEC from aged mice display a large distribution of stress fiber quantity, with a large percentage of cells exhibiting a high abundance (5 or more) of stress fibers.
