# Supplementary material for: The role of aging on endothelial cell–cell junctions and pulmonary microvascular permeability in male mice
Source: Physiol Rep. 2025 Dec 19;13(24):e70686. doi: 10.14814/phy2.70686 (PMC12717451; doi:10.14814/phy2.70686)
Supplement: Supplementary file 3 — Table S1. [file PHY2-13-e70686-s005.pdf]

Supplementary Table 1 | Shotgun proteomics from aged 22 months versus young 3 months pulmonary microvascular endothelial cells (PMVECs).

Peptide sequences were identified from the mouse UniProt protein database with appended standard laboratory and common contamination protein entries and reverse decoy sequences using the Andromeda algorithm as implemented in the MaxQuant software package v1.6.0.1, using a peptide FDR of 0.01. Search parameters included a mass tolerance of 1 ppm for the parent ion and 0.5 Da for the fragment ions, carbamidomethylation of cysteine residues (+57.021464 Da), variable N-terminal modification by acetylation (+42.010565). Database searches were not constrained by enzyme specificity, but limited to a maximal length of 25 residues. The table shows unique identified peptides as listed in the "modificationSpecificPeptides" output file of MaxQuant. Peptide sequences matching reverse or contaminant entries were removed. Mass: peptide mass; charges: precursor charge states of associated MS/MS spectra; PEP: Andromeda Posterior Error Probability.

Proteins	Gene Names	Protein Names	Potential Contaminant	Ratio H/L normalized	Intensity 1 (L) young	Intensity 1 (H) aged	Intensity 2 (L) young	Intensity 2 (H) aged	Intensity 3 (L) young	Intensity 3 (H) aged
Q792Z0;Q792Y8;Q9Z1R9;Q9	rss3;Gm10334;Prss1;Try4;Try5;Try10;Prss	Anionic trypsin-2		0.042526	23874000	490820	45539000	923290	18143000	784520
A0A494BB51;A0A494BB76;C	Lzts2	Leucine zipper putative tumor suppressor 2		0.047953	33112000	2951800	55266000	15154000	18041000	577700
O08807;B1AZS9	Prdx4	Peroxiredoxin-4		0.0786	5191300	195100	3947800	272630	13729000	649060
Q99P81;D3Z0Z4	Abcg3	ATP-binding cassette sub-family G member 3		0.10113	0	0	14587000	154500	980100	549040
Q3TC52;D3YXA6;Q7TNM2;E9	Trim46	Tripartite motif-containing protein 46		0.11612	0	0	120310000	1780700	762700	556910
CON__P00761;Q9CPN9			+	0.12621	79782000	3006100	185140000	8294100	221470000	12431000
Q2QI47;Q2QI47-3	Ush2A	Usherin		0.12912	4897400	218870	7487100	401800	0	0
J3QQ47;Q3TTP0	Shcbp1l	SHC SH2 domain-binding protein 1-like protein		0.15895	7300800	365420	13391000	1261700	0	0
Q9WVE8;A0A2R8W6S4;A0A	Pacsin2	rotein kinase C and casein kinase substrate in neurons protein 2		0.1677	3970100	243950	7825200	807430	7117400	1007700
A0A1Y7VN70;Q80Y14	Glrx5	Glutaredoxin-related protein 5, mitochondrial		0.18569	2072500	290690	6780900	721540	0	0
P28667	Marcksl1	MARCKS-related protein		0.19681	0	0	0	0	3059800	1083500
Q8VE11;Q8VE11-2	Mtmr6	Myotubularin-related protein 6		0.21453	17044000	1486500	0	0	0	0
D3YTR2;D3YTR1	Reep6			0.26168	2036100	31748	0	0	23446000	23879000
Q8BND5;Q8BND5-3;Q8BND	Qsox1	Sulfhydryl oxidase 1		0.26272	1379100	146780	2067000	304190	0	0
Q9CQW9	Ifitm3	Interferon-induced transmembrane protein 3		0.31897	0	0	1602900	3428100	11335000	2582500
Q80UG5-3;A2A6U3;Q80UG5	09-Sep	Septin-9		0.41688	2238400	25701	10273000	285220	922240	318800
P97501	Fmo3	Dimethylaniline monooxygenase [N-oxide-forming] 3		0.41946	1799300	345390	3944000	1118500	0	0
Q8K310;A0A494BAZ2;A0A49	Matr3	Matrin-3		0.42156	0	0	3502700	1395700	4369200	563270
Q9CZD3	Gars	Glycine--tRNA ligase		0.42385	428670	141470	1182500	519440	13415000	0
A0A0N4SW89;P97429;S4R1	Anxa4	Annexin A4;Annexin		0.42487	2653900	994510	13890000	6332000	5214600	2853400
P52480;P52480-2;A0A1L1SU	Pkm	Pyruvate kinase PKM		0.4255	3019000	228390	12158000	10075000	91883000	8918300
P19157;A0A494B908;A0A49	Gstp1;Gstp2	Glutathione S-transferase P 1;Glutathione S-transferase P 2		0.42842	36836000	8650500	79428000	29980000	24571000	18494000
Q02053	Uba1	Ubiquitin-like modifier-activating enzyme 1		0.4295	10384000	6154200	0	0	0	0
Q7TQG5;E9QK04;P97798-5;	Neo1	Neogenin		0.4328	0	0	2766100	2069000	2504100	493940
E9PZ16;B1B0C7;Q05793;A0A	Hspg2	ane-specific heparan sulfate proteoglycan core protein;Endorepellin;LG3 peptide		0.43692	22501000	2452100	24550000	80473000	63186000	14574000
P46664	Adss	Adenylosuccinate synthetase isozyme 2		0.46702	0	0	2688800	1577800	2898200	932500
Q8BGQ7	Aars	Alanine--tRNA ligase, cytoplasmic		0.47423	2332100	509110	3970200	1614200	0	0
P21981;G3UXE8;REV__P024	Tgm2	Protein-glutamine gamma-glutamyltransferase 2		0.48483	14032000	1667900	11016000	1930900	4844900	3137200
A0A0R4J2B2;Q6WVG3	Kctd12	BTB/POZ domain-containing protein KCTD12		0.49302	3774300	952520	8924600	2565500	5836500	1623100
Q62419;A0A3B2W7K0;Q8BX	Sh3gl1;Sh3gl2	Endophilin-A2;Endophilin-A1		0.49739	2878900	712130	3509900	1414100	0	0
P45376	Akr1b1	Aldose reductase		0.51941	2172400	568020	12190000	6087200	3255600	2206500
F8WJK8;Q99L47;E9Q1X9;E9	St13	Hsc70-interacting protein		0.53073	3167100	718600	9050800	4976700	5672900	3562400
G5E850;P56395;E0CY88	Cyb5a	Cytochrome b5		0.55808	5483600	1749900	14951000	5055000	7412700	4443900
A0A494BAX5;A0A498WGK2;	Nars	Asparagine--tRNA ligase, cytoplasmic		0.57715	1382700	853020	3359600	2078400	0	0
P26231;A0A494BAD0;E0CXB	Ctnna1;Ctnna2	Catenin alpha-1;Catenin alpha-2		0.5861	2086700	1995000	0	0	0	0
E9PYH3;Q8BWU8	Etnppl	Ethanolamine-phosphate phospho-lyase		0.59448	450300	422810	12211000	1472500	806590	651590

B7ZNU9;Q61235	Sntb2	Beta-2-syntrophin	0.59949	333310	131340	886700	478050	701950	688520
Q9D1A2;A0A494B9S3;A0A49	Cndp2	Cytosolic non-specific dipeptidase	0.602	5949800	2464200	7134900	3063900	12249000	7644000
P51150;A0A0N4SVR6;A0A0N	Rab7a	Ras-related protein Rab-7a	0.60278	2553300	895440	8671000	4813900	1959400	1181600
A0A3B2WCD8;Q3U0V1;A0A3	Khsrp	Far upstream element-binding protein 2	0.60827	0	0	1704200	1858300	2390100	960510
Q9CQ75	Ndufa2	ADH dehydrogenase [ubiquinone] 1 alpha subcomplex subunit 2	0.6088	0	0	1272100	1145000	2056700	601700
P26040	Ezr	Ezrin	0.60979	0	0	2022900	1057600	1654500	1103900
Q9DCN2-2;Q9DCN2;F2Z456	Cyb5r3	5 reductase 3 membrane-bound form;NADH-cytochrome b5 reductase 3 soluble form;	0.61686	3558900	1717200	6148100	3125800	2125000	1538300
A0A286YDT5;G5E8Q8	Adgrf5		0.61923	1514200	396720	3480300	2046300	0	0
B1AWE0;Q6PFA2;B1AWE1;B	Clta	Clathrin light chain A	0.62182	812080	396720	3549000	2082500	0	0
H7BXC3;P17751;P17751-2	Tpi1	Triosephosphate isomerase	0.62963	12011000	3960700	26878000	13025000	14867000	11111000
P60335	Pcbp1	Poly(rC)-binding protein 1	0.63189	2304100	815640	5156100	2889400	0	0
A0A494B9X6;A0A494BAB1;C	Gsto1	Glutathione S-transferase omega-1	0.63693	2253300	573690	0	0	2820300	1140900
O55125	Nipsnap1	Protein NipSnap homolog 1	0.64508	231370	157520	600570	301880	657560	433920
P97807-2;P97807	Fh	Fumarate hydratase, mitochondrial	0.6455	345950	210520	1014200	630810	1257600	979190
P14733	Lmnb1	Lamin-B1	0.65432	2020000	943890	5623800	5959200	961100	450830
Q6X632	Gpr75	Probable G-protein coupled receptor 75	0.65604	1180000	698440	2152400	1921500	2476700	1126400
Q9JKB3-2;Q9JKB3	Ybx3	Y-box-binding protein 3	0.65886	0	0	5254100	2461400	0	0
H3BKM0;Q9DBG3;Q9DBG3-2	Ap2b1	AP-2 complex subunit beta	0.66575	0	0	519300	310220	814280	259890
A0A498WGD8;Q8CDN6;A0A4	Txnl1	Thioredoxin-like protein 1	0.66686	467920	188810	2769100	3046900	3669100	4930700
Q9Z2X1;Q9Z2X1-2;J3QMT0;J	Hnrnpf	ibonucleoprotein F;Heterogeneous nuclear ribonucleoprotein F, N-terminally processe	0.66919	2985700	2115700	27926000	18326000	17685000	11519000
Q8BGD9;B2RWE8	Eif4b	Eukaryotic translation initiation factor 4B	0.67027	21485000	6100000	24741000	12162000	13539000	22444000
P50516;P50516-2;D3YWH3;D	Atp6v1a	V-type proton ATPase catalytic subunit A	0.67028	680190	349570	4458000	2161000	3188900	2645100
F6WEU2;A0A338P6M1;A0A3	Gm7873		0.67324	3550600	1226400	8150700	4151000	5525200	3505600
E9Q3X0;Q9EQK5;D3Z2N7	Mvp	Major vault protein	0.67559	1237300	551400	3431300	1882500	1211700	1000800
P05202	Got2	Aspartate aminotransferase, mitochondrial	0.67609	3324500	2071600	18352000	3214900	0	0
A0A494B987;Q3U1J4	Ddb1	DNA damage-binding protein 1	0.68232	1112600	546920	817310	668690	1129200	788510
Q7TMM9	Tubb2a	Tubulin beta-2A chain	0.68469	0	0	9589700	4373800	5197500	6410500
P40142;A0A286YE28	Tkt	Transketolase	0.68559	5202300	2105300	20209000	11645000	14652000	8394400
Q62167;P16381;Q3V086;Q61	Ddx3x;D1Pas1	endent RNA helicase DDX3X;Putative ATP-dependent RNA helicase Pl10	0.6857	2016900	642910	33432000	22327000	40036000	36964000
Q6ZWX6	Eif2s1	Eukaryotic translation initiation factor 2 subunit 1	0.69041	0	0	1858000	1605000	3813000	2493900
H7BWZ3;Q9JM76;D3Z2F7;D3	Arpc3	Actin-related protein 2/3 complex subunit 3	0.70231	973560	455260	0	0	1678700	916170
P38647	Hspa9	Stress-70 protein, mitochondrial	0.71157	29605000	11200000	43685000	29601000	43694000	26465000
P14901	Hmox1	Heme oxygenase 1	0.71532	3968700	2140400	4687200	2721700	2520900	1413200
V9GXV0;A0A668KL51;Q91VA	Idh3b	Isocitrate dehydrogenase [NAD] subunit, mitochondrial	0.71723	390650	260770	2917700	1104900	0	0
Q8R1Q8	Dync1li1	Cytoplasmic dynein 1 light intermediate chain 1	0.71759	1183200	626190	2375500	1874300	1793900	1404300
Q9CWJ9	Atic	protein PURH;Phosphoribosylaminoimidazolecarboxamide formyltransferase;IMP cyclo	0.72115	5269200	1980000	7814100	3761900	1827400	1472500
Q543K9;P23492;A0A2I3BQH2	Pnp;Pnp2	Purine nucleoside phosphorylase	0.7251	0	0	1548900	1104300	2342400	863970
P62305	Snrpe	Small nuclear ribonucleoprotein E	0.72749	412380	244670	1117900	1347300	1796200	1135600
B1AXW5;B1AXW6;P35700;B	Prdx1	Peroxiredoxin-1	0.72897	11724000	4474800	31003000	16389000	81336000	40899000
O35350	Capn1	Calpain-1 catalytic subunit	0.72959	763140	298990	1637800	898080	0	0
H7BX95;Q6PDM2;Q6PDM2-3	Srsf1	Serine/arginine-rich splicing factor 1	0.72969	0	0	1989200	1019700	12402000	1618600
A0A5F8MPB9;P26043;Q7TSC	Rdx	Radixin	0.73156	402580	145500	15501000	7152300	8628300	7604500
M0QWU8;P31786;Q4VWZ5	Dbi	Acyl-CoA-binding protein	0.73303	9307500	2993900	15398000	8591100	0	0
A0A0R4J0Z1;P08003	Pdia4	Protein disulfide-isomerase A4	0.7439	0	0	9894500	5341500	2982800	2146900
P10126;D3Z3I8;D3YZ68;P626	Eef1a1	Elongation factor 1-alpha 1	0.74439	45544000	18538000	100610000	64233000	10234000	7250100

P62814;Q91YH6;A0A0U1RNU	Atp6v1b2	V-type proton ATPase subunit B, brain isoform	0.7453	5177800	2137400	4059500	1685400	0	0
A0A0N4SV66;Q8CGP4;C0HKE	aa;Hist1h2ah;H2afj;Hist1h2ak;Hist1h2af;H	type 1-H;Histone H2A.J;Histone H2A type 1-K;Histone H2A type 1-F;Histone H2A type	0.75093	45764000	16478000	117360000	72568000	144340000	89978000
Q3TWW8	Srsf6	Serine/arginine-rich splicing factor 6	0.75189	0	0	0	0	6726200	5558900
A2AW05;Q08943;Q08943-2	Ssrp1	FACT complex subunit SSRP1	0.75618	0	0	622880	501050	1218800	760560
P20152;A0A0A6YWC8;A2AKJ	Vim	Vimentin	0.75621	538490000	195470000	1035100000	553590000	1009100000	750250000
F8WI35;A0A1W2P768;P8424	H3f3a;Hist1h3b;Hist1h3a;H3f3c	istone H3;Histone H3.3;Histone H3.2;Histone H3.1;Histone H3.3C	0.75629	232680000	79844000	533330000	334640000	278270000	133620000
Q7TPV4	Mybbp1a	Myb-binding protein 1A	0.7573	0	0	425500	380120	1144200	458380
P57784	Snrpa1	U2 small nuclear ribonucleoprotein A	0.75822	759050	443720	1612300	1547800	1608900	775110
P35550;A0A140LIR6;Q80WS	Fbl;Fbll1	transferase fibrillarin;rRNA/tRNA 2-O-methyltransferase fibrillarin-like protein 1	0.75832	0	0	2616000	1852400	2607400	1904300
Q5SXR6;Q68FD5;F6Z1R4	Cltc	Clathrin heavy chain;Clathrin heavy chain 1	0.75928	11002000	4074000	59773000	22829000	10583000	6817400
P42125	Eci1	Enoyl-CoA delta isomerase 1, mitochondrial	0.75974	0	0	2986200	1444200	1486100	1341500
P48962	Slc25a4	ADP/ATP translocase 1	0.76192	43900000	26560000	93559000	47921000	1464700	1665300
A0A0A6YWP6;A0A0A6YX18;	Atp6v1h	V-type proton ATPase subunit H	0.76357	1980100	751540	3489300	1812200	5223100	3691900
A0A140LHC3;A0A140LJH1;A0	Qars		0.76361	369810	202000	917140	614610	2532300	1564800
A0A0N4SVM0;D6RCW7;P477	Capza2	F-actin-capping protein subunit alpha-2	0.76607	2565800	859040	9874000	11719000	6095000	3617500
Q8R1B4;M0QWV3	Eif3c	Eukaryotic translation initiation factor 3 subunit C	0.7664	1200900	581780	2254100	1137400	3646900	2031200
P20065-2;P20065	Tmsb4x	Thymosin beta-4;Hematopoietic system regulatory peptide	0.77177	16088000	6106300	20187000	15639000	1964700	1841600
A0A087WP83;Q8VDJ3;A0A0	Hdlbp	Vigilin	0.77415	2799600	1253100	6773400	4969900	1312800	739310
A0A1B0GSR9;A0A1B0GSX0;	Ldha	L-lactate dehydrogenase A chain	0.77473	2094100	634050	4300500	3256500	5155500	3125800
P62192	Psmc1	26S protease regulatory subunit 4	0.77777	399900	384030	2671700	1643000	1507900	1574000
Q3UMU9-4;Q3UMU9-2;Q3U	Hdgfrp2	Hepatoma-derived growth factor-related protein 2	0.78199	0	0	1693300	1074700	1635600	716580
P26350;A0A087WP98;A0A0	Ptma	osin alpha;Prothymosin alpha, N-terminally processed;Thymosin alpha	0.78572	4078300	1428600	14225000	8649100	9552000	6106400
Q62465	Vat1	Synaptic vesicle membrane protein VAT-1 homolog	0.7868	0	0	3435700	1454900	2086100	2139900
E9Q0U7;Q61699-2;Q61699;	Hsph1	Heat shock protein 105 kDa	0.78761	1766300	794470	3673800	4101600	7927200	4259500
Q8CB58;Q8BGJ5;Q922I7;P17	Ptbp1	Polypyrimidine tract-binding protein 1	0.78926	3819500	1870600	3140900	3155400	8865200	17868000
A6ZI44;P05064;D3Z510;A0A	Aldoa;Aldoart2;Aldoart1	uctose-bisphosphate aldolase;Fructose-bisphosphate aldolase A	0.79094	4134100	1234800	12545000	8920600	15611000	12175000
Q60737	Csnk2a1	Casein kinase II subunit alpha	0.79402	0	0	1131300	1291100	948850	531950
A2A817;A2A816;A2A815;A2	Park7	Protein deglycase DJ-1	0.79425	0	0	5802500	2240800	2166000	1954800
A0A0U1RNQ6;Q8BFR5-2;Q8	Tufm	Elongation factor Tu, mitochondrial	0.79584	1265000	553410	2874900	2819700	2699800	1230900
P29341;Q62029;Q9D4E6;A0	Pabpc1	Polyadenylate-binding protein 1	0.79749	1039500	561690	11185000	7988400	7201100	4438500
E9Q9F5;E9Q1G8;O55131	07-Sep	Septin-7	0.79848	1459600	1428200	4267800	3979100	2949200	2404000
Q9JIW9;P63321	Ralb;Rala	Ras-related protein Ral-B;Ras-related protein Ral-A	0.80408	0	0	850180	741560	1174500	519420
P54227;D3Z5N2;D3Z1Z8	Stmn1	Stathmin	0.80451	2365000	1298700	5240400	2905900	4244300	2416300
P26041	Msn	Moesin	0.8061	21003000	7611200	60380000	101070000	26206000	9755600
P26039;A2AIM2;E9PUM4;A0	Tln1	Talin-1	0.80866	0	0	927180	926560	10114000	3869100
A0A140LIZ5;P54775	Psmc4	26S protease regulatory subunit 6B	0.80916	437500	231840	2431700	965420	1584000	890980
Q05816	Fabp5	Fatty acid-binding protein, epidermal	0.80987	3630300	1951700	20173000	16490000	21220000	13706000
A0A1B0GSG5;Q91VI7;A0A1E	Rnh1	Ribonuclease inhibitor	0.81342	1946500	588170	1112200	1405600	1019400	1117000
P62983;A0A0A6YW67;E9Q9J	Rps27a;Gm8797;Uba52;Kxd1;Ubc;Ubb	S ribosomal protein L40;Ubiquitin;60S ribosomal protein L40;Polyubiquitin-B;Ubiquitin	0.81534	50257000	20577000	69851000	48125000	55049000	33806000
Q9CR51	Atp6v1g1	V-type proton ATPase subunit G 1	0.81726	6432800	2681300	11997000	4588800	7831400	7539100
A2AL12;Q8BG05-2;Q8BG05;	Hnrnpa3	Heterogeneous nuclear ribonucleoprotein A3	0.81917	4744100	2157600	23532000	18092000	6070800	5709100
Q62418-3;Q62418-2;Q62418	Dbnl	Drebrin-like protein	0.81943	639850	536980	1981800	1177900	2148700	1837300
P62806	Hist1h4a	Histone H4	0.82088	256300000	115230000	568650000	416500000	674350000	411570000
P70372	Elavl1	ELAV-like protein 1	0.8212	625350	302430	1550900	1331100	2013600	1234600
P61161	Actr2	Actin-related protein 2	0.82159	1638500	695260	2853100	2220000	1738300	964190

P27612	Plaa	Phospholipase A-2-activating protein	0.82334	475120	261730	1251200	1323700	0	0
P26516	Psmd7	26S proteasome non-ATPase regulatory subunit 7	0.82356	1683000	874880	3885500	2422300	2764900	1686800
B1AU25;Q9Z0X1;Q9Z0X1-2	Aifm1	Apoptosis-inducing factor 1, mitochondrial	0.82396	2049200	870570	1816900	1477100	0	0
Q8BFW7-4;Q8BFW7;Q8BFW	Lpp	Lipoma-preferred partner homolog	0.83129	991930	708230	0	0	3285100	2082100
Q8JZQ9	Eif3b	Eukaryotic translation initiation factor 3 subunit B	0.83283	1034500	927770	2876000	2222300	0	0
Q60972	Rbbp4	Histone-binding protein RBBP4	0.8346	0	0	1602500	854420	1851900	1555200
Q6ZWN5;F7CJS8;D3YWH9;Q	Rps9	40S ribosomal protein S9	0.83536	6094200	2338000	11414000	9562800	2035900	1339900
Q9D8N0	Eef1g	Elongation factor 1-gamma	0.83563	2456800	115620	4405600	2889500	3739200	1143900
D3YVM5;S4R1N1;P14869	Rplp0	60S acidic ribosomal protein P0	0.83577	5203900	2602000	15957000	11777000	16565000	10205000
Q78PY7;Q3TJ56;E9Q3E9	Snd1	Staphylococcal nuclease domain-containing protein 1	0.83584	2502000	1228300	4466600	3200900	3056600	2249500
P97315	Csrp1	Cysteine and glycine-rich protein 1	0.83718	1816400	774890	4594300	2914300	3364900	1824200
Q9QXS1-3;Q9QXS1-10;Q9QX	Plec	Plectin	0.83776	20819000	7510300	55791000	20695000	55622000	26790000
P11499;E9Q0C3;E9PX27;E9Q	Hsp90ab1	Heat shock protein HSP 90-beta	0.84005	43081000	17951000	76931000	49786000	28609000	25768000
A0A0G2JGY8;A0A0G2JEY6;Q	Rpl34	60S ribosomal protein L34	0.84136	1975300	943830	10932000	8403200	11012000	7951000
P63017;Q504P4;D3Z5E2	Hspa8	Heat shock cognate 71 kDa protein	0.84249	43436000	21646000	127270000	103170000	81061000	52186000
Z4YJV4;Q60597-2;Q60597;Q	Ogdh	2-oxoglutarate dehydrogenase, mitochondrial	0.84309	191600	167940	662920	363420	1701200	853760
Q9WTM5;A0A1B0GSR4;A0A	Ruvbl2	RuvB-like 2	0.84383	1371200	965860	961270	955920	1970900	1773500
Q9Z204-4;Q9Z204-3;Q9Z204	Hnrnpc	Heterogeneous nuclear ribonucleoproteins C1/C2	0.84458	7985500	2972600	4508400	3710200	4251100	3673300
A0A3B2WDD2;A0A3B2WBL1	Rpl10a	Ribosomal protein;60S ribosomal protein L10a	0.84814	284200	225420	783880	552270	0	0
Q8BTS0;Q61656;S4R1I6;B1A	Ddx5	Probable ATP-dependent RNA helicase DDX5	0.85111	6483300	3998900	23975000	21029000	18050000	10333000
P58252;G3UXK8;G3UZ34;A2	Eef2	Elongation factor 2	0.85217	24189000	11637000	36242000	27697000	30256000	22253000
Q3U0I3;P80318;E9Q133;F6Q	Cct3	T-complex protein 1 subunit gamma	0.8523	338120	349990	1559700	1537200	2760300	1369500
Q9D8S4;A0A1L1SS58;A0A1L	Rexo2	Oligoribonuclease, mitochondrial	0.8528	569940	298820	2332400	1362600	2105400	1771900
P47911;A0A0J9YU32	Rpl6	60S ribosomal protein L6	0.85304	2103900	1018200	6372800	4616500	8164900	4762400
P62908;A0A140LI77;D3YV43	Rps3	40S ribosomal protein S3	0.85463	1102400	502220	3074600	2023400	2525300	1173400
Q9QUM9;E0CYT2;E0CXB1	Psma6	Proteasome subunit alpha type-6	0.85523	0	0	1262000	697800	6775900	4148500
A0A338P6J9;Q921U7;Q8C28	Cast	Calpastatin	0.85925	664170	490240	3075300	2871700	0	0
P99024;G3UZR1;CON__ENSI	Tubb5	Tubulin beta-5 chain	0.86165	18360000	7414100	95401000	66956000	63233000	63472000
Q8BMS1	Hadha	na, mitochondrial;Long-chain enoyl-CoA hydratase;Long chain 3-hydroxyacyl-CoA dehy	0.8639	0	0	962950	827290	1321300	1012600
P50518;A0A0N4SW34;A0A0I	Atp6v1e1	V-type proton ATPase subunit E 1	0.86608	4675200	2121300	6330900	2366000	7357500	5344300
Q8BP67	Rpl24	60S ribosomal protein L24	0.86644	0	0	10452000	8092300	12077000	6493600
Q6ZWV3;I7HLV2;P86048;A0	Rpl10;Rpl10l	60S ribosomal protein L10;60S ribosomal protein L10-like	0.86734	5892000	2252100	10436000	14903000	4090700	2941900
P60843;Q8BTU6;P10630;P10	Eif4a1;Eif4a2	A-I;Eukaryotic initiation factor 4A-II;Eukaryotic initiation factor 4A-II, N-terminally proc	0.86954	13770000	7557200	14754000	11348000	29275000	15415000
Q63844;A0A0U1RPX4;D3Z3G	Mapk3	togen-activated protein kinase 3;Mitogen-activated protein kinase	0.87114	0	0	1407200	1573800	2120400	1245500
Q9JHJ0;A0A1L1SQ12;A0A1L1	Tmod3	Tropomodulin-3	0.87196	1547000	413880	0	0	1790100	1156300
P62900;A0A0A6YXL3;A0A0A	Rpl31	60S ribosomal protein L31	0.87488	3651300	1972400	7058900	6506700	0	0
P47963	Rpl13	60S ribosomal protein L13	0.87559	6016100	3086600	10757000	9833100	9905800	6731200
O08583-2;Q9JJW6-2;O08583	Alyref;Alyref2	THO complex subunit 4;Aly/REF export factor 2	0.87664	2073600	1144400	3264500	4108700	5058000	2631700
P63028	Tpt1	Translationally-controlled tumor protein	0.87698	4994300	2387200	10830000	6151600	35116000	31735000
F6VQ81;Q3TUJ9;Q8BKP1;Q3	Tpd52l2	Tumor protein D54	0.88016	0	0	1337000	1134000	1428900	1190200
Q9CQM8;O09167	Rpl21	60S ribosomal protein L21	0.8814	3302300	1893200	15606000	14489000	26745000	17235000
B2M1R6;P61979-3;P61979;P	Hnrnpk	Heterogeneous nuclear ribonucleoprotein K	0.88229	2094300	688810	7569000	5961800	2481700	1659100
E9QN08;Q80T06;A0A0R4J1E	Eef1d	Elongation factor 1-delta	0.88404	1335800	7298600	17675000	13320000	19474000	15536000
Q9CQF9;F7CIP8;D3Z275	Pcyox1	Prenylcysteine oxidase	0.88777	0	0	1674000	1587900	1425600	1251900
E9PZF0;Q01768;Q5NC80;P15	Gm20390;Nme2;Nme1	sphate kinase;Nucleoside diphosphate kinase B;Nucleoside diphosphate kinase A	0.8882	24014000	10106000	61218000	39907000	26190000	29752000

Q6ZWQ9;Q3THE2;D3YV37;Q	Myl12a;Myl12b	Myosin regulatory light chain 12B	0.89045	874950	715290	5997200	6679500	5810900	3124600
Q9D8E6	Rpl4	60S ribosomal protein L4	0.89047	4542500	2041300	10347000	10813000	7094800	5003200
A0A0N4SV32;Q3UMP4;Q9CY	Serbp1	Plasminogen activator inhibitor 1 RNA-binding protein	0.89181	12335000	6606800	10742000	7672200	10423000	6312600
Q02819;A0A1B0GR41;A0A1(	Nucb1	Nucleobindin-1	0.8932	0	0	918720	1091500	714470	590440
A0A1B0GR11;Q93092	Taldo1	Transaldolase	0.89344	649490	279870	1647900	2125100	1690700	891150
A0A0A0MQC9;Q9D0R8	Lsm12	Protein LSM12 homolog	0.89413	0	0	1616400	1933900	1861600	905880
O88569-3;O88569-2;O88569	Hnrnpa2b1	Heterogeneous nuclear ribonucleoproteins A2/B1	0.89527	4115500	1439600	18512000	17128000	15414000	9657600
Q61792;A2A6G6;A2A6G7;A2	Lasp1	LIM and SH3 domain protein 1	0.89596	1384400	680530	13322000	14196000	15359000	7681200
Q9CXW4;A2BH06	Rpl11	60S ribosomal protein L11	0.90095	6660200	3412700	21988000	14620000	18543000	8890000
Q5EBP8;P49312;P49312-2	Hnrnpa1	onucleoprotein A1;Heterogeneous nuclear ribonucleoprotein A1, N-terminally process	0.90158	17701000	11376000	37043000	40086000	48899000	25047000
P60710;P63260;E9Q5F4;G3U	Actb;Actg1	smic 1, N-terminally processed;Actin, cytoplasmic 2;Actin, cytoplasmic 2, N-terminally	0.90226	401070000	202130000	822940000	870520000	1094100000	540690000
Q64433;Q9JI95	Hspe1;Cpn10-rs1	10 kDa heat shock protein, mitochondrial	0.90281	0	0	2179500	1609200	16877000	11949000
Q60864	Stip1	Stress-induced-phosphoprotein 1	0.90372	2044100	835550	3166400	2247000	948360	961910
Q62318;Q62318-2	Trim28	Transcription intermediary factor 1-beta	0.90513	1417100	934690	4826100	6697900	6085900	3488500
P35979	Rpl12	60S ribosomal protein L12	0.9059	2149800	1281400	3653200	2758900	30715000	23713000
Q6P069-2;Q6P069	Sri	Sorcin	0.90592	1501900	550360	0	0	1921300	1443300
P12970	Rpl7a	60S ribosomal protein L7a	0.90777	2507400	1199700	11227000	8591400	5807500	3624000
Q9CWK8	Snx2	Sorting nexin-2	0.90861	863230	481670	1819200	2244200	2893600	1594700
P80316;E0CZA1	Cct5	T-complex protein 1 subunit epsilon	0.90957	503910	323040	5449000	4530600	8304500	5875100
Q01853	Vcp	Transitional endoplasmic reticulum ATPase	0.91254	20327000	8977600	37992000	30791000	55917000	45853000
Q62261;A0A0A0MQG2;Q622	Sptbn1	Spectrin beta chain, non-erythrocytic 1	0.91495	1652400	1337800	5819300	6037100	17470000	9199800
Q91VW3;I7HPY0	Sh3bgrl3	SH3 domain-binding glutamic acid-rich-like protein 3	0.91539	13405000	7588100	68336000	53178000	278710	1835400
P51881	Slc25a5	/ATP translocase 2;ADP/ATP translocase 2, N-terminally processed	0.91605	22307000	9875100	34823000	23743000	10958000	9318000
P67984	Rpl22	60S ribosomal protein L22	0.91811	1766600	858110	4431500	3011700	2264500	1867900
Q06185;Q8BTB6	Atp5i;Atp5k	ATP synthase subunit e, mitochondrial	0.9198	3815400	2145900	8625400	7024300	7657800	5775900
Q8BH80;Q9QY76	Vapb	Vesicle-associated membrane protein-associated protein B	0.92358	0	0	2384300	2290800	4831000	3511300
Q3UA95;Q3THW5;P0C0S6;Q	H2afz;H2afv	Histone H2A;Histone H2A.V;Histone H2A.Z	0.92369	307590	227360	1337800	1252300	0	0
P14148;F6XI62	Rpl7	60S ribosomal protein L7	0.92528	610490	371510	9702600	6651100	9197100	6135900
Q61598-2;Q61598;A0A1Y7VI	Gdi2	Rab GDP dissociation inhibitor beta	0.92582	9785100	5048400	33093000	20271000	28706000	19705000
P62751	Rpl23a	60S ribosomal protein L23a	0.92842	11621000	6237100	15403000	14595000	84671000	31978000
P05213;A0A2R8VHF3	Tuba1b	Tubulin alpha-1B chain	0.93121	47008000	21534000	65094000	41092000	14042000	13054000
Q9JHU4;F6ZX84	Dync1h1	Cytoplasmic dynein 1 heavy chain 1	0.93183	4550600	3279300	9622300	4924000	5802200	4444600
A2AFI4;A2AFI3;A0A2I3BRL8;	Rbmx;Rbmxl1	NA-binding motif protein, X chromosome, N-terminally processed;RNA binding motif	0.93206	1272800	938090	5007200	3435400	3476500	2970400
P60867	Rps20	40S ribosomal protein S20	0.93264	2481000	1634000	17760000	15948000	14096000	10085000
P61982	Ywhag	3-3 protein gamma;14-3-3 protein gamma, N-terminally processed	0.93499	1497200	896540	27470000	22209000	29934000	20730000
P47757-2;P47757;P47757-4;	Capzb	F-actin-capping protein subunit beta	0.93585	1504200	1005600	14755000	14724000	26052000	17909000
P43274;I7HFT9;Q07133;P15	Hist1h1e	Histone H1.4	0.93686	14867000	2023900	37974000	11030000	17115000	11128000
P08228	Sod1	Superoxide dismutase [Cu-Zn]	0.93711	9408600	5695300	29423000	28903000	29354000	14928000
E9Q4Q2;D3YZC9;D3YZD0;Q6	Sf1	Splicing factor 1	0.93811	1637500	548390	2201700	2362900	5204500	2920900
A0A1B0GSB2;P19253;A0A1B	Rpl13a	60S ribosomal protein L13a	0.93921	2698300	1394900	7404400	7976500	0	0
P16460	Ass1	Argininosuccinate synthase	0.94043	531910	294280	1658800	394960	1595900	2030800
P13020-2;P13020;A0A0J9YU	Gsn	Gelsolin	0.94184	0	0	2306800	2974300	0	0
Q3UJB0;A0A494B9S9	Sf3b2		0.94509	0	0	2002600	2112000	16627000	481640
P63038;D3Z7J9;D3Z2F2;P63	Hspd1	60 kDa heat shock protein, mitochondrial	0.94766	240780	108870	8109200	6606200	13175000	23608000
Q6ZWZ7;Q9CPR4	Rpl17	60S ribosomal protein L17	0.9482	2906100	1080100	3198500	3023100	2224600	820830

P62082	Rps7	40S ribosomal protein S7		0.9491	1772200	809680	12825000	8615300	10864000	7987200
Q3UGB5;Q9JII5-2;Q9JII5;D3Z	Dazap1	DAZ-associated protein 1		0.94919	1217500	740700	2460400	2164100	1907300	2324800
P20108	Prdx3	Thioredoxin-dependent peroxide reductase, mitochondrial		0.94952	0	0	0	0	10878000	7360900
P14115	Rpl27a	60S ribosomal protein L27a		0.95012	2125700	1357500	9204600	8205700	17417000	11273000
P68510	Ywhah	14-3-3 protein eta		0.95423	755840	470480	2804700	2005500	0	0
P17182;Q6PHC1;B0QZL1;B1A	Eno1	Alpha-enolase;Enolase		0.95505	30094000	14491000	62192000	59905000	70662000	37824000
Q9CZY3;B7ZBY7;E9PY39;Q9C	Ube2v1;Gm20431	Ubiquitin-conjugating enzyme E2 variant 1		0.95749	2224400	1376800	5494400	4062700	6056000	3997600
A2A547;P84099	Rpl19	Ribosomal protein L19;60S ribosomal protein L19		0.95818	10832000	3222600	25419000	18032000	19675000	12763000
A0A1L1SUX8;P01831	Thy1	Thy-1 membrane glycoprotein		0.95874	0	0	2237800	2151000	7092500	4861600
P43276	Hist1h1b	Histone H1.5		0.96097	2526300	1384500	6696800	5564800	4913100	3497400
P62264;D3YVF4;D3Z7I1	Rps14	40S ribosomal protein S14		0.96259	5420200	2700000	11246000	8337100	9619300	6584000
D3YVX4;Q91VZ6	Smap1	Stromal membrane-associated protein 1		0.96355	768730	780750	1911100	1353300	1932800	998820
Q8VDN2;D3YYN7;A0A0G2JG	Atp1a1	Sodium/potassium-transporting ATPase subunit alpha-1		0.966	762160	459520	6224000	7185800	6042600	4282600
Q9D3D9	Atp5d	ATP synthase subunit delta, mitochondrial		0.96663	0	0	4243600	3599000	5574500	3966200
Q9CZN7-2;Q9CZN7	Shmt2	Serine hydroxymethyltransferase		0.96667	1632700	515710	2780500	3342300	0	0
CON__P04264;E9Q0F0			+	0.96947	7141700	2746200	44218000	19954000	13729000	22556000
P20029	Hspa5	78 kDa glucose-regulated protein		0.96954	25718000	12414000	56300000	50911000	54401000	41593000
Q03265;D3Z6F5;D6RJ16	Atp5a1	synthase subunit alpha, mitochondrial;ATP synthase subunit alpha		0.97204	7705900	3677600	49448000	40533000	35041000	24494000
F6YVP7;P62270;S4R1N6;A0A	Gm10260;Rps18	40S ribosomal protein S18		0.97243	2445500	1020700	9071500	7696500	13400000	8070300
P62960;A2BGG7;A0A0A0MC	Ybx1	Nuclease-sensitive element-binding protein 1		0.97257	7910400	4118400	10788000	10375000	1423600	1367600
Q8VEK3-2;Q8VEK3	Hnrnpu	Heterogeneous nuclear ribonucleoprotein U		0.97444	3315800	1411500	12959000	12977000	14362000	7554500
Q6ZWV7	Rpl35	60S ribosomal protein L35		0.97616	6881200	3395100	14545000	11636000	845920	647540
P62702	Rps4x	40S ribosomal protein S4, X isoform		0.97723	4748400	2592800	7729200	6691600	10636000	5966400
Q9CQV8-2;Q9CQV8;A2A5N1	Ywhab	protein beta/alpha;14-3-3 protein beta/alpha, N-terminally processed		0.97764	5253000	2985100	7569700	6857300	5082000	3631200
Q60837	Il12rb1	Interleukin-12 receptor subunit beta-1		0.9788	0	0	278920	288510	1560400	842920
P80314;A0A1W2P7B7;A0A1\	Cct2	T-complex protein 1 subunit beta		0.98209	1547500	749050	4279500	4335700	5019900	3992800
Q60692	Psmb6	Proteasome subunit beta type-6		0.98524	587110	397050	2065100	2191300	3411500	2176100
P14152;A0A5F8MPN8;B1AT(	Mdh1	Malate dehydrogenase, cytoplasmic		0.9854	0	0	700850	517810	2067400	1976300
P08113;F7C312;D3Z1R1	Hsp90b1	Endoplasmin		0.98684	18778000	10733000	27787000	34565000	20288000	23410000
P09411;S4R2M7;P09041	Pgk1;Pgk2	glycerate kinase 1;Phosphoglycerate kinase;Phosphoglycerate kinase 2		0.98826	20034000	9015500	65275000	52603000	67833000	59525000
A0A1B0GQU8;P35980;A0A1I	Rpl18	60S ribosomal protein L18		0.98896	8783800	4738000	13157000	11536000	5726600	3869700
P09405	Ncl	Nucleolin		0.98946	6409500	1839000	20697000	16678000	13333000	8075200
Q99PT1	Arhgdia	Rho GDP-dissociation inhibitor 1		0.994	9258000	6016200	13881000	11319000	10731000	7822200
Q9DB20;F7D3P8;A0A338P77	Atp5o	ATP synthase subunit O, mitochondrial		0.99567	999740	509150	2777700	2575200	2744300	2356300
P0DP28;P0DP27;P0DP26;A0A	Calml3	Calmodulin-like protein 3		0.99757	248570000	134090000	326460000	284500000	98629000	62279000
P47962;D3YYV8	Rpl5	60S ribosomal protein L5		1.0025	11904000	5978300	21065000	16730000	36410000	24642000
G3UVV4;P17710-3;P17710-4	Hk1	Hexokinase;Hexokinase-1		1.003	2992900	1594500	5616700	3515900	2610100	2251300
Q9WUK2-2;Q9WUK2	Eif4h	Eukaryotic translation initiation factor 4H		1.004	3269100	1659800	7498400	6578800	0	0
A0A0G2JES3;A0A140T8T4;P5	Rpl9	60S ribosomal protein L9		1.0077	0	0	1664500	2077700	1903100	2708600
Q9CQR2	Rps21	40S ribosomal protein S21		1.0081	2979000	2108100	6618400	6400000	9684700	6637100
P62267	Rps23	40S ribosomal protein S23		1.0131	3210500	2137700	6036700	5577700	348600	216940
Q8VDD5;A0A2R8W6V7;A0A2	Myh9	Myosin-9		1.0135	27208000	15226000	72087000	90002000	148050000	63468000
A1BN54;Q7TPR4;O88990	Actn1	Alpha-actinin-1		1.0145	2597700	950310	12254000	9808100	11188000	4528200
Q8VIJ6	Sfpq	Splicing factor, proline- and glutamine-rich		1.0176	389920	457270	5321700	4064000	6915400	5533000
P50396;D6RI86;B7FAU8	Gdi1	Rab GDP dissociation inhibitor alpha		1.0189	4573300	1553000	1704000	1330900	0	0

A3KGU7;A3KGU9;P16546;A3	Sptan1	Spectrin alpha chain, non-erythrocytic 1		1.0213	7160900	5089100	19796000	10136000	9393900	7321500
O08749	Dld	Dihydrolipoyl dehydrogenase, mitochondrial		1.0214	829340	610030	1436100	1729700	3567700	2876300
Q80YQ1;P35441	Thbs1	Thrombospondin-1		1.0273	0	0	358250	2375200	13425000	12894000
S4R1W1;A0A0A0MQF6;P168	Gm3839;Gapdh	Glyceraldehyde-3-phosphate dehydrogenase		1.0285	23145000	11946000	49313000	36896000	71338000	45695000
B7FAU9;Q8BTM8;B7FAV1;F(	Flna	Filamin-A		1.0309	5645800	1193000	20656000	7744100	13946000	10006000
P63325;A0A338P731;Q3UW{	Rps10	40S ribosomal protein S10		1.0319	0	0	3445800	3356200	2062600	1766900
P08249;A0A0G2JF23;A0A0G	Mdh2	Malate dehydrogenase, mitochondrial		1.0378	1348200	751750	3834600	3904200	5725400	80384000
P68372;Q9D6F9	Tubb4b;Tubb4a	Tubulin beta-4B chain;Tubulin beta-4A chain		1.0384	2159600	1773300	24503000	28324000	30713000	31184000
P62259;F6WA09;D6REF3	Ywhae	14-3-3 protein epsilon		1.0417	555720	406870	2679500	1983300	0	0
A0A494BA97;Q62422	Ostf1	Osteoclast-stimulating factor 1		1.0431	285080	96422	668310	363620	450850	687900
Q91VD1-2				1.0439	3559700	1742500	5329700	4821400	4328100	4066200
P26443;F7CFA5	Glud1	Glutamate dehydrogenase 1, mitochondrial		1.0449	1245700	738180	4971000	4325400	2207500	1700600
Q9DB05;A0A1B0GR35;P286	Napa	Alpha-soluble NSF attachment protein		1.0476	0	0	2165900	4831000	0	0
P62242	Rps8	40S ribosomal protein S8		1.0479	2281800	1575500	3647400	4113800	5926100	4852000
P14131	Rps16	40S ribosomal protein S16		1.0498	3469400	2671200	8075800	6797400	6565200	5490200
A0A1W2P777;A0A0G2JDL9;(	Rap1b;Rap1a	Ras-related protein Rap-1b;Ras-related protein Rap-1A		1.0512	665990	389950	996830	810780	15561000	13544000
F7BHJ0;Q6IRU5-3;Q6IRU5-2	Cltb	Clathrin light chain B		1.0529	1253000	595130	2323400	2246500	2363300	2885700
A2BE92;A2BE93;Q9EQU5-2;(	Set	Protein SET		1.0573	2643400	1552800	6022400	7260300	5701700	3441000
Q9CPQ1	Cox6c	Cytochrome c oxidase subunit 6C		1.0575	0	0	1893300	1790600	1559100	1448900
A0A1W2P6F6;A0A1W2P7Q9	Myl6	Myosin light polypeptide 6		1.0582	2805800	2210000	10570000	13482000	11879000	14306000
A0A494B969;A0A494BBK1;A	Add3	Gamma-adducin		1.0604	798600	626550	1805300	1095500	1898800	1902400
P34884	Mif	Macrophage migration inhibitory factor		1.0613	3377200	699540	5458400	6560500	3071800	3502400
O88531;B1B0P8;B1B0P9	Ppt1	Palmitoyl-protein thioesterase 1		1.0626	1006700	735080	1718800	1692900	1663300	1095800
A2AVJ7;Q99PL5	Rrbp1	Ribosome-binding protein 1		1.0759	0	0	3525500	3780800	3984800	3364200
P27773;F6Q404	Pdia3	Protein disulfide-isomerase A3		1.0814	40369000	7115100	41025000	40199000	51473000	40840000
P97351	Rps3a	40S ribosomal protein S3a		1.0817	0	0	673190	561400	870150	769440
P57759	Erp29	Endoplasmic reticulum resident protein 29		1.0889	0	0	2213800	2691600	2953200	2596300
Q9DCX2;B1ASE2	Atp5h	ATP synthase subunit d, mitochondrial		1.0895	0	0	4865400	4224800	8625800	5969400
F8WJG3;P62996	Tra2b	Transformer-2 protein homolog beta		1.0905	582330	306020	1212300	2201900	0	0
P97379-2;P97379	G3bp2	Ras GTPase-activating protein-binding protein 2		1.0909	1285100	989570	1949900	2218500	2464800	2044700
P09103;E9Q8G8	P4hb	Protein disulfide-isomerase		1.0944	12260000	7240400	54203000	19286000	33837000	21731000
Q61599	Arhgdib	Rho GDP-dissociation inhibitor 2		1.0948	927190	638860	2226000	1298400	1181400	1988000
P27546;P27546-3;P27546-2;/	Map4	Microtubule-associated protein 4		1.0953	5415400	3601600	8349000	5697700	4615500	4201000
A2ATP6;Q8C854-1;Q8C854-2	Myef2	Myelin expression factor 2		1.0999	957240	479960	1582900	2152000	2899400	3104500
Q9D0J8	Ptms	Parathymosin		1.1001	7896900	2162400	135410000	110060000	100660000	72474000
H7BX22;P34022	Ranbp1	Ran-specific GTPase-activating protein		1.101	1753600	1102000	3928500	4234000	5735100	3751700
P63276	Rps17	40S ribosomal protein S17		1.1018	2723200	1368600	7482900	8708100	13138000	10556000
P12787	Cox5a	Cytochrome c oxidase subunit 5A, mitochondrial		1.1021	1472500	1001200	8396900	6684000	24815000	17185000
A2AAN2;Q8BMA6	Srp68	Signal recognition particle subunit SRP68		1.1082	703410	438880	1424500	1035800	931120	832950
Q9CZU6;Q80X68	Cs;Csl	Citrate synthase, mitochondrial;Citrate synthase		1.1138	1024800	568210	2803700	2701500	3400700	2103500
CON__P34955			+	1.1157	2328700	1693600	5355000	3916900	5059500	4381900
Q3U2G2;Q61316	Hspa4	Heat shock 70 kDa protein 4		1.1218	4229900	1929200	3505500	5082200	2832600	2921500
A0A1L1SQA8;P62852	Rps25	40S ribosomal protein S25		1.122	14075000	10005000	83426000	79628000	67495000	43999000
A0A140LIU3;P56391	Cox6b1	Cytochrome c oxidase subunit 6B1		1.1272	2380800	1651300	0	0	3291200	2160800
Q9DBJ1	Pgam1	Phosphoglycerate mutase 1		1.1316	9032500	5204800	17786000	21171000	106290000	57160000

G3UXT7;Q8CFQ9;P56959;G3	Fus	RNA-binding protein FUS		1.1357	1695000	1422100	5331800	4822400	0	0
P17742;A0A1L1SRX5;A0A1L1	Ppia	-trans isomerase A;Peptidyl-prolyl cis-trans isomerase A, N-terminally processed		1.1358	13457000	6884700	37594000	27444000	127210000	83956000
P24369	Ppib	Peptidyl-prolyl cis-trans isomerase B		1.1397	1676000	1173000	14663000	16305000	3438200	2358500
Q80X90	Flnb	Filamin-B		1.1404	29907000	18967000	53417000	69045000	76516000	49491000
Q5SX49;P62962;CON__P025	Pfn1	Profilin;Profilin-1	+	1.141	3443300	2347300	17720000	19594000	17799000	13933000
P63101;D3YXN6;A0A2I3BQ0	Ywhaz	14-3-3 protein zeta/delta		1.1425	2747400	1691500	2913200	3812300	0	0
A2AVR9;P62627	Dynlrb1	Dynein light chain roadblock-type 1		1.1449	1558900	1105900	2445600	2711700	4620100	2726200
P70349;B0R1E3	Hint1	Histidine triad nucleotide-binding protein 1		1.1472	3981000	2339100	8575900	8296900	13944000	10754000
Q9CQA3	Sdhb	nate dehydrogenase [ubiquinone] iron-sulfur subunit, mitochondrial		1.1484	0	0	1795300	1482300	1443200	1560500
P18760;F8WGL3;A0A494B9A	Cfl1	Cofilin-1		1.153	7890700	4419700	22477000	23672000	34493000	20842000
D3YUT3;D3YUG3;D3Z5R8;D3	Rps19	40S ribosomal protein S19		1.1622	6213500	4809800	15614000	20412000	3250200	2177800
Q8BH64	Ehd2	EH domain-containing protein 2		1.1639	3449200	2663700	7114700	6090600	10515000	18088000
Q80XR6;Q20BD0;Q99020	Hnrnpab	Heterogeneous nuclear ribonucleoprotein A/B		1.164	1790700	1656200	5651300	9227400	7706600	9292200
Q8BKE0;P49722	Psma2	Proteasome subunit alpha type-2		1.165	1017800	701450	2840800	3865100	2058400	1629600
K7N6B7;K9J7H2;K7N6J4;D3Y	1201;Vmn1r121;Gm4214;Vmn1r126;Vmn1	Taste receptor type 2		1.1685	0	0	1031100	1166000	2824100	1506900
P62827	Ran	GTP-binding nuclear protein Ran		1.1689	0	0	2323400	2403900	5729000	3477100
Q6GT24;D3Z0Y2;O08709;Q8	Prdx6	Peroxiredoxin-6		1.1775	0	0	3547700	5344000	0	0
H3BKL5;Q921H8;H3BKA1;H3	Acaa1a;Acaa1b	cyl-CoA thiolase A, peroxisomal;3-ketoacyl-CoA thiolase B, peroxisomal		1.1779	377810	345850	1281300	1894900	2772900	2599400
Q6IRU2;A0A571BEU1	Tpm4	Tropomyosin alpha-4 chain		1.1803	30741000	24250000	41251000	50557000	52205000	31659000
Q61171;D3Z4A4	Prdx2	Peroxiredoxin-2		1.1827	1082600	456280	3809100	2634900	20838000	15004000
H3BLF7;H3BKR2;P62874	Gnb1	uanine nucleotide-binding protein G(I)/G(S)/G(T) subunit beta-1		1.1949	0	0	899980	1940900	1275000	744270
D3Z6P1;D6RCU8;Q9DCL9	Paics	horibosylaminoimidazole-succinocarboxamide synthase;Phosphoribosylaminoimidazol		1.1989	0	0	1491300	1040800	2001900	2994900
P04117	Fabp4	Fatty acid-binding protein, adipocyte		1.1992	2680400	1399400	11925000	15105000	5160500	4392000
Q8BVQ9;P46471	Psmc2	26S protease regulatory subunit 7		1.2009	760440	547610	0	0	4434100	1631100
Q3TJD7;B8JJB3;Q8BVJ7;Q3T	Pdlim7	PDZ and LIM domain protein 7		1.2047	0	0	2957100	3824500	3363100	2474700
A0A2I3BPS1;Q9CQI3;A0A2I3	Gmfb	Glia maturation factor beta		1.208	0	0	456690	568580	635140	795780
Q8BMK4	Ckap4	Cytoskeleton-associated protein 4		1.2107	1400900	1175100	4370800	5238600	12999000	8198600
P62245;F8WJ41;D3YVB4;D3	Rps15a	40S ribosomal protein S15a		1.2142	0	0	6759700	7511400	1373400	994100
Q9CQX2	Cyb5b	Cytochrome b5 type B		1.2213	9538700	5620300	4053300	2551500	13739000	14858000
A2AE89;P10649;F6WHQ7;D3	Gstm1	Glutathione S-transferase Mu 1		1.2245	0	0	936830	1885100	1319100	1039100
P62754	Rps6	40S ribosomal protein S6		1.2256	0	0	5103400	4900300	8211000	7708000
P68254-2;P68254	Ywhaq	14-3-3 protein theta		1.2314	1405400	630310	7211600	3655000	9473500	1421400
Q921R2;P62301;A0A0U1RQ	Rps13	40S ribosomal protein S13		1.2317	1129500	820440	1865900	3054900	0	0
E9Q1K3;F8WHZ9;F8WGR0;C	Add1	Alpha-adducin		1.2332	1252100	729020	1948800	4049400	4577600	4253400
F8WIT2;P14824	Anxa6	Annexin;Annexin A6		1.2358	1042000	455740	1382800	1987500	2411000	9448200
O35639;Q3TET3;A0A0G2JGL	Anxa3	Annexin A3;Annexin		1.2379	447770	1196700	0	0	8281300	4303700
A0A5F8MPK9;Q9EP69	Sacm1l	Phosphatidylinositide phosphatase SAC1		1.2433	1933200	1339800	3544400	4251400	3455700	2911600
Q8R411	Myct1	Myc target protein 1		1.2437	0	0	0	0	2586900	2334800
B0V2N7;P07356;B0V2N5;B0	Anxa2	Annexin;Annexin A2		1.2591	2730700	2431400	14293000	12131000	12943000	9047100
P48722-2;P48722;E0CY23	Hspa4l	Heat shock 70 kDa protein 4L		1.2886	0	0	1721600	1758400	1141500	1296200
CON__P02769			+	1.2928	296490	341940	873810	1089600	1235100	959490
P48678;P48678-2;P48678-3;	Lmna	Prelamin-A/C;Lamin-A/C		1.2939	4438800	2247300	8106400	10831000	9351700	6046800
Q9R0P5	Dstn	Destrin		1.2944	0	0	10780000	14445000	14561000	12374000
Q9WVA4;A0A0A6YXG6;Q9R	Tagln2	Transgelin-2		1.2964	4548300	2481900	25106000	40701000	68021000	32947000
Q9CQ60;F6X8L5;Q8CBG6;D3	Pgls	6-phosphogluconolactonase		1.2979	1418600	1095400	2846500	2574700	2304700	2852300

P43346	Dck	Deoxycytidine kinase		1.3098	19451000	12970000	0	0	48565000	43991000
P14206;A0A1L1SRW0;A0A1L	Rpsa	40S ribosomal protein SA		1.313	4759000	13199000	11892000	15103000	13937000	10877000
O08795;O08795-2	Prkcsh	Glucosidase 2 subunit beta		1.3228	1217400	641790	811710	1905600	0	0
P56480	Atp5b	ATP synthase subunit beta, mitochondrial		1.3444	14485000	6815600	24448000	18119000	19600000	13886000
A0A1L1SV25;P57780;E9Q2W	Actn4	Alpha-actinin-4		1.3498	16581000	11537000	44455000	60350000	83516000	39949000
P56382	Atp5e	ATP synthase subunit epsilon, mitochondrial		1.3558	0	0	1634700	2470400	10135000	4287600
O70400;S4R1V0	Pdlim1	PDZ and LIM domain protein 1		1.3578	2619100	1255500	9872300	43027000	15066000	5089000
D3Z7R6;Q08093	Cnn2	Calponin;Calponin-2		1.3619	1057600	1165700	3529500	4071700	7364300	3072400
Q8VHX6-2;Q8VHX6	Flnc	Filamin-C		1.3635	2120000	1989600	4301600	5002200	6383700	6157500
Q3UHL6;A0A087WR50;A0A0	Fn1	Fibronectin;Anastellin		1.365	22533000	17921000	65953000	180160000	238890000	62264000
G3X973;Q8R4Y4-2;Q8R4Y4;F	Stab1	Stabilin-1		1.3782	689850	461640	3839500	4895400	14863000	5061500
G3UWA1;Q5U465-2;Q5U465	Ccdc125	Coiled-coil domain-containing protein 125		1.3787	0	0	48897000	66881000	65577000	62362000
P07901;B7ZC50	Hsp90aa1	Heat shock protein HSP 90-alpha		1.381	1425000	739050	2310000	4714700	3737800	3035200
Q8BP92	Rcn2	Reticulocalbin-2		1.3873	1067100	1005500	1566100	1698200	2904400	3497000
B1ARA3;B1ARA5;P61255	Rpl26	60S ribosomal protein L26		1.3912	1681900	1302700	6385100	4953600	5908200	7469300
Q8K297	Colgalt1	Procollagen galactosyltransferase 1		1.406	1130800	2083200	1145300	908430	1089700	4388100
A2APB8	Tpx2	Targeting protein for Xklp2		1.4336	1466200	1186700	2670800	11756000	2635900	6666300
E9QP09;S4R1Y1;Q80TT8-4;Q	Cul9	Cullin-9		1.4348	0	0	3673700	5029200	4645200	5077900
P35564	Canx	Calnexin		1.435	0	0	2595700	4176200	2264900	1707000
Q8VCQ8;E9QA16;F6RGN9;F6	Cald1			1.4491	3696600	1988300	7727900	9721700	7326800	4531900
O88325	Naglu			1.4545	432640	551870	2002300	1685700	0	0
Q9CWF2	Tubb2b	Tubulin beta-2B chain		1.4596	0	0	1025000	1762300	3175400	3125800
P48036;A0A0G2JGQ0	Anxa5	Annexin A5		1.4727	32937000	4567200	93840000	27262000	81089000	35323000
F6USD5;F6T4L3;Q80ZP8;Q3T	Manf	Mesencephalic astrocyte-derived neurotrophic factor		1.4758	440390	224840	1147700	1861400	870040	1079400
Q9DCT8;A0A0G2JF37	Crip2	Cysteine-rich protein 2		1.4828	1025300	778860	1727600	3062000	2216900	1828800
P70698	Ctps1	CTP synthase 1		1.4939	335980	343550	447370	1427900	1836700	1216100
P26883;F6X9I3	Fkbp1a	-prolyl cis-trans isomerase FKBP1A;Peptidyl-prolyl cis-trans isomerase		1.5196	5294700	10230000	5223500	7235300	0	0
P21107-2;D3Z2H9;E9Q5J9	Tpm3;Tpm3-rs7	Tropomyosin alpha-3 chain		1.561	37153000	31194000	65480000	96097000	138150000	74394000
Q8K2B3;A0A1Y7VJ55	Sdha	ıate dehydrogenase [ubiquinone] flavoprotein subunit, mitochondrial		1.5943	560950	312230	688500	1187500	1380000	1831800
CON__P12763			+	1.61	2765800	2487800	245280	447180	4445100	5555000
F7DBQ0;Q3TML0;Q922R8	Pdia6	Protein disulfide-isomerase A6		1.6198	872300	694470	2329300	4497200	2856600	2638800
G3X924;Q91VC4	Plvap	Plasmalemma vesicle-associated protein		1.6381	977440	1844300	0	0	4171000	2871400
A0A338P6J5;A0A338P6S7;Q9	Ppil2	Peptidyl-prolyl cis-trans isomerase-like 2		1.6391	0	0	1649700	2403200	1192900	889620
P16045;A0A2R8VHJ0	Lgals1	Galectin-1		1.6602	0	0	0	0	5262700	4607700
E9PWE8;Q3TT92;Q62188	Dpysl3	Dihydropyrimidinase-related protein 3		1.6925	523400	973960	3158700	3323200	3474100	5402100
Q60668-3;Q60668;E9Q5B6;F	Hnrnpd	Heterogeneous nuclear ribonucleoprotein D0		1.7138	1183800	525030	2180600	3479800	1548800	1464600
Q9R0P9	Uchl1	Ubiquitin carboxyl-terminal hydrolase isozyme L1		1.7193	4190500	2258600	1404800	2509100	9903800	5802600
Q9WVK4	Ehd1	EH domain-containing protein 1		1.7544	0	0	1489200	2380700	0	0
E9Q616;A0A494BBD5;G5E8K	Ahnak			1.7751	1754500	1640800	2684300	4327800	0	0
B8JK33;B8JK32;Q9D0E1-2;Q9	Hnrnpm	Heterogeneous nuclear ribonucleoprotein M		1.8003	5771800	3510800	10538000	16099000	5491700	10276000
Q9WTI7;Q9WTI7-4;Q9WTI7-	Myo1c	Unconventional myosin-Ic		1.8579	1668600	1507100	3834100	11601000	6773500	5302700
CON__P13645;A2A513;CON_	Krt10	Keratin, type I cytoskeletal 10	+	1.8772	0	0	1337200	2331700	610330	1389700
P24549;O35945;A0A286YCZ0	Aldh1a1;Aldh1a7	Retinal dehydrogenase 1;Aldehyde dehydrogenase, cytosolic 1		1.8986	96459	1189100	0	0	4374600	5508700
A0A1L1STC6;Q6ZWR6-4;Q6Z	Syne1	Nesprin-1		1.9996	0	0	3622900	6540500	10605000	17163000
CON__P02768-1			+	2.205	0	0	466280	1997700	13179000	10434000

F7A1B4;Q3UAM9;Q63961;F7	Eng	Endoglin	2.2681	0	0	2175500	4865800	0	0
Q8R3C7;Q8BZY3;Q61655	Ddx19b;Ddx19a	ATP-dependent RNA helicase DDX19A	2.2734	1562400	3784000	2892400	6804700	0	0
Q6NSP9;P52927	Hmga2	High mobility group protein HMGI-C	2.3683	0	0	196460	8751100	4878100	1130600
Q9Z0M3	Cdh20	Cadherin-20	2.57	1614600	1682100	3850100	8288000	0	0
H3BKG0;P49817	Cav1	Caveolin-1	2.6385	1580600	3059200	4354700	9647900	0	0
B1AYB7;B1AYB5;B1AYB6	Mbd5	Methyl-CpG-binding domain protein 5	2.6677	5357400	3827500	14351000	29553000	6408600	9687100
Q99N15;A2AFQ2;O08756	Hsd17b10	3-hydroxyacyl-CoA dehydrogenase type-2	2.8122	460530	1487100	1540100	3447100	0	0
P67778;Q5SQG5	Phb	Prohibitin	3.9236	3110100	19673000	9143900	31749000	6123500	23395000
P14069	S100a6	Protein S100-A6	3.9588	2822400	8325600	3620300	15408000	5711000	6247800
P62309	Snrpg	Small nuclear ribonucleoprotein G	4.0688	0	0	675050	1347000	399450	1514900
B1ARB3;Q08481-4;Q08481-3	Pecam1	Platelet endothelial cell adhesion molecule	6.5046	472080	1639400	1028700	4844200	0	0
A0A0A6YWA9;P08752	Gnai2	Guanine nucleotide-binding protein G(i) subunit alpha-2	11.818	0	0	1627700	17310000	891660	11549000
E9Q390;A0A286YDF5;Q69ZN	Myof	Myoferlin	16.125	0	0	2302600	15581000	1886700	1104500
O70435;E0CZ34;F8WH02;E0(	Psma3	Proteasome subunit alpha type-3	20.819	531440	6810500	1725100	21355000	0	0
Q8BH97;A0A1B0GS22;A0A1I	Rcn3	Reticulocalbin-3	21.702	0	1782100	144790	7085400	884890	13919000
Q792F9;Q00651	Itga4	Integrin alpha-4	30.998	3109000	25302000	2033100	46864000	2320500	26802000
Q3V116;E0CXV8;Q7TT35;Q64	Phc1	Polyhomeotic-like protein 1	49.2	188190	5966600	947170	60844000	567780	14699000
A0A087WNK8;P61375;P6300	Lhx1;Lhx5	LIM/homeobox protein Lhx5;LIM/homeobox protein Lhx1	NaN	0	0	0	0	1224900	784860
A0A087WNP6;Q4VAA2-2;Q4	Cdv3	Protein CDV3	NaN	0	0	0	0	0	0
A0A087WNS0;A0A087WQK0	Rpl3	60S ribosomal protein L3	NaN	0	0	0	0	0	1332700
A0A087WQE6;A0A087WNT1	Tceb1	Transcription elongation factor B polypeptide 1	NaN	0	0	0	0	0	0
A0A087WQF8;A0A087WP48	Ktn1	Kinectin	NaN	0	0	0	0	0	0
A0A087WNY2;A0A087WRT0	Atg4b	Cysteine protease ATG4B	NaN	0	0	0	0	0	0
A0A087WNZ7;G5E870;A0A0	Trip12	E3 ubiquitin-protein ligase TRIP12	NaN	0	0	0	0	0	0
A0A087WP00;A0A0A0MQ76	Nop58	Nucleolar protein 58	NaN	0	0	0	0	0	0
E0CY18;A0A087WPF5;Q91X5	Zfand2b	AN1-type zinc finger protein 2B	NaN	0	0	0	0	0	0
A0A087WPL5;E9QNN1;O701	Dhx9	ATP-dependent RNA helicase A	NaN	0	0	0	0	0	0
A0A087WRU0;A0A087WQ94	Tns1		NaN	0	0	0	0	0	0
E9QAN4;G3UW47;Q6TA13;E	Kif1a;Kif1b	sin-like protein;Kinesin-like protein KIF1A;Kinesin-like protein KIF1B	NaN	0	0	0	0	0	0
A0A087WQM1;Q07235;A0A(	Serpine2	Glia-derived nexin	NaN	0	0	722960	457220	0	0
A0A087WQQ3;A0A087WRE9	Rnf2	E3 ubiquitin-protein ligase RING2	NaN	0	0	0	0	0	0
A0A087WR70;A0A087WQS1	Sgk3	Serine/threonine-protein kinase Sgk3	NaN	0	0	0	0	0	0
A0A087WR97;Q921F2	Tardbp	TAR DNA-binding protein 43	NaN	0	0	0	0	0	0
A0A087WRM2;Q91VK4	Itm2c	Integral membrane protein 2C;CT-BRI3	NaN	0	0	0	0	0	0
A0A087WS46;O70251	Eef1b2;Eef1b	Elongation factor 1-beta	NaN	0	0	1049700	826430	0	0
A0A0A0MQA5;P68368;Q9JJZ	Tuba4a;Tuba8	Tubulin alpha-4A chain;Tubulin alpha-8 chain	NaN	0	0	0	0	0	0
A0A0A0MQF5;Q8K2H2	Otud6b	OTU domain-containing protein 6B	NaN	0	0	0	0	0	0
A0A0A0MQM0;P63242;J3QP	Eif5a	translation initiation factor 5A;Eukaryotic translation initiation factor 5A-1	NaN	0	0	967990	603800	0	0
A0A0A6YVU8;Q9JKV1	Gm9774;Adrm1	Proteasomal ubiquitin receptor ADRM1	NaN	0	0	0	0	0	0
B7ZNR9;B2RUE8;A0A0A6YW	Map4k4	Mitogen-activated protein kinase kinase kinase kinase 4	NaN	0	0	0	0	0	0
A0A0A6YX73;Q8K1M3	Prkar2a		NaN	0	0	0	0	0	0
A0A0A6YXG0;Q9JKK1-2;Q9JK	Stx6	Syntaxin-6	NaN	0	0	0	0	0	0
A0A0A6YXS4;A0A0A6YY72;P.	Impdh2	Inosine-5-monophosphate dehydrogenase 2	NaN	0	0	0	0	0	0
A0A0A6YXV0;A0A0R4J107;Q	Apeh	Acylamino-acid-releasing enzyme	NaN	0	0	0	0	0	0
A0A0A6YY39;A2AJ72;Q3TIX6	Fubp3		NaN	0	0	73890	4800400	0	0

A0A0B4J1E2;Q9CSN1	Snw1	SNW domain-containing protein 1	NaN	0	0	0	0	0	0
A0A0B4J1J2	Igkv5-43		NaN	0	0	0	0	0	0
A0A0G2JEF6;A0A0G2JGZ1;A	Lrrfip2		NaN	0	0	0	0	0	0
A0A0H2UH17;A0A0G2JDV6;(	Ubap2l	Ubiquitin-associated protein 2-like	NaN	0	0	0	0	0	0
A0A0G2JE27;Q5RKN9;P4775	Capza1	F-actin-capping protein subunit alpha-1	NaN	0	0	0	0	3596500	2323100
A0A0G2JEC4;Q9JK48-3;Q9JK	Sh3glb1	Endophilin-B1	NaN	0	0	0	0	0	0
A0A0G2JEF4;A0A0G2JFG5;Q	Camkk2	Calcium/calmodulin-dependent protein kinase kinase 2	NaN	0	0	0	0	0	0
A0A0H2UH27;A0A0G2JEP0;C	Fxr1	Fragile X mental retardation syndrome-related protein 1	NaN	0	0	0	0	0	0
A0A0G2JEU1;P47738;D3YYF	Aldh2	Aldehyde dehydrogenase, mitochondrial	NaN	0	0	0	0	0	0
A0A0G2JFX7;Q9CWZ3;A0A0(	Rbm8a	RNA-binding protein 8A	NaN	0	0	0	0	0	0
A0A0G2JG00;Q3TUE1;A0A0(	Fubp1	Far upstream element-binding protein 1	NaN	0	0	684940	4264500	0	0
A0A2R8VHB7;A0A2R8VI28;A	Smarcd3;Smarcd2;Smarcd1	natrix-associated actin-dependent regulator of chromatin subfamily D member 2;SWI	NaN	0	0	0	0	0	0
A0A0J9YUG2;P62141	Ppp1cb	erine/threonine-protein phosphatase PP1-beta catalytic subunit	NaN	0	0	0	0	0	0
A0A0J9YUS5;E9PVC6;E9Q9E	Eif4g1	Eukaryotic translation initiation factor 4 gamma 1	NaN	0	0	0	0	11981000	1270000
A0A0J9YUT8;Q8R3C6	Rbm19	Probable RNA-binding protein 19	NaN	0	0	18949000	1657200	0	0
A0A0J9YVG0;Q61074	Ppm1g	Protein phosphatase 1G	NaN	0	0	0	0	2609000	912800
E9QL12;E9PXU9;A0A0N4SUJ	Dysf	Dysferlin	NaN	0	0	0	0	0	0
A0A0N4SV00;P80313;A0A0N	Cct7	T-complex protein 1 subunit eta	NaN	0	0	0	0	0	0
A0A0N4SV15;Q80UY2-2;Q80	Kcmf1	E3 ubiquitin-protein ligase KCMF1	NaN	0	0	0	0	0	0
A0A0N4SVC2;E9QP00;Q6PFF	Tra2a	Transformer-2 protein homolog alpha	NaN	0	0	0	0	0	0
A2AMI7;Z4YKC4;A0A0R4J112	Eif4g3	Eukaryotic translation initiation factor 4 gamma 3	NaN	0	0	0	0	0	0
A0A0N4SVS6;P53996-2;P539	Cnbp	Cellular nucleic acid-binding protein	NaN	0	0	0	0	0	0
A0A0N4SW65;A0A0N4SWC3	Cmas	N-acylneuraminate cytidylyltransferase	NaN	0	0	0	0	0	0
A0A0N4SW94;O35682	Myadm	Myeloid-associated differentiation marker	NaN	0	0	0	0	0	0
A0A0R3P9C8;Q9DC69	Ndufa9	hydrogenase [ubiquinone] 1 alpha subcomplex subunit 9, mitochondrial	NaN	0	0	0	0	0	0
A0A0R4IZW8;O88456;A0A0F	Capns1	Calpain small subunit 1	NaN	0	0	0	0	0	0
A0A0R4IZW9;Q9DCT5	Sdf2	Stromal cell-derived factor 2	NaN	0	0	0	0	0	0
Q6NS54;A0A0R4IZX2;Q9JHL1	Slc9a3r2	ge regulatory cofactor NHE-RF;Na(+)/H(+) exchange regulatory cofactor NHE-RF2	NaN	0	0	0	0	0	0
A0A0R4IZY9;Q91XI1-2;Q91XI	Dus3l	tRNA-dihydrouridine(47) synthase [NAD(P)(+)]-like	NaN	0	0	0	0	0	0
A0A0R4J047;Q9CYI4;D6RGP6	Luc7l	Putative RNA-binding protein Luc7-like 1	NaN	0	0	1947400	1002300	0	0
A0A0R4J078;Q8VCH8	Ubxn4	UBX domain-containing protein 4	NaN	0	0	0	0	0	0
A0A0R4J079;Q8BMP6	Acbd3	Golgi resident protein GCP60	NaN	0	0	0	0	0	0
A0A0R4J080;Q62130	Ptpn14	Tyrosine-protein phosphatase non-receptor type 14	NaN	0	0	0	0	3254000	0
A0A0R4J083;P51174	Acadl	Long-chain specific acyl-CoA dehydrogenase, mitochondrial	NaN	0	0	956100	269870	0	0
A0A0R4J0A0;F6W2Q5;Q5JC2	Eps15	Epidermal growth factor receptor substrate 15	NaN	0	0	0	0	0	0
A0A0R4J0E4			NaN	0	0	0	0	0	0
A0A0R4J0I4;Q8BIW9	Chtf18	Chromosome transmission fidelity protein 18 homolog	NaN	0	0	11860000	7363600	0	0
A0A0R4J0M9;Q8CDG3	Vcpip1	Deubiquitinating protein VCIP135	NaN	0	0	0	0	0	0
A0A0R4J0Q5;P21619-2;P216	Lmnb2	Lamin-B2	NaN	0	0	0	0	0	0
A0A0R4J0R1;O70404	Vamp8	Vesicle-associated membrane protein 8	NaN	0	0	0	0	0	0
A0A0R4J0S1;Q91W92	Cdc42ep1	Cdc42 effector protein 1	NaN	0	0	0	0	0	0
A0A2I3BQY0;A0A0R4J0T8;Q	Arfgap3	ADP-ribosylation factor GTPase-activating protein 3	NaN	0	0	0	0	0	0
F6TQN9;E9QL31;A0A0R4J10	Dab2	Disabled homolog 2	NaN	0	0	3391100	2568500	0	0
A0A0R4J138;P50429	Arsb	Arylsulfatase B	NaN	0	0	0	0	0	0
A0A0R4J1E3;Q9QXS6-3;Q9Q	Dbn1	Drebrin	NaN	0	0	0	0	0	0

D3Z0Q8;A0A0R4J1H6;E9QP9	Golga3	Golgin subfamily A member 3	NaN	0	0	0	0	0	0
A0A0R4J1L2;F6ZFU0;P57776	Eef1d	Elongation factor 1-delta	NaN	0	0	0	0	0	0
D6RE33;A0A0R4J1Q0;G5E89	Edc4	Enhancer of mRNA-decapping protein 4	NaN	0	0	0	0	0	0
A0A0R4J1W0;B2RXQ9;D3Z0	Sorbs2	Sorbin and SH3 domain-containing protein 2	NaN	0	0	0	0	0	0
A0A0R4J1Y7;E9PXX7;Q91W9	Txndc5	Thioredoxin domain-containing protein 5	NaN	0	0	0	0	0	0
A0A0R4J275;Q7TMF3	Ndufa12	ADH dehydrogenase [ubiquinone] 1 alpha subcomplex subunit 12	NaN	0	0	0	0	0	0
A0A0U1RNJ1;P19096	Fasn	er-protein] synthase;3-oxoacyl-[acyl-carrier-protein] reductase;3-hydroxyacyl-[acyl-car	NaN	0	0	2529900	4710200	0	0
A0A0U1RNT6;A0A0U1RNK6;	Mat2a	S-adenosylmethionine synthase isoform type-2	NaN	4403500	846910	0	0	0	0
A0A0U1RNM2;A1L3S7;Q8VH	Gatad2b	Transcriptional repressor p66-beta	NaN	0	0	0	0	0	0
A0A0U1RP97;P06745;A0A0U	Gpi	Glucose-6-phosphate isomerase	NaN	0	0	0	0	0	0
Q9R166;A0A0U1RPC5	Zfp109		NaN	0	0	0	0	13674000	1024000
D3YZ84;D3YY06;D3YWG5;D6	Tsen34	tRNA-splicing endonuclease subunit Sen34	NaN	0	0	0	0	0	0
A0A140LHW5;A0A140LIK0;A	Spcs2	Signal peptidase complex subunit 2	NaN	0	0	0	0	0	0
A0A140LHU9;A0A140LI54;Q	Pnpla6	Neuropathy target esterase	NaN	0	0	0	0	0	0
D3Z6W1;A0A140LIT9;Q7TSJ	Map6	Microtubule-associated protein 6	NaN	0	0	0	0	0	0
A0A140LJ36;Q9JIG8	Praf2	PRA1 family protein 2	NaN	0	0	0	0	0	0
A0A171EBL2;E9Q555	Rnf213	E3 ubiquitin-protein ligase RNF213	NaN	0	0	0	0	161930000	0
A0A1B0GQZ1;A0A1B0GSZ9;	Mrpl23	39S ribosomal protein L23, mitochondrial	NaN	0	0	0	0	0	0
A0A1B0GR08;Q921G8	Tubgcp2	Gamma-tubulin complex component 2	NaN	0	0	0	0	0	0
A0A1B0GS13;A0A1B0GT81;	Bax	Apoptosis regulator BAX	NaN	0	0	0	0	0	0
Q8K4L2;E9Q3Z5;A0A1B0GS9	Svil	Supervillin	NaN	0	0	0	0	0	0
D3Z0B9;A0A1B0GSU0;Q571	Aldh16a1	Aldehyde dehydrogenase family 16 member A1	NaN	0	0	0	0	0	0
A0A1B0GSX6;O70209	Pdlim3	PDZ and LIM domain protein 3	NaN	0	0	0	0	0	0
A0A1B0GSY1;Q9ESW8	Pgpep1	Pyroglutamyl-peptidase 1	NaN	0	0	0	0	0	0
A0A1B0GX15;G3UWN5;P082	Apoe	Apolipoprotein E	NaN	0	0	0	0	0	0
D6RCH8;F8WHJ1;A0A1C7CYU	Fam160a2	FTS and Hook-interacting protein	NaN	0	0	0	0	0	0
E9QA74;E9Q405;A0A1C7ZN1	Myo18a	Unconventional myosin-XVIIIa	NaN	0	0	0	0	0	0
A0A1D5RM74;A0A1D5RLE4;	Csnk2a2	Casein kinase II subunit alpha	NaN	0	0	0	0	0	0
A0A1D5RLU9;A0A1D5RLE8;A	Cmtm3	CKLF-like MARVEL transmembrane domain-containing protein 3	NaN	0	0	0	0	0	0
A0A1D5RLF2;A0A1D5RLI3;A	Fam192a	Protein FAM192A	NaN	0	0	0	0	0	0
A0A1D5RLS1;Q60902-3;Q60	Eps15l1	Epidermal growth factor receptor substrate 15-like 1	NaN	0	0	0	0	0	0
A0A1D5RLW5;A0A1D5RM85	Rpl18a	60S ribosomal protein L18a	NaN	0	0	2578700	1907900	0	0
A0A1D5RLY6;Q8C052	Map1s	otubule-associated protein 1S;MAP1S heavy chain;MAP1S light chain	NaN	0	0	0	0	0	0
A0A1D5RLZ6;Q9DCC8	Tomm20	Mitochondrial import receptor subunit TOM20 homolog	NaN	0	0	0	0	0	0
A0A1D5RMI0;F8WHV1;Q9D1	Fam96b	Mitotic spindle-associated MMXD complex subunit MIP18	NaN	0	0	0	0	0	0
A0A1L1SR69;A0A1L1ST61;A	Higd1a	HIG1 domain family member 1A, mitochondrial	NaN	0	0	0	0	0	0
A0A1L1SRS6;D3YV69;F7BE3	4;Rab6b;Rab39a;Rab27b;Rab27a;Rasef;C	rted protein Rab-27A;Ras-related protein Rab-34;Ras and EF-hand domain-containing	NaN	0	0	0	0	0	0
A0A1L1SS87;A0A1L1SU40			NaN	0	0	17137000	13594000	0	0
J3QN87;A0A1L1SSA3;Q9CXU	Eif1;Eif1b	c translation initiation factor 1b;Eukaryotic translation initiation factor 1	NaN	0	0	0	0	0	0
A0A1L1SSA8;Q91XE8	Tmem205	Transmembrane protein 205	NaN	0	0	0	0	0	0
Q3UDS7;A0A1L1SSF2;Q8VDL	Adpgk	ADP-dependent glucokinase	NaN	0	0	416520	506060	0	0
A0A1L1STC0;Q61081	Cdc37	o-chaperone Cdc37;Hsp90 co-chaperone Cdc37, N-terminally processed	NaN	0	0	0	0	0	0
Q45VK5;A0A1L1STE4;Q9Z1X	Ilf3	Interleukin enhancer-binding factor 3	NaN	0	0	0	0	0	0
A0A1L1STF0;Q6PDI6-3;Q6PD	Fam63b	Protein FAM63B	NaN	0	0	0	0	0	0
A0A1L1STY4;A0A1L1SVG0;Q	Uaca	Jveal autoantigen with coiled-coil domains and ankyrin repeats	NaN	4246200	0	0	0	0	0

A0A1L1SVK0;Q61206;A0A1L1	Pafah1b2	Platelet-activating factor acetylhydrolase IB subunit beta	NaN	0	0	0	0	0	0
A0A1W2P6H2;A0A1W2P7I2;(	Epb4.1l2;Epb41l2	Band 4.1-like protein 2	NaN	0	0	0	0	0	0
A0A1W2P7I5;A0A1W2P6P1;(	Sgta	ll glutamine-rich tetratricopeptide repeat-containing protein alpha	NaN	0	0	0	0	0	0
A0A1W2P6Y0;Q6P8X1	Snx6	Sorting nexin-6;Sorting nexin-6, N-terminally processed	NaN	0	0	0	0	0	0
A0A1W2P8D6;A0A1W2P727;	Ilvbl	Acetolactate synthase-like protein	NaN	0	0	0	0	0	0
A0A1W2P729;Q9QXT0	Cnpy2	Protein canopy homolog 2	NaN	0	0	0	0	0	0
A0A1W2P733;Q922D8	Mthfd1	se;Methenyltetrahydrofolate cyclohydrolase;Formyltetrahydrofolate synthetase;C-1-te	NaN	0	0	0	0	0	0
A0A1W2P7H2;P62075	Timm13	litochondrial import inner membrane translocase subunit Tim13	NaN	0	0	0	0	0	0
A0A1W2P7Z8;A0A1W2P7H4;	Cdc5l	Cell division cycle 5-like protein	NaN	0	0	0	0	0	0
A0A1W2P7Q6;Q9CRT8	Xpot	Exportin-T	NaN	0	0	0	0	0	0
A0A1W2P7X0;E9QMV2;Q4KN	Abracl	Costars family protein ABRACL	NaN	0	0	0	0	0	0
A0A1W2P7X3;Q9D358;Q561	Acp1	Low molecular weight phosphotyrosine protein phosphatase	NaN	0	0	0	0	0	0
B2RRF0;B1AQN2;A0A1W2P7	Ptprk;Ptprt	eceptor-type tyrosine-protein phosphatase T;Receptor-type tyrosine-protein phosphata	NaN	0	0	2278100	1222400	0	0
A0A1Y7VJ71;P45591	Cfl2	Cofilin-2	NaN	0	0	0	0	2304600	1701900
A0A1Y7VM39;Q3TZZ7	Esyt2	Extended synaptotagmin-2	NaN	0	0	0	0	0	0
A0A1Y7VM80;E9QLA5;Q0GN	Inf2	Inverted formin-2	NaN	0	0	0	0	0	0
F8VPK5;A0A1Y7VMN0;P7033	Rock2	Rho-associated protein kinase;Rho-associated protein kinase 2	NaN	4287600	0	0	0	1450600	1599100
A0A286YDB7;A0A286YCT4;A	Ssr1	Translocon-associated protein subunit alpha	NaN	0	0	0	0	0	0
A0A286YD22;A0A286YDT9;A	Gkap1	G kinase-anchoring protein 1	NaN	0	0	0	0	0	0
A0A286YD68;Q8JZR0	Acsl5	Long-chain-fatty-acid--CoA ligase 5	NaN	0	0	0	0	0	0
A0A286YE75;Q59J78	Ndufaf2	Mimitin, mitochondrial	NaN	0	0	0	0	0	0
A0A2C9F2D2;Q07076;A0A28	Anxa7	Annexin A7	NaN	0	0	0	0	0	0
A0A2I3BR03;A0A2I3BPT1;A0	App	oid protein 40;C83;P3(42);P3(40);C80;Gamma-secretase C-terminal fragment 59;Gam	NaN	0	0	0	0	0	0
A0A2I3BQ39;Q9JKB1;P58321	Uchl3;Uchl4	-terminal hydrolase isozyme L3;Ubiquitin carboxyl-terminal hydrolase isozyme L4	NaN	0	0	1695900	1773900	0	0
A0A2I3BQF4;P62889	Rpl30	60S ribosomal protein L30	NaN	0	0	0	0	0	0
A0A2I3BQJ3;Q8K072	Reep4	Receptor expression-enhancing protein 4	NaN	0	0	0	0	0	0
A0A2I3BQZ0;Q7TN22-2;Q7TI	Txndc16	Thioredoxin domain-containing protein 16	NaN	0	0	0	0	0	0
A0A2R8VI25;Q3TT81;A0A2R8	Pcbp2;Pcbp3	Poly(rC)-binding protein 2;Poly(rC)-binding protein 3	NaN	0	0	2441700	2187700	0	0
A0A2R8VK76;Q9EPU4	Cpsf1	Cleavage and polyadenylation specificity factor subunit 1	NaN	0	0	0	0	0	0
A0A2R8W6Y5;A0A2R8VKL5;I	Larp4	La-related protein 4	NaN	0	0	0	0	0	0
A0A2U3TZ82;Q99KW3-1;Q99	Triobp	TRIO and F-actin-binding protein	NaN	0	0	973380	1996000	0	0
A0A338P675;P47941	Crkl	Crk-like protein	NaN	0	0	0	0	0	0
A0A338P7E5;A0A338P786;P6	Ube2l3	Ubiquitin-conjugating enzyme E2 L3	NaN	0	0	0	0	0	0
A0A338P7D7;Q9Z2U0;B7ZM!	Psma7;Psma8	alpha type-7;Proteasome subunit alpha type;Proteasome subunit alpha type-7-like	NaN	0	0	0	0	0	0
A0A338P7L3;H3BLR8;I1E4X7;	Nudt3	Diphosphoinositol polyphosphate phosphohydrolase 1	NaN	0	0	0	0	0	0
A0A338P7L9;Q9CQ49	Ncbp2	Nuclear cap-binding protein subunit 2	NaN	0	0	0	0	0	0
A0A3B2W7I6;A0A3B2W883;	Srpk1	SRSF protein kinase 1	NaN	0	0	0	0	0	0
A0A3B2WBC6			NaN	0	0	0	0	0	0
A0A3B2WBH9;A0A3B2WCN!	Tjp2	Tight junction protein ZO-2	NaN	0	0	0	0	0	0
A0A3B2WCL5;Q9CT10	Ranbp3	Ran-binding protein 3	NaN	0	0	0	0	0	0
A0A452J8C7;Q8K4Q8	Colec12	Collectin-12	NaN	0	0	0	0	0	0
A0A494B8X7;A0A494B9A2;B	Asah2	Neutral ceramidase;Neutral ceramidase soluble form	NaN	0	0	0	0	0	0
A0A494BA39;A0A494B9F0;A	Nedd4l	E3 ubiquitin-protein ligase NEDD4-like	NaN	0	0	0	0	0	0
G3UXW2;A0A494BA33;A0A4	H2-K1;H2-D1	nain;H-2 class I histocompatibility antigen, K-K alpha chain;H-2 class I histocompatibili	NaN	0	0	0	0	0	0
A0A494BA44;Q04207-2;Q04	Rela	Transcription factor p65	NaN	0	0	0	0	0	0

A0A494BA52			NaN	0	0	0	0	0	0
A0A494BAY0;A0A494BAR3;(	Vps37c	Vacuolar protein sorting-associated protein 37C	NaN	0	0	0	0	0	0
A0A494BB04;Q5FWI3	Tmem2	Transmembrane protein 2	NaN	0	0	0	0	0	0
A0A494BB86;P61164	Actr1a	Alpha-centractin	NaN	0	0	0	0	0	0
A0A494BBM6;R4H4V1;A0A4	Scyl1	N-terminal kinase-like protein	NaN	0	0	0	0	0	0
A0A494BBD8;P10107	Anxa1	Annexin A1	NaN	0	0	0	0	494670	1029700
A0A498WFS2;Q922Y1	Ubxn1	UBX domain-containing protein 1	NaN	0	0	1071800	975180	0	0
G3UZM6;G3UZD6;A0A571BI	Ube4b	Ubiquitin conjugation factor E4 B	NaN	0	0	0	0	0	0
A0A571BGH5;A2A610;A0A57	Gnas	s) subunit alpha isoforms short;Guanine nucleotide-binding protein G(s) subunit alpha	NaN	0	0	0	0	0	0
A0A571BEI2;Q6PIU9		Uncharacterized protein FLJ45252 homolog	NaN	0	0	0	0	0	0
D3YU22;A0A571BG24;Q3UH	Limch1	LIM and calponin homology domains-containing protein 1	NaN	0	0	0	0	0	0
A0A589M675;Q4ACU6-10;Q4	Shank3	SH3 and multiple ankyrin repeat domains protein 3	NaN	0	0	0	0	0	0
A0A5F8MPF6;A0A5F8MPH6;	Arhgef18	Rho guanine nucleotide exchange factor 18	NaN	0	0	0	0	0	0
A0A5F8MPW8;Q3U9D6;Q8R	Exoc6	Exocyst complex component 6	NaN	0	0	0	0	0	0
A0A5K1VVQ1;E9Q8N1;E9Q8	Ttn	Titin	NaN	3759900	652160	927390	0	0	0
A0A668KLV9;A0A668KLD3;Q	Akap12	A-kinase anchor protein 12	NaN	0	0	0	0	0	0
A0A6I8MWW6;Q5SS00	Zdbf2	DBF4-type zinc finger-containing protein 2 homolog	NaN	0	0	0	0	0	0
Q80YW6;Q80YW9;A0A6I8M\	Fkbp15	Peptidyl-prolyl cis-trans isomerase;FK506-binding protein 15	NaN	0	0	0	0	0	0
A0A7N4FLU7;P28028;P2802	Braf	Serine/threonine-protein kinase B-raf	NaN	0	0	0	0	0	0
A0A7N9VR94			NaN	0	0	0	0	0	0
A0A7N9VRC4;Q8R0W0	Eppk1	Epiplakin	NaN	0	0	0	0	0	0
A2A4A6;Q62077	Plcg1	spholipase C;1-phosphatidylinositol 4,5-bisphosphate phosphodiesterase gamma-1	NaN	0	0	0	0	0	0
A2A4H9;Q61576;F6W360	Fkbp10	-prolyl cis-trans isomerase FKBP10;Peptidyl-prolyl cis-trans isomerase	NaN	0	0	0	0	0	0
A6PW84;A2A7Q5;Q3V1T4-2;	P3h1;Lepre1	Prolyl 3-hydroxylase 1	NaN	0	0	0	0	0	0
A2A7S7;Q91WQ3	Yars	ine--tRNA ligase, cytoplasmic;Tyrosine--tRNA ligase, cytoplasmic, N-terminally proce	NaN	0	0	0	0	0	0
A2A863-3;A2A864;A2A863-2	Itgb4	Integrin beta-4;Integrin beta	NaN	0	0	0	0	0	0
A2A9K7	Cnksr1		NaN	0	0	0	0	0	0
A6PWC3;A2A9Q2;Q8BHG1;C	Nrd1	Nardilysin	NaN	0	0	0	0	0	0
A2A9X5;Q9JM14	Nt5c	5(3)-deoxyribonucleotidase, cytosolic type	NaN	0	0	0	0	0	0
A2AAW9;Q9Z0N1	Eif2s3x	Eukaryotic translation initiation factor 2 subunit 3, X-linked	NaN	630070	3825800	0	0	0	0
A2AC13;Q3TLP8;Q05144;P63	Rac3;Rac1;Rac2	bstrate 2;Ras-related C3 botulinum toxin substrate 1;Ras-related C3 botulinum toxin s	NaN	0	0	0	0	0	0
A2AC29;P70245	Ebp	3-beta-hydroxysteroid-Delta(8),Delta(7)-isomerase	NaN	0	0	0	0	3959300	2115000
A2ACG7;Q9DBG6	Rpn2	nyl-diphosphooligosaccharide--protein glycosyltransferase subunit 2	NaN	0	0	0	0	0	0
A2ADF3;A2ADF2;A2ADF1;A2	Fblim1	Filamin-binding LIM protein 1	NaN	0	0	0	0	0	0
A2ADR8;Q8R3G1	Ppp1r8	Nuclear inhibitor of protein phosphatase 1	NaN	0	0	0	0	0	0
A2ADY9	Ddi2	Protein DDI1 homolog 2	NaN	0	0	0	0	0	0
A2AFQ0;Q7TMY8-4;Q7TMY8	Huwe1	E3 ubiquitin-protein ligase HUWE1	NaN	0	0	0	0	0	0
A2AGN7;B7ZCF1;O88685;F6	Psmc3	26S protease regulatory subunit 6A	NaN	0	0	2427500	1306800	0	0
A2AHZ5;Q9ESG4	Tmem27	Collectrin	NaN	0	0	0	0	0	0
A2AJ26;P41234	Abca2	ATP-binding cassette sub-family A member 2	NaN	0	0	0	0	405110	2811500
A2BIN0;B8JI96;L7MUC7;Q58	Mup15;Mup13;Mup10;Mup4;Mup8;Mup9	nary protein 1;Major urinary protein 6;Major urinary protein 17;Major urinary proteins	NaN	0	0	0	702530	0	0
A7TU71;A2ALU4;A2ALU4-2	Shroom2	Protein Shroom2	NaN	0	0	0	0	0	0
A2AM80	Fam43b		NaN	0	0	874320	29571000	0	0
Q6PBC0;A2AMQ5;Q99L43	Cds2	phatidate cytidylyltransferase;Phosphatidate cytidylyltransferase 2	NaN	0	0	0	0	0	0
A2AMY5;Q91VX2;A2AMY7	Ubap2	Ubiquitin-associated protein 2	NaN	0	0	0	0	0	0

Z4YMA7;A2AN08-2;A2AN08-	Ubr4	E3 ubiquitin-protein ligase UBR4		NaN	0	0	0	0	0	0
A2API8;F7AA26;O54931-5;O:	Akap2;Pakap	A-kinase anchor protein 2		NaN	0	0	0	0	0	0
A2AQR0;Q64521	Gpd2	osphate dehydrogenase;Glycerol-3-phosphate dehydrogenase, mitochondrial		NaN	0	0	2709700	2172900	0	0
A2ATI8;A2ATI6;A2ATI9;Q99J:	Gorasp2	Golgi reassembly-stacking protein 2		NaN	0	0	0	0	0	0
Q6PG65;A2ATQ5;P53995	Anapc1	Anaphase-promoting complex subunit 1		NaN	0	0	0	0	0	0
A2AU62;Q64012-2;Q64012	Raly	RNA-binding protein Raly		NaN	0	0	0	0	0	0
A2AWA9	Rabgap1	Rab GTPase-activating protein 1		NaN	0	0	0	0	0	0
A2AWI9;A2AWI7;Q8R3V5-3;	Sh3glb2	Endophilin-B2		NaN	0	0	0	0	0	0
Q3TPJ8;A2BFF9;O88487;A2B	Dync1i2	Cytoplasmic dynein 1 intermediate chain 2		NaN	0	0	0	0	0	0
A2CG44;E9Q3M0;Q8BUN5;C	Smad3;Smad2;Smad9	st decapentaplegic homolog 3;Mothers against decapentaplegic homolog 9;Mothers a		NaN	0	0	0	0	0	0
A3KG36;Q00612	G6pdx	5-phosphate 1-dehydrogenase;Glucose-6-phosphate 1-dehydrogenase X		NaN	0	0	0	0	0	0
E9Q0P6;A3KGA8;P46737-2;P	Brcc3	Lys-63-specific deubiquitinase BRCC36		NaN	0	0	0	0	0	0
A6BLY7	Krt28	Keratin, type I cytoskeletal 28		NaN	0	0	0	0	9968600	2389400
A6H634	Gm266			NaN	0	0	0	0	1144600	0
A6H6E2	Mmrn2	Multimerin-2		NaN	0	0	0	0	0	0
A6X8Z5	Arhgap31	Rho GTPase-activating protein 31		NaN	0	0	0	0	0	0
B0QZL3;Q8BIV7	Slc45a1	Proton-associated sugar transporter A		NaN	0	0	11569000	419610	0	0
B0QZN5;P63044	Vamp2	Vesicle-associated membrane protein 2		NaN	0	0	0	0	0	0
Q3V2Y9;H3BKD1;B1AQF4;Q9	Dusp3	Dual specificity protein phosphatase 3		NaN	0	0	0	0	0	0
B1AQR8;G3X9T7;O08573-3;(	Lgals9	Galectin;Galectin-9		NaN	0	0	0	0	0	0
B7ZC46;B1AQY9;B1AQZ0;Q8	08-Sep	Septin-8		NaN	0	0	0	0	0	0
E9QA63;B1ARU4;E9PVY8;Q9	Macf1	Microtubule-actin cross-linking factor 1		NaN	0	0	0	0	0	0
Q3TRH2;B1AT36;Q9D8W5	Psmd12	26S proteasome non-ATPase regulatory subunit 12		NaN	0	0	0	0	0	0
B1ATL6;P47809	Map2k4	Dual specificity mitogen-activated protein kinase kinase 4		NaN	0	0	0	0	0	0
B1AUD9;Q9DBG9	Tax1bp3	Tax1-binding protein 3		NaN	0	0	0	0	0	0
B1AUX2;Q61191	Hcfc1	-terminal chain 5;HCF N-terminal chain 6;HCF C-terminal chain 1;HCF C-terminal chair		NaN	0	0	0	0	0	0
B1AZ42;P0C0A3	Chmp6	Charged multivesicular body protein 6		NaN	0	0	0	0	0	0
Q8BLJ6;B1AZQ9;B1AZR0	Klhl4			NaN	0	0	0	0	0	0
D3Z5B1;B2RPU8;Q9D1L0	Zbed5;Chchd2	Coiled-coil-helix-coiled-coil-helix domain-containing protein 2		NaN	0	0	0	0	0	0
E9QAI5;G3UWN2;B2RQC6-2	Cad	pendent carbamoyl-phosphate synthase;Aspartate carbamoyltransferase;Dihydroorota		NaN	0	0	0	0	0	0
B2RQS1;Q9ERG2	Strn3	Striatin-3		NaN	0	0	0	0	0	0
S4R2B0;B2RY56	Rbm25	RNA-binding protein 25		NaN	0	0	0	0	0	0
Q9DBQ6;B5B2N5;B5B2N4;Q	Nfatc1	Nuclear factor of activated T-cells, cytoplasmic 1		NaN	0	0	0	0	8774700	995480
D6RFS0;Q3THM8;B7FAU5;I7	Emd	Emerin		NaN	0	0	0	0	0	0
B7ZC18;P42227-2;P42227-3;	Stat3	and activator of transcription;Signal transducer and activator of transcription 3		NaN	0	0	0	0	0	0
B7ZC21;B7ZC22;O70139	Pkig	cAMP-dependent protein kinase inhibitor gamma		NaN	0	0	0	0	0	0
B7ZCM8;B7ZCN0;B7ZCM9;P(	Pla2g4b;Gm28042	Cytosolic phospholipase A2 beta		NaN	0	0	0	0	0	0
V9GXM6;F6R587;B7ZCP4;Q8	Cpne1	Copine-1		NaN	0	0	0	0	0	0
B7ZNL2;Q78ZA7;A0A140LJ37	Nap1l4	Nucleosome assembly protein 1-like 4		NaN	0	0	0	0	0	0
B8JJ90;Q52KR6;B8JJ92;B8JJ9	Acin1	Apoptotic chromatin condensation inducer in the nucleus		NaN	0	0	0	0	0	0
B9EHJ3;P39447;A0A0U1RPW	Tjp1	Tight junction protein ZO-1		NaN	0	0	1690400	2310400	0	0
B9EJR8	Dnaaf5	Dynein assembly factor 5, axonemal		NaN	0	0	0	0	0	0
Q6NZJ5;E9Q3I8;E9Q3I9;E9Q3	Itsn1	Intersectin-1	+	NaN	0	0	19267000	0	0	0
CON__ENSEMBL:ENSBTAP00000007350			+	NaN	0	0	0	0	0	0
CON__ENSEMBL:ENSBTAP00000016046;Q08879;Q08879-2			+	NaN	0	0	0	0	0	0

CON__Q3MHN5;CON__ENSE	Gc	Vitamin D-binding protein	+	NaN	0	0	0	0	0	0
CON__ENSEMBL:ENSBTAP00000024146			+	NaN	0	0	0	0	0	0
CON__P00978			+	NaN	0	0	0	0	0	0
CON__P01966			+	NaN	0	0	0	0	0	0
CON__P02070;CON__Q3SX09			+	NaN	0	0	0	0	0	0
CON__P02533;CON__Q61782			+	NaN	0	0	0	0	0	0
CON__P02666			+	NaN	0	0	0	0	0	0
CON__P35527			+	NaN	0	0	0	0	0	0
CON__P35908;CON__P48668;CON__P04259;CON__P02538;CON__P19013;CON__Q3TTY5;Q3TTY5			+	NaN	0	0	0	0	0	0
CON__Q03247			+	NaN	0	0	0	0	0	0
CON__Q2KJF1			+	NaN	0	0	0	0	0	0
CON__Q2UVX4			+	NaN	0	0	0	0	0	0
CON__Q3T052			+	NaN	0	0	0	0	0	0
CON__Q3ZBS7;P29788			+	NaN	0	0	0	0	0	0
CON__Q6IFZ6;Q6IFZ6	Krt77	Keratin, type II cytoskeletal 1b	+	NaN	0	0	7559700	6885600	0	0
CON__Q7Z794			+	NaN	0	0	0	0	0	0
D3YU17;Q8VCM8	Ncln	Nicalin		NaN	0	0	0	0	0	0
D6RET7;D3YUE7;Q8CB44	Gramd4	GRAM domain-containing protein 4		NaN	0	0	0	0	0	0
D6RCZ7;D6RG51;D3YUH9;Q6	Map4k2	Mitogen-activated protein kinase kinase kinase kinase 2		NaN	0	0	0	0	0	0
D3YV10	Ccdc13	Coiled-coil domain-containing protein 13		NaN	0	0	72261	9620400	0	0
D6RH38;D3Z412;D3YVK9;F7E	Syne2	Nesprin-2		NaN	0	0	815340	997480	0	0
F6RUD1;D3YVW2;Q8BXA1	Golim4	Golgi integral membrane protein 4		NaN	0	0	0	0	0	0
D3YW40;O08915	Aip	AH receptor-interacting protein		NaN	0	0	0	0	0	0
D3Z7K0;D3YWF6;Q7TQI3	Otub1	Ubiquitin thioesterase OTUB1		NaN	0	0	0	0	0	0
F7BX63;F7CV24;D6RHL8;D3Y	Donson	Protein downstream neighbor of Son		NaN	0	0	0	0	0	0
D3YWT1;D3Z3N4	Hnrnph3			NaN	0	0	0	0	0	0
F6YTL8;D3YWX2	Ylpm1			NaN	0	0	0	0	0	0
D3Z4R0;D3YWZ1;F6R8S6;E9	Akt1s1	Proline-rich AKT1 substrate 1		NaN	0	0	0	0	0	0
D3YX34;E9Q586;E9Q3M3;O0	Dctn1	Dynactin subunit 1		NaN	0	0	0	0	0	0
D3YX62;O70252	Hmox2	Heme oxygenase 2		NaN	0	0	292380	6036300	0	0
D3YXF8;J3QQ40;Q3UE61;Q9	Tor1aip1	Torsin-1A-interacting protein 1		NaN	0	0	0	0	0	0
D3YXG2;Q9D997;Q9QZ08	Nagk	N-acetyl-D-glucosamine kinase		NaN	0	0	0	0	0	0
D3YXG6;Q9CVB6	Arpc2	Actin-related protein 2/3 complex subunit 2		NaN	0	0	0	0	0	0
D3YXU1;Q99JY0	Hadhb	ictional enzyme subunit beta, mitochondrial;3-ketoacyl-CoA thiolase		NaN	0	0	0	0	0	0
D3YY48;Q99KJ6;P12265	Gusb	Beta-glucuronidase		NaN	0	0	0	0	0	0
F6UFG6;D3YYE1;D3Z7M9;O3	Anp32a	Acidic leucine-rich nuclear phosphoprotein 32 family member A		NaN	0	0	0	0	0	0
D3YYK8;E9Q6X0;Q8R001-2;C	Mapre2	Microtubule-associated protein RP/EB family member 2		NaN	0	0	0	0	0	0
D3YYM6;D3Z1S8;Q91V55;P9	Rps5	osomal protein S5;40S ribosomal protein S5, N-terminally processed		NaN	0	0	0	0	5015800	5107200
D3YZ06;P14602-2;P14602	Hspb1	Heat shock protein beta-1		NaN	0	0	0	0	0	0
D3YZA1;D3Z7T7;E9PW20;Q9	Chtop	Chromatin target of PRMT1 protein		NaN	0	0	0	0	0	0
D3Z0M2;D3Z191;Q80X71	Tmem106b	Transmembrane protein 106B		NaN	0	0	0	0	0	0
D6RHS6;D3Z1V4;P70296	Pebp1	anolamine-binding protein 1;Hippocampal cholinergic neurostimulating peptide		NaN	0	0	0	0	0	0
E9Q986;D3Z7H6;E9Q907;E9C	Ctnnd1	Catenin delta-1		NaN	0	0	422020	302420	0	0
D3Z5X8;D3Z7N3;D3Z345;Q0	Lyar	Cell growth-regulating nucleolar protein		NaN	0	0	0	0	0	0
D3Z3F1;Q9D824-4;Q9D824-3	Fip1l1	Pre-mRNA 3-end-processing factor FIP1		NaN	0	0	0	0	0	0

D3Z3Q3;Q921U8-2;Q921U8;	Smtn	Smoothelin	NaN	0	0	0	0	0	0
D3Z5B2;Q3UFK8	Frmd8	FERM domain-containing protein 8	NaN	0	0	0	0	0	0
D3Z5G4;O88532	Zfr	Zinc finger RNA-binding protein	NaN	0	0	0	0	0	0
D3Z6C9;Q8BJ48	Nagpa	etylglucosamine-1-phosphodiester alpha-N-acetylglucosaminidase	NaN	0	0	0	0	0	0
D3Z6G3	Mapre3		NaN	0	0	0	0	0	0
D3Z6K5	Mrps10		NaN	0	0	0	0	12015000	0
E9QAT0;D3Z6U8;E9QNF5;Q6	Fmr1	Fragile X mental retardation protein 1 homolog	NaN	0	0	0	0	0	0
D3Z6Z0;Q78ZM0;D3Z789;O7	Snx3	Sorting nexin-3	NaN	0	0	0	0	0	0
D3Z7C0;Q9Z2Q5	Mrpl40	39S ribosomal protein L40, mitochondrial	NaN	0	0	0	0	0	0
G3X922;D4AFX7;A0A1L1STR	Dnajc13		NaN	0	0	0	0	0	0
D6REI7;D6RD00;Q9D7H3	Rtca;RtcA	RNA 3-terminal phosphate cyclase	NaN	0	0	0	0	0	0
D6RFB1;Q9D8C4	Ifi35	Interferon-induced 35 kDa protein homolog	NaN	0	0	0	0	0	0
F7BHM8;D6RFU2;Q99LR1-2;	Abhd12	Monoacylglycerol lipase ABHD12	NaN	0	0	0	0	0	0
D6RH37;P70268;P70268-2	Pkn1	Serine/threonine-protein kinase N1	NaN	0	0	0	0	0	0
D6RI64;Q9R0P4	1110004F10Rik;Smap	Small acidic protein	NaN	0	0	0	0	0	0
D9J2Z9;D9J300;D9J301;D9J3	Pdlim5	PDZ and LIM domain protein 5	NaN	527080	1098400	0	0	0	0
E0CYQ2;Q9CQ48	Nudcd2	NudC domain-containing protein 2	NaN	0	0	0	0	0	0
F6TXE3;F7D432;E9PWE0;E0	Pcmt1	ate O-methyltransferase;Protein-L-isoaspartate(D-aspartate) O-methyltransferase	NaN	0	0	0	0	0	0
E0CZ22	Mroh1		NaN	0	0	0	0	0	0
E9PU87	Sik3		NaN	0	0	0	0	0	0
E9PUB0;Q4LDD4-3;Q4LDD4-	Arap1	with Rho-GAP domain, ANK repeat and PH domain-containing protein 1	NaN	0	0	0	0	0	0
E9PUD2;Q8K1M6-4;Q8K1M6	Dnm1l	Dynamin-1-like protein	NaN	0	0	0	0	0	0
E9PUF7;Q61210-2;Q61210;C	Arhgef1	Rho guanine nucleotide exchange factor 1	NaN	0	0	0	0	0	0
E9PUX0;Q80WJ7	Mtdh	Protein LYRIC	NaN	0	0	0	0	0	0
E9PV22;Q505F5	Lrrc47	Leucine-rich repeat-containing protein 47	NaN	0	0	0	0	0	0
E9PV48;Q64345	I830012O16Rik;Ifit3	Interferon-induced protein with tetratricopeptide repeats 3	NaN	0	0	0	0	0	0
E9PV80;Q61026	Ncoa2	Nuclear receptor coactivator 2	NaN	0	0	0	0	0	0
E9PVA6;Q9JLQ2;F6SLJ2;F6U	Git2	ARF GTPase-activating protein GIT2	NaN	0	0	0	0	0	0
E9PVA8	Gcn1l1		NaN	0	0	0	0	0	0
E9PVM9;E9Q0T0;G5E8W7;F	Ppt2	Lysosomal thioesterase PPT2	NaN	0	0	0	0	0	0
E9PVQ9;E9PVN6;Q9D6K5-2;	Gm20498;Synj2bp	Synaptojanin-2-binding protein	NaN	0	0	0	0	0	0
E9Q175;E9PVU0;E9Q3L1;E9	Myo6	Unconventional myosin-VI	NaN	0	0	0	0	0	0
E9QAH1;E9PVZ8	Golgb1		NaN	0	0	0	0	0	0
E9PW43;Q9CQS8	Gm10320;Sec61b	Protein transport protein Sec61 subunit beta	NaN	0	0	0	0	0	0
E9PWC5;Q9JLB0-2;Q9JLB0	Mpp6	MAGUK p55 subfamily member 6	NaN	0	0	0	0	0	0
F6YTS6;E9PWK1;Q9D379	Ephx1	Epoxide hydrolase 1	NaN	0	0	0	0	0	0
E9PWN3;E9PWN2;Q8BIJ6	Iars2	Isoleucine--tRNA ligase, mitochondrial	NaN	0	0	0	0	0	0
E9PWY9;Q8C0C7;D6RIJ2	Farsa	Phenylalanine--tRNA ligase alpha subunit	NaN	0	0	0	0	0	0
F6V294;E9PX53;E9QPR5;Q8K	Ppp4r1	Serine/threonine-protein phosphatase 4 regulatory subunit 1	NaN	0	0	0	0	0	0
E9PXW9;Q08369	Gata4	Transcription factor GATA-4	NaN	0	0	0	0	0	0
E9PYI8;Q9JMA1	Usp14	n carboxyl-terminal hydrolase;Ubiquitin carboxyl-terminal hydrolase 14	NaN	0	0	0	0	0	0
E9PYX7;E9Q852;E9Q9C3;Q9	Mllt4	Afadin	NaN	0	0	0	0	0	0
E9PZ00;Q8BFQ1;K3W4L3;J3	Psap	Prosaposin	NaN	0	0	0	0	0	0
E9PZ69;P58021	Tm9sf2	Transmembrane 9 superfamily member 2	NaN	0	0	0	0	0	0
E9PZ92;Q3TPX4	Exoc5	Exocyst complex component 5	NaN	0	0	0	0	0	0

E9Q9E4;E9PZX7;P30285;D6R	Cdk4	Cyclin-dependent kinase 4		NaN	0	0	0	0	0	0
E9Q6F4;E9Q039;Q11011;F6V	Npepps	Puromycin-sensitive aminopeptidase		NaN	0	0	0	0	0	0
E9Q3Y1;E9Q6X2;E9Q108;E9C	Serpinb6a;Serpinb6	Serpin B6		NaN	0	0	0	0	0	0
E9Q475;E9Q646;E9Q0Y6;P7C	Ufd1l	Ubiquitin fusion degradation protein 1 homolog		NaN	0	0	0	0	0	0
E9Q1G1;E9Q1H3;G3UY72;G3	Aldh7a1	Alpha-aminoadipic semialdehyde dehydrogenase		NaN	0	0	0	0	0	0
E9Q1J7;Q99MN9;A0A087WC	Pccb	Propionyl-CoA carboxylase beta chain, mitochondrial		NaN	0	0	0	0	0	0
J3QMH1;J3QQ55;E9Q1R7;G3	Tiam1	T-lymphoma invasion and metastasis-inducing protein 1		NaN	0	0	0	0	10233000	570410
E9Q1S3;Q01405	Sec23a	Protein transport protein Sec23A		NaN	0	0	2535400	331760	0	0
F6ZEW4;E9Q1T9;Q9ERK4;E9	Cse1l	Exportin-2		NaN	0	0	0	0	0	0
E9Q242;P54822;E9Q3T7;A0A	Adsl	Adenylosuccinate lyase		NaN	0	0	0	0	0	0
G3UY29;E9Q3P9;F8WGS1;G3	Rab11b;Rab11a	Ras-related protein Rab-11A;Ras-related protein Rab-11B		NaN	0	0	0	0	0	0
E9Q3V6;P42208;F6UKN5;D3Y	02-Sep	Septin-2		NaN	0	0	0	0	0	0
Q8BP43;E9Q450;E9Q452;Q8	Tpm1;Tpm2	Tropomyosin alpha-1 chain;Tropomyosin beta chain	+	NaN	0	0	0	0	5033700	739380
E9Q453;G5E8R2;E9Q456;G5	Tpm1			NaN	0	0	0	0	6632900	2740300
Q6W4W7;E9Q4U7;O70566	Diap2;Diaph2	Protein diaphanous homolog 2		NaN	0	0	0	0	0	0
H3BJG4;E9Q512	Trip11			NaN	0	0	0	0	0	0
E9Q565;Q3UIJ9	Myzap	Myocardial zonula adherens protein		NaN	0	0	0	0	0	0
E9QNH6;E9Q580;Q7TQD7;P4	Myo1b	Unconventional myosin-Ib		NaN	0	0	0	0	0	0
E9Q5B2;G5E8X1;Q9DCS2	0610011F06Rik	UPF0585 protein C16orf13 homolog		NaN	0	0	0	0	0	0
E9Q5G3	Kif23	Kinesin-like protein KIF23		NaN	7182200	0	0	0	0	0
E9Q5L3;Q9DBL1	Acadsb	rt/branched chain specific acyl-CoA dehydrogenase, mitochondrial		NaN	0	0	0	0	0	0
E9Q6J8;Q08274	Dmwd	Dystrophia myotonica WD repeat-containing protein		NaN	0	0	0	0	0	0
E9Q6R3;O08547;A0A0G2JF0	Sec22b	Vesicle-trafficking protein SEC22b		NaN	0	0	0	0	0	0
E9Q6R7;A0A1W2P7C0;Q616	Utrn			NaN	0	0	0	0	0	0
F6T5L3;E9Q717;Q5M8S1;Q9	Atxn3	Ataxin-3		NaN	0	0	0	0	0	0
Q3U3A7;E9Q794;Q3UII8;Q92	Mta3;Mta1	tasis-associated protein MTA3;Metastasis-associated protein MTA1		NaN	0	0	0	0	168970	15344000
E9Q7B0;Q60715-2;Q60715	P4ha1	Prolyl 4-hydroxylase subunit alpha-1		NaN	0	0	0	0	0	0
E9Q7G0;F6ZQA3;A0A1B0GS	Numa1			NaN	0	0	0	0	0	0
E9Q800;Q8CAQ8-3;Q8CAQ8-	Immt	MICOS complex subunit Mic60		NaN	0	0	0	0	0	0
E9Q855;Q3UXS0;O35609	Scamp3	Secretory carrier-associated membrane protein 3		NaN	0	0	0	0	0	0
F7AA45;E9Q8F0;Q8VH51-3;C	Rbm39	RNA-binding protein 39		NaN	0	0	0	0	0	0
E9Q9Q7;E9Q9D1;E9Q9D2;E9	Ablim1	Actin-binding LIM protein 1		NaN	0	0	0	0	0	0
H7BWY4;E9Q9H0;Q811D0-2;	Dlg1	Disks large homolog 1		NaN	0	0	0	0	0	0
E9Q9X4;P70313	Nos3	Nitric oxide synthase;Nitric oxide synthase, endothelial		NaN	0	0	0	0	0	0
E9QAD6;P97450	Atp5j	ATP synthase-coupling factor 6, mitochondrial		NaN	0	0	0	0	0	0
E9QAF9;Q0VGY8	Tanc1	Protein TANC1		NaN	0	0	0	0	0	0
E9QAS4;E9QAS5;Q6PDQ2;F6	Chd4;Chd5	helicase-DNA-binding protein 4;Chromodomain-helicase-DNA-binding protein 5		NaN	0	0	0	0	0	0
E9QAT4;E9QAT4-2;F7BPW6	Sec16a			NaN	0	0	0	0	0	0
E9QMK9;Q8BH86-2;Q8BH86	9030617O03Rik	UPF0317 protein C14orf159 homolog, mitochondrial		NaN	0	0	0	0	0	0
E9QN70;P02469	Lamb1	Laminin subunit beta-1		NaN	0	0	0	0	0	0
G5E8R3;E9QPD7;Q05920	Pcx;Pc	Pyruvate carboxylase;Pyruvate carboxylase, mitochondrial		NaN	0	0	0	0	0	0
E9QPI5;Q6A026;A0A0J9YV33	Pds5a	Sister chromatid cohesion protein PDS5 homolog A		NaN	0	0	0	0	0	0
E9QPX1;P39061-2;P39061-1;	Col18a1	Collagen alpha-1(XVIII) chain;Endostatin		NaN	0	0	0	0	0	0
F6Q8V7;O35129;F6QPR1	Phb2	Prohibitin-2		NaN	0	0	0	0	0	0
F6QA74;P28352	Apex1	apyrimidinic site) lyase;DNA-(apurinic or apyrimidinic site) lyase, mitochondrial		NaN	0	0	0	0	0	0

F6ZML1;F6RXI4	BC067074		NaN	0	0	0	0	0	0
F6SFF5;Q8K1Z0	Coq9	Ubiquinone biosynthesis protein COQ9, mitochondrial	NaN	0	0	0	0	0	0
F6SXM5;P32067	Ssb	Lupus La protein homolog	NaN	0	0	0	0	0	0
F7CUP3;F6UP77;Q8JZV7	Amdhd2	Putative N-acetylglucosamine-6-phosphate deacetylase	NaN	0	0	3081800	455320	0	0
F6XQZ4;Q8BWZ3-2;Q8BWZ3	Naa25	N-alpha-acetyltransferase 25, NatB auxiliary subunit	NaN	0	0	1848900	300060	0	0
F6XVP7;Q924T7-2;Q924T7	Rnf31	E3 ubiquitin-protein ligase RNF31	NaN	0	0	3196100	0	0	0
F6YLI0;Q60865	Caprin1	Caprin-1	NaN	0	0	0	0	0	0
Q7M739;F6ZDS4;F6RX08	Tpr	Nucleoprotein TPR	NaN	504080	813220	0	0	0	0
F7ALS6;P05201	Got1	Aspartate aminotransferase, cytoplasmic	NaN	0	0	0	0	0	0
F7B5B5;Q8VHM5;G3UXU5;S	Hnrnpr;Syncrip	Heterogeneous nuclear ribonucleoprotein Q	NaN	0	0	0	0	0	0
F7CBP1;G3XA17;Q62448-2;C	Eif4g2	Eukaryotic translation initiation factor 4 gamma 2	NaN	0	0	0	0	0	0
F7CUQ1;Q91WX5;O08734	Bak1	Bcl-2 homologous antagonist/killer	NaN	0	0	0	0	0	0
V9GXH3;V9GXF0;V9GXP8;F8	Erc1	ELKS/Rab6-interacting/CAST family member 1	NaN	0	0	0	0	0	0
F8VQJ3;P02468;F6TLW1	Lamc1	Laminin subunit gamma-1	NaN	0	0	0	0	0	0
F8VQN6;Q8R4H2	Arhgef12	Rho guanine nucleotide exchange factor 12	NaN	0	0	0	0	0	0
F8WGE3;Q8K442	Abca8a	ATP-binding cassette sub-family A member 8-A	NaN	0	0	945270	513720	0	0
F8WGG3;Q9JK81;F7A3N3	Myg1	UPF0160 protein MYG1, mitochondrial	NaN	0	0	0	0	0	0
F8WHL2;Q8CIE6;F6XJN3	Copa	Coatomer subunit alpha;Coatomer subunit alpha;Xenin;Proxenin	NaN	0	0	0	0	0	0
F8WHM5;Q61543;F6RSH1	Glg1	Golgi apparatus protein 1	NaN	0	0	0	0	0	0
F8WHR6;Q569Z5-2;Q569Z5;.	Ddx46	Probable ATP-dependent RNA helicase DDX46	NaN	0	0	0	0	0	0
F8WI30	Snx7		NaN	0	0	0	0	0	0
F8WJ30;O70591	Pfdn2	Prefoldin subunit 2	NaN	0	0	0	0	0	0
G3UW70;Q9D1L9	Lamtor5	Ragulator complex protein LAMTOR5	NaN	0	0	0	0	0	0
G3UXA3;G3UWV3;G3V004;C	Calu	Calumenin	NaN	0	0	1874700	1975600	0	0
G3UX48;Q7TSC1	Prrc2a	Protein PRRC2A	NaN	0	0	0	0	0	0
G3UXK7;P53702	Hccs	Cytochrome c-type heme lyase	NaN	0	0	0	0	0	0
G3UXX3;Q91XH5;Q64105;G3	Spr	Sepiapterin reductase	NaN	0	0	1519300	709300	0	0
G3UY42;Q8CCS6-2;Q8CCS6;C	Pabpn1;Gm20521	Polyadenylate-binding protein 2	NaN	0	0	0	0	0	0
G3UY93;Q9Z1Q9;G3UZ22;G3	Vars	Valine--tRNA ligase	NaN	300140	288280	0	0	0	0
G3UYF9;Q03958	Pfdn6	Prefoldin subunit 6	NaN	0	0	4652500	1948800	0	0
G3UYG6;Q6Y7W8;E9Q2M5;C	Gigyf2	PERQ amino acid-rich with GYF domain-containing protein 2	NaN	0	0	0	0	0	0
G3UYG7;Q6P5E4	Uggt1	UDP-glucose:glycoprotein glucosyltransferase 1	NaN	0	0	0	0	0	0
G3UYQ2;G3UZT6;Q3UF95;Q	Bag6	Large proline-rich protein BAG6	NaN	0	0	0	0	0	0
G3UYU4;O08917	Flot1	Flotillin-1	NaN	356460	228680	0	0	0	0
G3UYV7;P62858	Rps28	40S ribosomal protein S28	NaN	0	0	0	0	0	0
G3UYW7;P46938-2;P46938;C	Yap1	Transcriptional coactivator YAP1	NaN	0	0	0	0	0	0
G3UZ21;Q61193	Rgl2	Ral guanine nucleotide dissociation stimulator-like 2	NaN	0	0	0	0	0	0
G3UZJ4;H3BJQ7;P99029-2;P9	Prdx5	Peroxiredoxin-5, mitochondrial	NaN	0	0	0	0	0	0
G3UZP7;P01899;P01897;V9C	2-L;H2-Bl;H2-Q7;H2-Q6;H2-Q4;H2-Q2;H2-C	hain;H-2 class I histocompatibility antigen, Q9 alpha chain;H-2 class I histocompatibil	NaN	0	0	1094900	3777100	0	0
G3X928;Q6NZC7	Sec23ip	SEC23-interacting protein	NaN	0	0	0	0	0	0
G5DDB7;Q8R3B1;Q05DG3	Plcd1	hospholipase C;1-phosphatidylinositol 4,5-bisphosphate phosphodiesterase delta-1	NaN	0	0	0	0	0	0
G5E839;P80315	Cct4	T-complex protein 1 subunit delta	NaN	0	0	0	0	0	0
G5E884;Q8CIN4;Q61036-2;O	Pak1;Pak2;Pak3	ne-protein kinase PAK 2;PAK-2p27;PAK-2p34;Serine/threonine-protein kinase PAK 3;Se	NaN	0	0	0	0	0	0
G5E897;A0A1L1SRB2	Kdelc2		NaN	0	0	436650	1175200	0	0
G5E8E1;A0A087WSF5;A0A08	Lrrfip1	Leucine-rich repeat flightless-interacting protein 1	NaN	0	0	0	0	0	0

G5E8V9;E9QAY5;G5E8V9-2;(	Arfip1		NaN	0	0	0	0	0	0
G5E902;Q8VEM8	Slc25a3	Phosphate carrier protein, mitochondrial	NaN	0	0	0	0	0	0
H3BJ02;I1E4X1;Q8K1E0-2;Q8	Stx5a;Stx5	Syntaxin-5	NaN	0	0	0	0	0	0
H3BKW0;H3BJ30;H3BJW3;Q(	Cpsf6	Cleavage and polyadenylation specificity factor subunit 6	NaN	0	0	0	0	0	0
H3BJU7;H3BJ45;H3BJ40;H3B	Arhgef2	Rho guanine nucleotide exchange factor 2	NaN	0	0	0	0	0	2965000
H3BJ97;Q99JR5	Tinagl1	Tubulointerstitial nephritis antigen-like	NaN	0	0	243600	202360	0	0
H3BJB6;H3BL49;P42932	Cct8	T-complex protein 1 subunit theta	NaN	0	0	0	0	0	0
H3BJZ7;Q4KUS2	Unc13a	Protein unc-13 homolog A	NaN	97460	12678000	0	0	0	0
H3BK43;H3BKH6;Q9R0P3;H3I	Esd	S-formylglutathione hydrolase	NaN	0	0	1650600	1925300	0	0
H3BLH2;Q9R112	Sqrdl	Sulfide:quinone oxidoreductase, mitochondrial	NaN	0	0	0	0	0	0
H7BWY7;Q9Z1K5	Arih1	E3 ubiquitin-protein ligase ARIH1	NaN	0	0	0	0	0	0
H9H9R4;Q9JHJ3;H3BK59;H3B	Glmp	Glycosylated lysosomal membrane protein	NaN	0	0	0	0	0	0
H9KV02;H9KV01;H9KV15;H9I	Son	Protein SON	NaN	0	0	0	0	0	0
J3QMM7;K3W4M4;Q9CZ42-2	Carkd	ATP-dependent (S)-NAD(P)H-hydrate dehydratase	NaN	0	0	0	0	0	0
J3QP56;P97823-2;P97823;J3(	Lypla1;Gm37988	Acyl-protein thioesterase 1	NaN	0	0	0	0	0	0
J3QP71;K3W4Q8;P18572-2;P	Bsg	Basigin	NaN	0	0	0	0	0	0
K3W4T3;Q9Z1G4-3;Q9Z1G4-	Atp6v0a1	oton ATPase subunit a;V-type proton ATPase 116 kDa subunit a isoform 1	NaN	0	0	0	0	0	0
L7N451;Q80SU7;H3BL64	Gvin1	Interferon-induced very large GTPase 1	NaN	0	0	0	0	0	0
M0QWY0;M0QWP2;O70378	Emc8	ER membrane protein complex subunit 8	NaN	0	0	0	0	0	0
M0QWP9;Q9D6J6-2;Q9D6J6	Ndufv2	IADH dehydrogenase [ubiquinone] flavoprotein 2, mitochondrial	NaN	0	0	0	0	0	0
O08529	Capn2	Calpain-2 catalytic subunit	NaN	0	0	0	0	0	0
O08553	Dpysl2	Dihydropyrimidinase-related protein 2	NaN	0	0	0	0	1456700	1685700
O08912	Galnt1	ctosaminyltransferase 1;Polypeptide N-acetylgalactosaminyltransferase 1 soluble for	NaN	0	0	0	0	0	0
O08972;Q99LD8;G3UZR0	Ddah2	N(G),N(G)-dimethylarginine dimethylaminohydrolase 2	NaN	0	0	0	0	1122700	563370
Q3TMX0;O08992;A2AKJ9;A2/	Sdcbp	Syntenin-1	NaN	0	0	1566400	1297500	0	0
O09005	Degs1	Sphingolipid delta(4)-desaturase DES1	NaN	0	0	0	0	4636800	1520700
O09053	Wrn	Werner syndrome ATP-dependent helicase homolog	NaN	0	0	0	0	0	7481100
O09159	Man2b1	Lysosomal alpha-mannosidase	NaN	0	0	1460900	1118000	0	0
O35127	Grcc10	Protein C10	NaN	0	0	0	0	0	0
O35295	Purb	Transcriptional activator protein Pur-beta	NaN	1431100	3449900	0	0	0	0
Q8C391;Q9CXE1;O35382	Exoc4	Exocyst complex component 4	NaN	0	0	0	0	0	0
O35465;O35465-2;F6WP10	Fkbp8	Peptidyl-prolyl cis-trans isomerase FKBP8	NaN	0	0	0	0	0	0
O35598	Adam10	Disintegrin and metalloproteinase domain-containing protein 10	NaN	0	0	0	0	0	0
O35608	Angpt2	Angiopoietin-2	NaN	0	0	0	0	0	0
O35704	Sptlc1	Serine palmitoyltransferase 1	NaN	0	0	0	0	0	0
Q8C2Q7;O35737	Hnrnph1	ibonucleoprotein H;Heterogeneous nuclear ribonucleoprotein H, N-terminally processe	NaN	0	0	0	0	0	0
O35857	Timm44	Mitochondrial import inner membrane translocase subunit TIM44	NaN	0	0	0	0	0	0
O35901;O35900	Lsm2	U6 snRNA-associated Sm-like protein LSm2	NaN	0	0	0	0	0	0
O54724	Ptrf	Polymerase I and transcript release factor	NaN	0	0	0	0	0	0
O54734	Ddost	-diphosphooligosaccharide--protein glycosyltransferase 48 kDa subunit	NaN	0	0	0	0	0	0
O54782;F6TMZ3	Man2b2	Epididymis-specific alpha-mannosidase	NaN	0	0	1439200	697550	0	0
O54941	Smarce1	natrix-associated actin-dependent regulator of chromatin subfamily E member 1	NaN	0	0	0	0	0	0
O54988-2;O54988	Slk	STE20-like serine/threonine-protein kinase	NaN	0	0	0	0	0	0
O55135;A6PWZ2	Eif6	Eukaryotic translation initiation factor 6	NaN	0	0	0	0	0	0
O55201-2;O55201	Supt5h	Transcription elongation factor SPT5	NaN	0	0	0	0	0	0

Q8BTY5;O55234	Psmb5	Proteasome subunit beta type-5	NaN	0	0	0	0	0	0
O70194	Eif3d	Eukaryotic translation initiation factor 3 subunit D	NaN	0	0	675440	275220	0	0
Q8BH40;O70439	Stx7	Syntaxin-7	NaN	0	0	0	0	0	0
O70475;D3YXP9	Ugdh	UDP-glucose 6-dehydrogenase	NaN	0	0	0	0	0	0
O88342;A0A0J9YU05	Wdr1	WD repeat-containing protein 1	NaN	0	0	0	0	0	0
Q5D0E0;O88351	Ikbkb	Inhibitor of nuclear factor kappa-B kinase subunit beta	NaN	0	0	0	0	0	0
Q91XH6;O88384;F6UHS3;E0(	Vti1b	'esicle transport through interaction with t-SNAREs homolog 1B	NaN	0	0	0	0	0	0
O88544;F6QTS1;D3Z1R9;D3Y	Cops4	COP9 signalosome complex subunit 4	NaN	286850	346310	0	0	0	0
O88587-2;O88587	Comt	Catechol O-methyltransferase	NaN	0	0	940330	512620	0	0
O88630	Gosr1	Golgi SNAP receptor complex member 1	NaN	0	0	0	0	0	0
Q3UDC3;O88746;A0A1D5RM	Tom1	Target of Myb protein 1	NaN	0	0	0	0	0	0
O88844;A0A087WRM4;D3YV	Idh1	Isocitrate dehydrogenase [NADP] cytoplasmic	NaN	0	0	1844300	1721900	0	0
O89017	Lgmn	Legumain	NaN	0	0	0	0	0	0
O89023	Tpp1	Tripeptidyl-peptidase 1	NaN	0	0	0	0	0	0
O89086;Q8BG13;S4R2M6	Rbm3	RNA-binding protein 3	NaN	0	0	0	0	0	0
P00520-2;P00520-3;P00520;I	Abl1	Tyrosine-protein kinase ABL1	NaN	0	0	0	0	0	0
P01101	Fos	Proto-oncogene c-Fos	NaN	0	0	4689200	14255000	0	0
P01790;P01789;P01794;P017	Ighv7-1	Ig heavy chain V region HPCG8;Ig heavy chain V region HPCM6;Ig heavy chain V region	NaN	0	0	0	0	0	0
P01803		Ig heavy chain V region AMPC1	NaN	0	0	0	0	3336700	138130
P01887	B2m	Beta-2-microglobulin	NaN	0	0	0	0	0	0
P03930	Mtatp8	ATP synthase protein 8	NaN	0	0	0	0	0	0
P03958	Ada	Adenosine deaminase	NaN	4061300	1539900	0	0	0	0
P05063	Aldoc	Fructose-bisphosphate aldolase C	NaN	0	0	0	0	0	0
P06797	Ctsl	Cathepsin L1;Cathepsin L1 heavy chain;Cathepsin L1 light chain	NaN	0	0	0	0	0	0
Q3TQP6;P06801	Me1	Malic enzyme;NADP-dependent malic enzyme	NaN	0	0	0	0	0	0
P08030;A0A1D5RLR6	Aprt	Adenine phosphoribosyltransferase	NaN	0	0	0	0	0	0
P08122	Col4a2	Collagen alpha-2(IV) chain;Canstatin	NaN	0	0	0	0	0	0
P09055;P09055-2	Itgb1	Integrin beta-1	NaN	0	0	0	0	0	0
P10493	Nid1	Nidogen-1	NaN	0	0	0	0	0	0
P10605	Ctsb	Cathepsin B;Cathepsin B light chain;Cathepsin B heavy chain	NaN	0	0	0	0	0	0
Q8CBB6;Q8CGP2;Q8CGP1;Q(	Hist2h2bb;Hist1h2bh;Hist1h2bb;Hist1h2bm	2-B;Histone H2B type 1-H;Histone H2B type 1-B;Histone H2B type 1-M;Histone H2B t	NaN	0	0	0	0	0	0
P11438	Lamp1	Lysosome-associated membrane glycoprotein 1	NaN	0	0	0	0	0	0
P11688	Itga5	rin alpha-5;Integrin alpha-5 heavy chain;Integrin alpha-5 light chain	NaN	0	0	0	0	0	0
P11881-8;P11881-7;P11881-(	Itpr1	Inositol 1,4,5-trisphosphate receptor type 1	NaN	0	0	0	0	0	0
P11983;P11983-2;F2Z483	Tcp1	T-complex protein 1 subunit alpha	NaN	0	0	0	0	0	0
P13597-2;P13597	Icam1	Intercellular adhesion molecule 1	NaN	0	0	0	0	0	0
P14211	Calr	Calreticulin	NaN	0	0	0	0	0	0
P14685;F7B7L8	Psmd3	26S proteasome non-ATPase regulatory subunit 3	NaN	0	0	0	0	0	0
P14873	Map1b	ıbule-associated protein 1B;MAP1B heavy chain;MAP1 light chain LC1	NaN	0	0	0	0	0	0
Q3USD9;P15975-2;P15975	Usp53	Inactive ubiquitin carboxyl-terminal hydrolase 53	NaN	0	0	468520	541430	0	0
P17427;A0A140LHG0;P1742(	Ap2a2	AP-2 complex subunit alpha-2	NaN	0	0	0	0	0	0
Q61696;P17879;P16627	Hspa1a;Hspa1b;Hspa1l	kDa protein 1A;Heat shock 70 kDa protein 1B;Heat shock 70 kDa protein 1-like	NaN	0	0	0	0	0	0
P18406	Cyr61	Protein CYR61	NaN	0	0	0	0	1487900	740990
P19324;A0A140LHR4;A0A14(	Serpinh1	Serpin H1	NaN	0	0	0	0	0	0
P19783;M0QWX7	Cox4i1	Cytochrome c oxidase subunit 4 isoform 1, mitochondrial	NaN	0	0	0	0	3835400	450630

P19788	Mgp	Matrix Gla protein	NaN	0	0	0	0	0	0
P21278;A0A494BBL5;P30677	Gna11;Gna14	nding protein subunit alpha-11;Guanine nucleotide-binding protein subunit alpha-14	NaN	0	0	0	0	0	0
P21279	Gnaq	Guanine nucleotide-binding protein G(q) subunit alpha	NaN	0	0	0	0	0	0
P21956-2;P21956	Mfge8	Lactadherin	NaN	0	0	0	0	0	0
P23116	Eif3a	Eukaryotic translation initiation factor 3 subunit A	NaN	0	0	0	0	0	0
P23780	Glb1	Beta-galactosidase	NaN	0	0	0	0	0	0
P24270;A2AL20	Cat	Catalase	NaN	0	0	0	0	0	0
P24668	M6pr	Cation-dependent mannose-6-phosphate receptor	NaN	0	0	0	0	0	0
P27046	Man2a1	Alpha-mannosidase 2	NaN	0	0	0	0	0	0
P28271	Aco1	Cytoplasmic aconitate hydratase	NaN	0	0	0	0	0	0
P28651	Ca8	Carbonic anhydrase-related protein	NaN	0	0	0	0	0	0
Q3TF41;Q8BSH9;P28656	Nap1l1	Nucleosome assembly protein 1-like 1	NaN	0	0	0	0	0	0
Q3U9N4;P28798;H3BJE0	Grn	nin;Granulin-1;Granulin-2;Granulin-3;Granulin-4;Granulin-5;Granulin-6;Granulin-7	NaN	0	0	0	0	0	0
Q68FM4;P28828;A0A286YDL	Ptprm	tyrosine-phosphatase;Receptor-type tyrosine-protein phosphatase mu	NaN	0	0	0	0	4266900	218230
Q9CPX4;P29391;A0A1B0GR6	Ftl1;Ftl2	Ferritin;Ferritin light chain 1;Ferritin light chain 2	NaN	0	0	0	0	0	0
P29416	Hexa	Beta-hexosaminidase subunit alpha	NaN	0	0	0	0	0	0
P30412	Ppic	Peptidyl-prolyl cis-trans isomerase C	NaN	0	0	0	0	0	0
P30416	Fkbp4	s isomerase FKBP4;Peptidyl-prolyl cis-trans isomerase FKBP4, N-terminally processed	NaN	0	0	0	0	0	0
P31001	Des	Desmin	NaN	0	0	0	0	0	0
P31324	Prkar2b	cAMP-dependent protein kinase type II-beta regulatory subunit	NaN	0	0	0	0	0	0
P32507-2;P32507	Pvrl2	Nectin-2	NaN	0	0	0	0	0	0
P32921-2;P32921	Wars	Tryptophan--tRNA ligase, cytoplasmic;T1-TrpRS;T2-TrpRS	NaN	0	0	0	0	0	0
P35235;P35235-1	Ptpn11	Tyrosine-protein phosphatase non-receptor type 11	NaN	0	0	0	0	0	0
Q8C266;P35278	Rab5c	Ras-related protein Rab-5C	NaN	0	0	0	0	0	0
P35282;A0A1W2P6Z4	Rab21	Ras-related protein Rab-21	NaN	0	0	0	0	0	0
P35456	Plaur	Urokinase plasminogen activator surface receptor	NaN	0	0	0	0	0	0
P35486	Pdha1	ehydrogenase E1 component subunit alpha, somatic form, mitochondrial	NaN	0	0	0	0	0	0
Q05DV1;P37040;F6R7H8;E9F	Por	NADPH--cytochrome P450 reductase	NaN	0	0	0	0	0	0
P37889-2;P37889	Fbln2	Fibulin-2	NaN	0	0	0	0	0	0
P40336;P40336-2	Vps26a	Vacuolar protein sorting-associated protein 26A	NaN	0	0	0	0	0	0
P42669	Pura	Transcriptional activator protein Pur-alpha	NaN	0	0	0	0	0	0
P46061;A0A2R8W753;A0A2F	Rangap1	Ran GTPase-activating protein 1	NaN	0	0	0	0	0	0
P46414	Cdkn1b	Cyclin-dependent kinase inhibitor 1B	NaN	0	0	0	0	0	0
P46467	Vps4b	Vacuolar protein sorting-associated protein 4B	NaN	0	0	0	0	0	0
P46935;A0A571BEP7;A0A57	Nedd4	E3 ubiquitin-protein ligase NEDD4	NaN	0	0	0	0	0	0
P48437	Prox1	Prospero homeobox protein 1	NaN	0	0	0	0	0	0
P49070	Camlg	Calcium signal-modulating cyclophilin ligand	NaN	0	0	0	0	0	0
P49717	Mcm4	DNA replication licensing factor MCM4	NaN	0	0	0	0	0	0
P50427	Sts	Steryl-sulfatase	NaN	0	0	0	0	0	0
P50580-2;P50580	Pa2g4	Proliferation-associated protein 2G4	NaN	0	0	0	0	0	0
P50636	Rnf19a	E3 ubiquitin-protein ligase RNF19A	NaN	0	0	0	0	0	0
P51480;P51480-2	Cdkn2a	Cyclin-dependent kinase inhibitor 2A	NaN	0	0	0	0	0	0
Q8BGZ6;P51569	Gla	Alpha-galactosidase A	NaN	0	0	0	0	0	0
P51660	Hsd17b4	nctional enzyme type 2;(3R)-hydroxyacyl-CoA dehydrogenase;Enoyl-CoA hydratase 2	NaN	0	0	0	0	0	0
P52293;A2A600;A2A601;A6P	Kpna2	Importin subunit alpha-1	NaN	0	0	0	0	0	0

P52479;P52479-2	Usp10	Ubiquitin carboxyl-terminal hydrolase 10	NaN	0	0	0	0	0	0
P52825;A2A8E7;A2A8E8	Cpt2	Carnitine O-palmitoyltransferase 2, mitochondrial	NaN	0	0	0	0	0	0
P52875	Tmem165	Transmembrane protein 165	NaN	0	0	0	0	0	0
P53986	Slc16a1	Monocarboxylate transporter 1	NaN	0	0	0	0	0	0
P53994;A0A1D5RMH1;S4R23	Rab2a;Rab4b;Rab2b	Ras-related protein Rab-2A;Ras-related protein Rab-2B	NaN	0	0	0	0	0	0
P54116	Stom	Erythrocyte band 7 integral membrane protein	NaN	0	0	0	0	0	0
P54728	Rad23b	UV excision repair protein RAD23 homolog B	NaN	0	0	0	0	0	0
P54823	Ddx6	Probable ATP-dependent RNA helicase DDX6	NaN	0	0	0	0	0	0
P55284	Cdh5	Cadherin-5	NaN	0	0	0	0	0	0
P55302	Lrpap1	Alpha-2-macroglobulin receptor-associated protein	NaN	0	0	0	0	0	0
P56389	Cda	Cytidine deaminase	NaN	0	0	0	0	0	0
Q3U4W8;P56399;D3YYA5;D3	Usp5	n carboxyl-terminal hydrolase;Ubiquitin carboxyl-terminal hydrolase 5	NaN	0	0	0	0	0	0
P56812;D3Z7Q5	Pdcd5	Programmed cell death protein 5	NaN	0	0	0	0	0	0
P57716	Ncstn	Nicastrin	NaN	0	0	0	0	0	0
V9GXI9;P58404-2;P58404	Strn4	Striatin-4	NaN	0	0	0	0	0	0
P58871;Z4YJL4;A0A571BEG9	Tnks1bp1	182 kDa tankyrase-1-binding protein	NaN	0	0	0	0	0	0
P58929	Gmeb2	Glucocorticoid modulatory element-binding protein 2	NaN	1335300	22077000	0	0	0	0
P59999;Q3TX55;E9PWA7	Arpc4	Actin-related protein 2/3 complex subunit 4	NaN	0	0	0	0	0	0
P60122	Ruvbl1	RuvB-like 1	NaN	0	0	0	0	0	0
P60670-2;P60670	Nploc4	Nuclear protein localization protein 4 homolog	NaN	0	0	0	0	0	0
P61089	Ube2n	Ubiquitin-conjugating enzyme E2 N	NaN	0	0	0	0	0	0
P61804	Dad1	l-diphosphooligosaccharide--protein glycosyltransferase subunit DAD1	NaN	0	0	0	0	0	0
P61967	Ap1s1	AP-1 complex subunit sigma-1A	NaN	0	0	0	0	0	0
P62071;A0A1B0GRG1;A0A1E	Rras2	Ras-related protein R-Ras2	NaN	0	0	0	0	0	0
P62077	Timm8b	itochondrial import inner membrane translocase subunit Tim8 B	NaN	467120	246010	0	0	0	0
P62137	Ppp1ca	erine/threonine-protein phosphatase PP1-alpha catalytic subunit	NaN	0	0	0	0	0	0
Q8K1K2;P62196	Psmc5	26S protease regulatory subunit 8	NaN	0	0	0	0	0	0
P62307	Snrpf	Small nuclear ribonucleoprotein F	NaN	0	0	0	0	0	0
P62311	Lsm3	U6 snRNA-associated Sm-like protein LSm3	NaN	0	0	0	0	0	0
P62317	Snrpd2	Small nuclear ribonucleoprotein Sm D2	NaN	0	0	0	0	0	0
P62320	Snrpd3	Small nuclear ribonucleoprotein Sm D3	NaN	0	0	5681200	10919000	0	0
P62334	Psmc6	26S protease regulatory subunit 10B	NaN	0	0	0	0	0	0
P62715;P63330;P97470	Ppp2cb;Ppp2ca	catalytic subunit beta isoform;Serine/threonine-protein phosphatase 2A catalytic sub	NaN	0	0	0	0	0	0
P62869	Tceb2	Transcription elongation factor B polypeptide 2	NaN	0	0	0	0	0	0
P63024	Vamp3	Vesicle-associated membrane protein 3	NaN	0	0	0	0	0	0
P63037;B1AXY0;B1AXY1	Dnaja1	DnaJ homolog subfamily A member 1	NaN	0	0	0	0	0	0
P63213	Gng2	anine nucleotide-binding protein G(I)/G(S)/G(O) subunit gamma-2	NaN	0	0	0	0	0	0
P68040	Gnb2l1	ubunit beta-2-like 1;Guanine nucleotide-binding protein subunit beta-2-like 1, N-termi	NaN	0	0	0	0	0	0
P68369;P05214	Tuba1a;Tuba3a	Tubulin alpha-1A chain;Tubulin alpha-3 chain	NaN	0	0	0	0	0	0
P68373	Tuba1c	Tubulin alpha-1C chain	NaN	0	0	0	0	0	0
P70168	Kpnb1	Importin subunit beta-1	NaN	0	0	0	0	0	0
P70195	Psmb7	Proteasome subunit beta type-7	NaN	0	0	0	0	0	0
P70227	Itpr3	Inositol 1,4,5-trisphosphate receptor type 3	NaN	0	0	0	0	0	0
P70333	Hnrnph2	Heterogeneous nuclear ribonucleoprotein H2	NaN	0	0	0	0	0	0
P70445	Eif4ebp2	Eukaryotic translation initiation factor 4E-binding protein 2	NaN	0	0	14234000	21372000	0	0

P70460	Vasp	Vasodilator-stimulated phosphoprotein	NaN	0	0	0	0	0	0
Q60817;P70670	Naca	mplex subunit alpha;Nascent polypeptide-associated complex subunit alpha, muscle-s	NaN	0	0	0	0	0	0
P70697;A0A0A0MQG7	Urod	Uroporphyrinogen decarboxylase	NaN	0	0	0	0	0	0
P70699;F6VEG4;A2AFL5;F6R	Gaa	Lysosomal alpha-glucosidase	NaN	0	0	0	0	0	0
Q52KG9;P80317;B1AT05;Q6	Cct6a;Cct6b	omplex protein 1 subunit zeta;T-complex protein 1 subunit zeta-2	NaN	0	0	0	0	0	0
Q3UKN6;P81117	Nucb2	Nucleobindin-2;Nesfatin-1	NaN	0	0	0	0	0	0
P83870	Phf5a	PHD finger-like domain-containing protein 5A	NaN	0	0	0	0	0	0
P84104-2;P84104;A0A3Q4EH	Srsf3	Serine/arginine-rich splicing factor 3	NaN	0	0	0	0	0	0
P97310	Mcm2	DNA replication licensing factor MCM2	NaN	0	0	0	0	0	0
Q3ULG5;P97311	Mcm6	DNA helicase;DNA replication licensing factor MCM6	NaN	0	0	0	0	0	0
P97314;A0A1W2P780;A0A1V	Csrp2	Cysteine and glycine-rich protein 2	NaN	0	0	0	0	0	0
P97346;P97346-2	Nxn	Nucleoredoxin	NaN	0	0	0	0	0	0
P97377-2;P97377	Cdk2	Cyclin-dependent kinase 2	NaN	0	0	0	0	0	0
P97384;D3Z7U0	Anxa11	Annexin A11;Annexin	NaN	0	0	0	0	0	0
Q6P1J1;P97427	Crmp1	Dihydropyrimidinase-related protein 1	NaN	0	0	0	0	0	0
P97737	Gdf10	Growth/differentiation factor 10	NaN	0	0	1468400	966510	0	0
P97819	Pla2g6	85/88 kDa calcium-independent phospholipase A2	NaN	0	0	0	0	0	0
P97825	Hn1	xpressed 1 protein;Hematological and neurological expressed 1 protein, N-terminally	NaN	0	0	0	0	1652600	1161500
P97855	G3bp1	Ras GTPase-activating protein-binding protein 1	NaN	0	0	0	0	0	0
P97864;A0A5F8MPP6	Casp7	Caspase-7;Caspase-7 subunit p20;Caspase-7 subunit p11	NaN	0	0	0	0	0	0
P99028	Uqcrh	Cytochrome b-c1 complex subunit 6, mitochondrial	NaN	0	0	0	0	0	0
V9GX00;Q00560	Il6st	Interleukin-6 receptor subunit beta	NaN	0	0	0	0	0	0
Q00PI9	Hnrnpul2	Heterogeneous nuclear ribonucleoprotein U-like protein 2	NaN	0	0	0	0	0	0
Q02013	Aqp1	Aquaporin-1	NaN	0	0	0	0	0	0
Q02248;E9Q6A9;F7CRC6;F7E	Ctnnb1	Catenin beta-1	NaN	0	0	0	0	0	0
Q04447	Ckb	Creatine kinase B-type	NaN	0	0	880470	306350	0	0
Q05186	Rcn1	Reticulocalbin-1	NaN	0	0	0	0	0	0
Q05D44	Eif5b	Eukaryotic translation initiation factor 5B	NaN	0	0	0	0	0	0
Q6PAP2;Q06806	Tie1	Tyrosine-protein kinase receptor Tie-1	NaN	0	0	0	0	0	0
Q07113	Igf2r	Cation-independent mannose-6-phosphate receptor	NaN	0	0	0	0	0	0
Q07797;E9Q5X5	Lgals3bp	Galectin-3-binding protein	NaN	0	0	0	0	0	0
Q0VBL3-2;Q0VBL3	Rbm15		NaN	0	0	0	0	0	0
Q2EG98-7;Q2EG98-6;Q2EG9	Pkd1l3	Polycystic kidney disease protein 1-like 3	NaN	0	0	0	0	0	0
Q3TBT3-3;Q3TBT3;Q3TBT3-2	Tmem173	Stimulator of interferon genes protein	NaN	0	0	0	0	0	0
Q3TC93;A0A1W2P6R8	Hs1bp3	HCLS1-binding protein 3	NaN	0	0	0	0	0	0
Q3TCN2	Plbd2	ase B-like 2 28 kDa form;Putative phospholipase B-like 2 40 kDa form;Putative phosph	NaN	0	0	0	0	0	0
Q3TFP0;Q9R0U0-3;Q9R0U0-	Srsf10	Serine/arginine-rich splicing factor 10	NaN	0	0	4623900	616970	0	0
Q6ZWQ5;Q3TGS7;Q3V2H3;C	Snx12	Sorting nexin-12	NaN	0	0	0	0	0	0
Q3THK3	Gtf2f1	General transcription factor IIF subunit 1	NaN	0	0	0	0	0	0
Q3TIV5-2;Q3TIV5	Zc3h15	Zinc finger CCCH domain-containing protein 15	NaN	0	0	0	0	0	0
Q3TQV3;Q8JZR2;Q5ND50;Q6	Crk	Adapter molecule crk	NaN	0	0	0	0	0	0
Q8BPW9;Q3TVK3;Q9Z2W0	Dnpep	Aspartyl aminopeptidase	NaN	0	0	0	0	0	0
Q3TW96;Q3TW96-2	Uap1l1	UDP-N-acetylhexosamine pyrophosphorylase-like protein 1	NaN	0	0	1667200	617300	0	0
Q3TXS7;J3QN38	Psmd1	26S proteasome non-ATPase regulatory subunit 1	NaN	0	0	0	0	0	0
Q3TYX2	Lrrn4cl	LRRN4 C-terminal-like protein	NaN	0	0	0	0	0	0

Q3U0S6	Rasip1	Ras-interacting protein 1	NaN	0	0	0	0	0	0
Q3U0V2	Tradd	or necrosis factor receptor type 1-associated DEATH domain protein	NaN	0	0	0	0	0	0
Q3U1Z5-2;Q3U1Z5;G3UYL4	Gpsm3	G-protein-signaling modulator 3	NaN	0	0	0	0	0	0
Q3U367;Q9JLJ2	Aldh9a1	4-trimethylaminobutyraldehyde dehydrogenase	NaN	0	0	0	0	0	0
Q3U4F0;Q91V61-2;Q91V61;/	Sfxn3	Sideroflexin-3	NaN	0	0	868620	1645800	0	0
Q3U741;Q501J6;Q3TU25;Q5	Ddx17	Probable ATP-dependent RNA helicase DDX17	NaN	0	0	0	0	0	0
Q3U7R1;Q3U7R1-2;A0A1W2	Esyt1	Extended synaptotagmin-1	NaN	0	0	0	0	0	0
Q3U9G9;A0A0A6YY12	Lbr	Lamin-B receptor	NaN	0	0	0	0	0	0
Q3UDD3;Q8BG81;F6VR84	Poldip3	Polymerase delta-interacting protein 3	NaN	0	0	0	0	0	0
Q3UDE2	Ttll12	Tubulin--tyrosine ligase-like protein 12	NaN	0	0	0	0	0	0
Q3UE92;Q6P1B1;A0A494BB(	Xpnpep1	Xaa-Pro aminopeptidase 1	NaN	0	0	1266500	1080200	0	0
Q3UF75;Q9EPC1	Parva	Alpha-parvin	NaN	0	0	0	0	0	0
Q3UGS4	Fam195b	Protein FAM195B	NaN	0	0	0	0	0	0
Q5SV64;Q3UH59;Q61879	Myh10	Myosin-10	NaN	0	0	0	0	0	0
Q3UH93	Plxnd1	Plexin-D1	NaN	0	0	0	0	0	0
Q3UHG5;Q62283	Tspan7	Tetraspanin;Tetraspanin-7	NaN	0	0	0	0	0	0
Q3UHK1	Slc2a13	Proton myo-inositol cotransporter	NaN	0	0	0	0	0	0
Q3UHX2	Pdap1	28 kDa heat- and acid-stable phosphoprotein	NaN	0	0	0	0	0	0
Q3UJQ9;Q9D0K2	Oxct1	oenzyme A transferase;Succinyl-CoA:3-ketoacid coenzyme A transferase 1, mitochond	NaN	0	0	0	0	0	0
Q6PD20;Q3ULD5	Mccc2	Methylcrotonoyl-CoA carboxylase beta chain, mitochondrial	NaN	0	0	0	0	0	0
Q3UM45;A0A087WRA7	Ppp1r7	Protein phosphatase 1 regulatory subunit 7	NaN	664150	9758.1	0	0	0	0
Q3UMT1;F6XWD4	Ppp1r12c	Protein phosphatase 1 regulatory subunit 12C	NaN	0	0	0	0	0	0
S4R2A9;Q3UPL0-2;Q3UPL0;S	Sec31a	Protein transport protein Sec31A	NaN	0	0	3466700	5565100	0	0
Q3URS9-2;Q3URS9	Ccdc51	Coiled-coil domain-containing protein 51	NaN	0	0	0	0	0	0
Q3V038	Ttc9	Tetratricopeptide repeat protein 9A	NaN	0	0	0	0	0	0
Q3V117;Q91V92;Q3TS02	Acly	ATP-citrate synthase	NaN	0	0	0	0	0	0
Q3V3R1	Mthfd1l	Monofunctional C1-tetrahydrofolate synthase, mitochondrial	NaN	0	0	0	0	0	0
Q4PZA2-3;Q4PZA2-2;Q4PZA2	Ece1	Endothelin-converting enzyme 1	NaN	0	0	0	0	0	0
Q542V3;Q8VE97	Srsf4	Serine/arginine-rich splicing factor 4	NaN	0	0	0	0	0	0
Q58A65-5;Q58A65-2;Q58A6!	Spag9	C-Jun-amino-terminal kinase-interacting protein 4	NaN	0	0	0	0	0	0
Q5DP50	Tex24		NaN	0	0	0	0	0	0
Q5F258;Q68FF6	Git1	ARF GTPase-activating protein GIT1	NaN	0	0	0	0	0	0
Q5F284;Q9JIZ9	Plscr3	Phospholipid scramblase 3	NaN	0	0	0	0	0	0
Q5SQB0;Q61937;Q9DAY9;Q5	Npm1	Nucleophosmin	NaN	0	0	0	0	0	0
Q5SXA5;Q5SRX1-3;Q5SRX1-4	Tom1l2	TOM1-like protein 2	NaN	0	0	0	0	0	0
Q5SUH7;Q5SUH6;Q99KN9-2	Clint1	Clathrin interactor 1	NaN	0	0	0	0	0	0
Q5SUR0	Pfas	Phosphoribosylformylglycinamidine synthase	NaN	0	0	0	0	0	0
Q5SUT0;Q5SUS9;Q61545;Q5	Ewsr1	RNA-binding protein EWS	NaN	0	0	0	0	0	0
Q5SWZ5;F6S5I0;F6RND9;P9	Mprip	Myosin phosphatase Rho-interacting protein	NaN	0	0	1174500	3636900	0	0
Q5SX75;Q60716-2;Q60716;B	P4ha2	Prolyl 4-hydroxylase subunit alpha-2	NaN	0	0	0	0	0	0
Q5U458;E9Q8B3	Dnajc11	DnaJ homolog subfamily C member 11	NaN	0	0	0	0	0	0
Q5U4C5;Q8VI75	Ipo4	Importin-4	NaN	0	0	0	0	0	0
Q5U4D9	Thoc6	THO complex subunit 6 homolog	NaN	0	0	0	0	0	0
Q60876	Eif4ebp1	Eukaryotic translation initiation factor 4E-binding protein 1	NaN	0	0	0	0	0	0
Q61001	Lama5	Laminin subunit alpha-5	NaN	0	0	0	0	0	0

Q61033;Q61033-2;Q61029-4	Tmpo	2, isoforms alpha/zeta;Lamina-associated polypeptide 2, isoforms beta/delta/epsilon	NaN	0	0	0	0	0	0
Q61140-2;Q61140	Bcar1	Breast cancer anti-estrogen resistance protein 1	NaN	0	0	0	0	0	0
Q61160	Fadd	FAS-associated death domain protein	NaN	0	0	0	0	0	0
Q61166	Mapre1	Microtubule-associated protein RP/EB family member 1	NaN	0	0	0	0	0	0
Q61249	Igbp1	Immunoglobulin-binding protein 1	NaN	0	0	0	0	0	0
Q61292	Lamb2	Laminin subunit beta-2	NaN	0	0	0	0	0	0
Q61550	Rad21	Double-strand-break repair protein rad21 homolog	NaN	0	0	0	0	0	0
Q61753;F6ZSB7	Phgdh	D-3-phosphoglycerate dehydrogenase	NaN	0	0	0	0	0	0
Q61768;E9QAK5	Kif5b	Kinesin-1 heavy chain;Kinesin-like protein	NaN	0	0	0	0	0	0
Q61881;D3Z6N3	Mcm7	DNA replication licensing factor MCM7	NaN	0	0	0	0	0	0
Q62093	Srsf2	Serine/arginine-rich splicing factor 2	NaN	0	0	0	0	0	0
Q62095	Ddx3y	ATP-dependent RNA helicase DDX3Y	NaN	0	0	817250	469520	0	0
Q62159;A0A0G2JEP8;H3BL5(	Rhoc;Rhoa;4930544G11Rik;Rhob	ling protein RhoC;Transforming protein RhoA;Rho-related GTP-binding protein RhoB	NaN	0	0	0	0	0	0
Q9D8L3;Q62186	Ssr4	Translocon-associated protein subunit delta	NaN	0	0	1217200	1294600	0	0
Q62312-2;Q62312	Tgfbr2	TGF-beta receptor type-2	NaN	0	0	0	0	0	0
Q8C872;Q62351	Tfrc	Transferrin receptor protein 1	NaN	0	0	0	0	0	0
Q62376;A0A1B0GSX5;A0A1E	Snrnp70	U1 small nuclear ribonucleoprotein 70 kDa	NaN	0	0	0	0	0	0
Q62433;E9Q514	Ndrg1	Protein NDRG1	NaN	6981300	0	0	0	0	0
Q62470;Q62470-2;Q62470-3	Itga3	rin alpha-3;Integrin alpha-3 heavy chain;Integrin alpha-3 light chain	NaN	0	0	0	0	0	0
Q7TQE2;Q62523;A0A0N4SVI	Zyx	Zyxin	NaN	0	0	0	0	0	0
Q63829	Commd3	COMM domain-containing protein 3	NaN	0	0	0	0	0	0
Q64152-2;Q64152	Btf3	Transcription factor BTF3	NaN	0	0	0	0	0	0
Q64282	Ifit1	Interferon-induced protein with tetratricopeptide repeats 1	NaN	0	0	0	0	0	0
Q64387;Q64387-2	Pnoc	Prepronociceptin;Neuropeptide 1;Nociceptin;Neuropeptide 2	NaN	0	0	0	0	13336000	1109700
Q64669	Nqo1	NAD(P)H dehydrogenase [quinone] 1	NaN	0	0	0	0	0	0
Q64727;A0A286YDJ4	Vcl	Vinculin	NaN	0	0	0	0	0	0
Q64735-2;Q64735	Cr1l	Complement component receptor 1-like protein	NaN	0	0	0	0	0	0
Q64737;Q64737-2	Gart	oribosylamine--glycine ligase;Phosphoribosylformylglycinamidine cyclo-ligase;Phospho	NaN	0	0	0	0	0	0
Q6A0A2-2;Q6A0A2	Larp4b	La-related protein 4B	NaN	0	0	0	0	0	0
Q6A0A9	FAM120A	Constitutive coactivator of PPAR-gamma-like protein 1	NaN	0	0	0	0	0	0
Q6DFV5;Q6DFV5-3	Helz	Probable helicase with zinc finger domain	NaN	0	0	0	0	1446900	1063900
Q6DVA0	Lemd2	LEM domain-containing protein 2	NaN	0	0	0	0	0	0
Q6GQT9	Nomo1	Nodal modulator 1	NaN	0	0	0	0	0	0
Q6P4T2	Snrnp200	U5 small nuclear ribonucleoprotein 200 kDa helicase	NaN	0	0	0	0	0	0
Q6P542	Abcf1	ATP-binding cassette sub-family F member 1	NaN	0	0	0	0	0	0
Q6P5B5;Q9WVR4	Fxr2	Fragile X mental retardation syndrome-related protein 2	NaN	0	0	1846400	1001900	0	0
Q6P5F7-2;Q6P5F7	Ttyh3	Protein tweety homolog 3	NaN	0	0	0	0	0	0
Q6P5H2-2;Q6P5H2	Nes	Nestin	NaN	0	0	0	0	0	0
Q6P9J5	Kank4	KN motif and ankyrin repeat domain-containing protein 4	NaN	0	0	0	0	0	0
Q6PAM1;A8Y5J8;A2ADZ3;A2	Txlna	Alpha-taxilin	NaN	0	0	1175400	1468100	0	0
Q6PB44-2;Q6PB44	Ptpn23	Tyrosine-protein phosphatase non-receptor type 23	NaN	0	0	0	0	0	0
Q6PB66	Lrpprc	Leucine-rich PPR motif-containing protein, mitochondrial	NaN	0	0	0	0	0	0
Q6PD28	Ppp2r5b		NaN	0	0	0	0	0	0
Q6PF96;Q921G7	Etfdh	ron transfer flavoprotein-ubiquinone oxidoreductase, mitochondrial	NaN	0	0	0	0	0	0
Q6PGH2	Hn1l	Hematological and neurological expressed 1-like protein	NaN	0	0	0	0	0	0

Q6PGL7-2;Q6PGL7	Fam21	WASH complex subunit FAM21	NaN	0	0	0	0	0	0
Q6R891-2;Q6R891	Ppp1r9b	Neurabin-2	NaN	0	0	0	0	0	0
Z4YJT3;Q6ZQ58;Q6ZQ58-2	Larp1	La-related protein 1	NaN	0	0	0	0	0	0
Q6ZQ73	Cand2	Cullin-associated NEDD8-dissociated protein 2	NaN	0	0	647670	1505100	0	0
Q6ZWM4;A0A0N4SUZ3	Lsm8	U6 snRNA-associated Sm-like protein LSm8	NaN	0	0	0	0	0	0
Q6ZWY8	Tmsb10	Thymosin beta-10	NaN	4753000	866680	0	0	0	0
Q6ZWZ4;P47964	Rpl36	60S ribosomal protein L36	NaN	0	0	0	0	0	0
Q70IV5-2;Q70IV5;A0A140LJ7	Synm	Synemin	NaN	0	0	0	0	0	0
Q76MZ3;H3BIV7;H3BLQ0;H3	Ppp2r1a;Ppp2r1b	ulatory subunit A alpha isoform;Serine/threonine-protein phosphatase 2A 65 kDa regu	NaN	0	0	343620	293520	0	0
Q7TQH0-2;Q3TGG2;A0A0U1	Atxn2l	Ataxin-2-like protein	NaN	0	0	0	0	0	0
Q7TRE1;Q7TRE0	Olfr884;Olfr885		NaN	0	0	0	0	0	0
Q7TSV4	Pgm2	Phosphoglucomutase-2	NaN	0	0	0	0	0	0
Q7TT50;A0A1Y7VM95	Cdc42bpb	Serine/threonine-protein kinase MRCK beta	NaN	0	0	0	0	1519200	1296900
Q80UU9	Pgrmc2	Membrane-associated progesterone receptor component 2	NaN	0	0	0	0	0	0
Q80VP0-2;Q80VP0	Tecpr1	Tectonin beta-propeller repeat-containing protein 1	NaN	0	0	0	0	0	0
Q80XI4	Pip4k2b	Phosphatidylinositol 5-phosphate 4-kinase type-2 beta	NaN	0	0	0	0	0	0
Q80YX1-2;Q80YX1;Q80YX1-5	Tnc	Tenascin	NaN	0	0	0	0	0	0
Q80ZA0	Itln1b	Intelectin-1b	NaN	0	0	0	0	0	0
Q80ZE3	Siglecg		NaN	0	0	0	0	0	0
Q80ZS3	Mrps26	28S ribosomal protein S26, mitochondrial	NaN	0	0	0	0	0	0
Q80ZX0;F6VJC5	Sec24b		NaN	0	0	0	0	0	0
Q810B6	Ankfy1	Rabankyrin-5	NaN	0	0	0	0	0	0
Q8BFQ8	Pddc1	Parkinson disease 7 domain-containing protein 1	NaN	0	0	0	0	0	0
Q8BFR4	Gns	N-acetylglucosamine-6-sulfatase	NaN	0	0	0	0	0	0
Q8BFZ3	Actbl2	Beta-actin-like protein 2	NaN	0	0	0	0	0	0
Q8BG32;G3UZ28;G3UYI4;G3	Psmd11	26S proteasome non-ATPase regulatory subunit 11	NaN	0	0	0	0	0	0
Q8BGT1	Flrt3		NaN	0	0	0	0	0	0
Q8BH43;B1AUN0	Wasf2	Wiskott-Aldrich syndrome protein family member 2	NaN	0	0	0	0	0	0
Q8BHC7	Rhbdd1	Rhomboid-related protein 4	NaN	0	0	0	0	0	0
Q8BHL8	Psmf1	Proteasome inhibitor PI31 subunit	NaN	0	0	0	0	0	0
Q8BHN3;Q8BHN3-2;Q8BHN3	Ganab	Neutral alpha-glucosidase AB	NaN	0	0	0	0	0	0
Q8BII1	Prox2	Prospero homeobox protein 2	NaN	0	769140	0	0	0	0
Q8BIJ7	Rufy1	RUN and FYVE domain-containing protein 1	NaN	0	0	0	0	0	0
Q8BJ05-2;Q8BJ05-3;Q8BJ05	Zc3h14	Zinc finger CCCH domain-containing protein 14	NaN	0	0	0	0	0	0
Q8BJS4-3;Q8BJS4-2;Q8BJS4	Sun2	SUN domain-containing protein 2	NaN	0	0	0	0	0	0
Q8BJY1;F7BA91	Psmd5	26S proteasome non-ATPase regulatory subunit 5	NaN	0	0	0	0	0	0
Q8BK64	Ahsa1	Activator of 90 kDa heat shock protein ATPase homolog 1	NaN	0	0	0	0	0	0
Q8BL66	Eea1	Early endosome antigen 1	NaN	0	0	0	0	0	0
Q8BMF4	Dlat	due acetyltransferase component of pyruvate dehydrogenase complex, mitochondrial	NaN	0	0	0	0	0	0
Q8BN82-3;Q8BN82-2;Q8BN8	Slc17a5	Sialin	NaN	0	0	0	0	0	0
Q8BPG6	Sumf2	Sulfatase-modifying factor 2	NaN	0	0	0	0	0	0
Q8BPU7-3;Q8BPU7	Elmo1	Engulfment and cell motility protein 1	NaN	0	0	0	0	0	0
Q8BQ47;D3Z0T5	Cnpy4	Protein canopy homolog 4	NaN	0	0	0	0	0	0
Q8BT60;Q9D6C8;A0A0R4J1D	;Cpne4;Cpne6;Cpne2;Cpne9;Cpne7;Cpne8;	e-3;Copine-2;Copine-9;Copine-6;Copine-4;Copine-7;Copine-8;Copine-5	NaN	0	0	0	0	0	0
Q8BTI8-3;Q8BTI8-2;Q8BTI8;/	Srrm2	Serine/arginine repetitive matrix protein 2	NaN	0	0	0	0	0	0

Q8BTW3	Exosc6	Exosome complex component MTR3	NaN	0	0	0	0	0	0
Q8BTZ7	Gmppb	Mannose-1-phosphate guanyltransferase beta	NaN	0	0	0	0	0	0
Q8BU62	Atp10d		NaN	0	0	0	0	0	0
Q8BUE4;Q8BUE4-2	Aifm2	Apoptosis-inducing factor 2	NaN	0	0	1473500	1329600	0	0
Q8BUK6;F6W1A2	Hook3	Protein Hook homolog 3	NaN	0	0	0	0	0	0
Q8BUR4	Dock1	Dedicator of cytokinesis protein 1	NaN	0	0	0	0	0	0
Q8BVA0;Q99P91	Gpnmb	Transmembrane glycoprotein NMB	NaN	0	0	0	0	0	0
Q99MI6;Q8BWF2	Gimap3;Gimap5	GTPase IMAP family member 3;GTPase IMAP family member 5	NaN	0	0	0	0	0	0
Q8BYZ1	Abi3	ABI gene family member 3	NaN	0	0	0	0	0	0
Q8BZR9		Uncharacterized protein C17orf85 homolog	NaN	0	0	0	0	0	0
Q8C0E3;Q8C0E3-2	Trim47	Tripartite motif-containing protein 47	NaN	0	0	0	0	0	0
Q8C2Q3;E9QL13;Q8C2Q3-2;J	Rbm14	RNA-binding protein 14	NaN	0	0	1485500	4890600	0	0
Q8C3W1		Uncharacterized protein C1orf198 homolog	NaN	0	0	0	0	0	0
Q8C3X8	Lmf2	Lipase maturation factor 2	NaN	0	0	0	0	0	0
Q8C522	Endod1	Endonuclease domain-containing 1 protein	NaN	0	0	0	0	0	0
Q8C7E9;A2AEJ8;F6ZKC7;A2A	Cstf2t;Cstf2	mulation factor subunit 2 tau variant;Cleavage stimulation factor subunit 2	NaN	264860	4266000	0	0	0	0
Q8C8U0-2;Q8C8U0;Q8C8U0-	Ppfibp1;Ppfibp2	Liprin-beta-1;Liprin-beta-2	NaN	0	0	0	0	0	0
Q8CBY8-2;Q8CBY8	Dctn4	Dynactin subunit 4	NaN	0	0	0	0	0	0
Q8CG76	Akr7a2	Aflatoxin B1 aldehyde reductase member 2	NaN	0	0	0	0	0	0
Q8CGA0	Ppm1f	Protein phosphatase 1F	NaN	0	0	0	0	0	0
Q8CGC7;A0A0A6YWH3;A0A0	Eprs	lutamate/proline--tRNA ligase;Glutamate--tRNA ligase;Proline--tRNA ligase	NaN	0	0	0	0	0	0
Q8CGK3	Lonp1	Lon protease homolog, mitochondrial	NaN	0	0	0	0	0	0
Q8CHT0	Aldh4a1	Delta-1-pyrroline-5-carboxylate dehydrogenase, mitochondrial	NaN	0	0	0	0	0	0
Q8CHU3;F7CD65;F7CUV7;Q5	Epn2	Epsin-2	NaN	0	0	0	0	0	0
Q8CI08;Q8CI08-2	Slain2	SLAIN motif-containing protein 2	NaN	0	0	0	0	0	0
Q8JZK9	Hmgcs1	Hydroxymethylglutaryl-CoA synthase, cytoplasmic	NaN	0	0	0	0	0	0
Q8K078-2;Q8K078	Slco4a1	Solute carrier organic anion transporter family member 4A1	NaN	0	0	0	0	0	0
Q8K0B2-3;Q8K0B2-2;Q8K0B2	Lmbrd1	Probable lysosomal cobalamin transporter	NaN	0	0	0	0	0	0
Q8K0H5	Taf10	Transcription initiation factor TFIID subunit 10	NaN	0	0	0	0	0	0
Q8K2C7-2;Q8K2C7	Os9	Protein OS-9	NaN	0	0	0	0	0	0
Q8K2Y3	Eva1b	Protein eva-1 homolog B	NaN	0	0	0	0	0	0
Q8K3J1	Ndufs8	H dehydrogenase [ubiquinone] iron-sulfur protein 8, mitochondrial	NaN	0	0	0	0	0	0
Q8QZT1	Acat1	Acetyl-CoA acetyltransferase, mitochondrial	NaN	0	0	0	0	0	0
Q8QZY1	Eif3l	Eukaryotic translation initiation factor 3 subunit L	NaN	0	0	0	0	0	0
Q8R010	Aimp2	noacyl tRNA synthase complex-interacting multifunctional protein 2	NaN	0	0	0	0	0	0
Q8R059	Gale	UDP-glucose 4-epimerase	NaN	0	0	0	0	0	0
Q8R0G9	Nup133	Nuclear pore complex protein Nup133	NaN	0	0	0	0	0	0
Q8R0T6;A0A1D5RLS0	Gpr97	Probable G-protein coupled receptor 97	NaN	0	0	0	0	0	0
Q8R1V4;Q99KF1	Tmed4;Tmed9	24 domain-containing protein 4;Transmembrane emp24 domain-containing protein 9	NaN	0	0	0	0	0	0
Q8R2Q8	Bst2	Bone marrow stromal antigen 2	NaN	0	0	0	0	0	0
Q8R2U6	Nudt4	Diphosphoinositol polyphosphate phosphohydrolase 2	NaN	0	0	0	0	0	0
Q8R307	Vps18	Vacuolar protein sorting-associated protein 18 homolog	NaN	0	0	0	0	0	0
Q8R317;Q8R317-2	Ubqln1	Ubiquilin-1	NaN	0	0	0	0	0	0
Q8R4R6;A2ATJ2	Nup35	Nucleoporin NUP53	NaN	0	0	0	0	0	0
Q8R570	Snap47	Synaptosomal-associated protein 47	NaN	0	0	0	0	0	0

Q8VBV7;A0A087WPM5	Cops8	COP9 signalosome complex subunit 8	NaN	0	0	0	0	0	0
Q8VC30	Dak	one kinase/FAD-AMP lyase (cyclizing);ATP-dependent dihydroxyacetone kinase;FAD-A	NaN	0	0	0	0	0	0
Q8VCF0	Mavs	Mitochondrial antiviral-signaling protein	NaN	0	0	0	0	0	0
Q9CQ43;Q8VCG1	Dut		NaN	0	0	0	0	0	0
Q8VCR4;Q9DCG9	Trmt112	Multifunctional methyltransferase subunit TRM112-like protein	NaN	0	0	0	0	0	0
Q8VCV2;Q9QYF9	Ndrg3	Protein NDRG3	NaN	0	0	0	0	0	0
Q8VD75;A0A0J9YV08;A0A1D	Hip1	Huntingtin-interacting protein 1	NaN	0	0	0	0	0	0
Q8VDM4	Psmd2	26S proteasome non-ATPase regulatory subunit 2	NaN	4565700	395720	0	0	0	0
Q8VDP3-2;Q8VDP3-3;Q8VDP	Mical1	Protein-methionine sulfoxide oxidase MICAL1	NaN	0	0	0	0	0	0
Q8VE99	Ccdc115	Coiled-coil domain-containing protein 115	NaN	0	0	0	0	0	0
Q8VEE1	Lmcd1	LIM and cysteine-rich domains protein 1	NaN	0	0	0	0	0	0
Q91V12-2;Q91V12-4;E9PYH2	Acot7	Cytosolic acyl coenzyme A thioester hydrolase	NaN	0	0	0	0	0	0
Q91VD9	Ndufs1	NADH-ubiquinone oxidoreductase 75 kDa subunit, mitochondrial	NaN	0	0	1268600	1530800	0	0
Q91VH6;A0A3B2WCC1;A0A3	Memo1	Protein MEMO1	NaN	0	0	0	0	0	0
Q91VR5;A0A1Y7VM48	Ddx1	ATP-dependent RNA helicase DDX1	NaN	696940	1019700	0	0	0	0
Q91WC0-3;Q91WC0-2;Q91W	Setd3	Histone-lysine N-methyltransferase setd3	NaN	0	0	0	0	0	0
Q91WG2-2;Q91WG2-1;Q91\	Rabep2	Rab GTPase-binding effector protein 2	NaN	0	0	0	0	0	0
Q91YP2	Nln	Neurolysin, mitochondrial	NaN	0	0	790550	325820	0	0
Q91YQ5;A0A0N4SUJ8	Rpn1	nyl-diphosphooligosaccharide--protein glycosyltransferase subunit 1	NaN	0	0	0	0	0	0
Q91YR1	Twf1	Twinfilin-1	NaN	0	0	0	0	0	0
Q91YT8	Tmem63a	CSC1-like protein 1	NaN	0	0	0	0	0	0
Q91YW3	Dnajc3	DnaJ homolog subfamily C member 3	NaN	0	0	0	0	0	0
Q91Z96-2;Q91Z96	Bmp2k	BMP-2-inducible protein kinase	NaN	609870	1025900	0	0	0	0
Q91ZR1	Rab4b	Ras-related protein Rab-4B	NaN	0	0	0	0	0	0
Q921I1	Tf	Serotransferrin	NaN	0	0	0	0	0	0
Q921M3-2;Q921M3	Sf3b3	Splicing factor 3B subunit 3	NaN	0	0	0	0	0	0
Q922F4	Tubb6	Tubulin beta-6 chain	NaN	0	0	0	0	0	0
Q922Q1	02-Mar	Mitochondrial amidoxime reducing component 2	NaN	0	0	0	0	0	0
Q922Q4	Pycr2	Pyrroline-5-carboxylate reductase 2	NaN	0	0	0	0	0	0
Q922Q8	Lrrc59	Leucine-rich repeat-containing protein 59	NaN	0	0	0	0	0	0
Q923D5	Wbp11	WW domain-binding protein 11	NaN	0	0	0	0	0	0
Q925B0-2;Q925B0	Pawr	PRKC apoptosis WT1 regulator protein	NaN	0	0	15850000	0	114600000	0
Q925F2;D3Z5Y0	Esam	Endothelial cell-selective adhesion molecule	NaN	0	0	0	0	0	0
Q99J36	Thumpd1	THUMP domain-containing protein 1	NaN	0	0	0	0	0	0
Q99JB2;A2AG41;A2AG39;F6'	Stoml2	Stomatin-like protein 2, mitochondrial	NaN	1872300	585780	0	0	0	0
Q99JP6;Q99JP6-2;J3QQ00	Homer3	Homer protein homolog 3	NaN	0	0	0	0	0	0
Q99JR1	Sfxn1	Sideroflexin-1	NaN	0	0	0	0	0	0
Q99JX4	Eif3m	Eukaryotic translation initiation factor 3 subunit M	NaN	0	0	0	0	0	0
V9GX96;V9GXF3;Q99JX7	Nxf1	Nuclear RNA export factor 1	NaN	0	0	0	0	0	0
Q99JY9;A0A087WRA1;A0A0	Actr3	Actin-related protein 3	NaN	0	0	0	0	0	0
Q99K41	Emilin1	EMILIN-1	NaN	0	0	0	0	0	0
Q99K48;Q99K48-2;B1AXT0	Nono	Non-POU domain-containing octamer-binding protein	NaN	0	0	0	0	0	0
Q99KC8	Vwa5a	von Willebrand factor A domain-containing protein 5A	NaN	0	0	0	0	0	0
Q99KE1	Me2	NAD-dependent malic enzyme, mitochondrial	NaN	0	0	0	0	0	0
Q99KI0;A0A2R8VJW0	Aco2	Aconitate hydratase, mitochondrial	NaN	0	0	3828100	1474900	0	0

Q99KJ8	Dctn2	Dynactin subunit 2	NaN	0	0	0	0	0	0
Q99KK7;A0A494BBC1;A0A49	Dpp3	Dipeptidyl peptidase 3	NaN	0	0	0	0	0	0
Q99KQ4	Nampt	Nicotinamide phosphoribosyltransferase	NaN	0	0	0	0	0	0
Q99KV1	Dnajb11	DnaJ homolog subfamily B member 11	NaN	0	0	0	0	0	0
Q99L45;E0CXJ3	Eif2s2	Eukaryotic translation initiation factor 2 subunit 2	NaN	0	0	831350	343090	0	0
Q99LB4;P24452;D3YZN3;D3Y	Capg	Macrophage-capping protein	NaN	0	0	1419900	1670100	0	0
Q99LJ0	Cttnbp2nl	CTTNBP2 N-terminal-like protein	NaN	0	0	0	0	0	0
Q99LN9;D3Z7J7;D3Z6Y9;Q99	Dohh	Deoxyhypusine hydroxylase	NaN	0	0	0	0	0	0
Q99M08		Uncharacterized protein C4orf3 homolog	NaN	0	0	0	0	0	0
Q99M71-2;Q99M71	Epdr1	Mammalian ependymin-related protein 1	NaN	0	0	0	0	0	0
Q99M87-3;Q99M87-2;Q99M	Dnaja3	DnaJ homolog subfamily A member 3, mitochondrial	NaN	0	0	0	0	0	0
Q99MR3	Slc12a9	Solute carrier family 12 member 9	NaN	0	0	0	0	0	0
Q99MR6-3;Q99MR6-4;Q99M	Srrt	Serrate RNA effector molecule homolog	NaN	0	0	0	0	0	0
Q99NB8	Ubqln4	Ubiquilin-4	NaN	0	0	0	0	0	0
Q99P72-5;Q99P72-4;Q99P72	Rtn4	Reticulon-4	NaN	0	0	0	0	0	0
Q99PV0;B7ZC27	Prpf8	Pre-mRNA-processing-splicing factor 8	NaN	0	0	0	0	0	0
Q9CPP0	Npm3	Nucleoplasmin-3	NaN	0	0	0	0	0	0
Q9CPQ3;A0A2R8VHM4	Tomm22	Mitochondrial import receptor subunit TOM22 homolog	NaN	0	0	0	0	0	0
Q9CPS7	Pno1	RNA-binding protein PNO1	NaN	0	0	0	0	0	0
Q9CPY7-2;Q9CPY7;A0A0G2JE	Lap3	Cytosol aminopeptidase	NaN	0	0	0	0	0	0
Q9CPZ6	Ormdl3	ORM1-like protein 3	NaN	0	0	0	0	0	0
Q9CQ65	Mtap	S-methyl-5-thioadenosine phosphorylase	NaN	0	0	1983100	417110	0	0
Q9CQ69	Uqcrq	Cytochrome b-c1 complex subunit 8	NaN	0	0	0	0	0	0
Q9CQ71	Rpa3	Replication protein A 14 kDa subunit	NaN	0	0	0	0	0	0
Q9CQE8		UPF0568 protein C14orf166 homolog	NaN	0	0	0	0	3869800	505170
Q9CQI6	Cotl1	Coactosin-like protein	NaN	0	0	1017100	721460	0	0
Q9CQI7	Snrpb2	U2 small nuclear ribonucleoprotein B	NaN	0	0	0	0	0	0
Q9CQN1	Trap1	Heat shock protein 75 kDa, mitochondrial	NaN	0	0	0	0	0	0
Q9CQQ4	Gemin2	Gem-associated protein 2	NaN	0	0	0	0	0	0
Q9CQV4-2;Q9CQV4	Fam134c	Protein FAM134C	NaN	0	0	0	0	0	0
Q9CR00;A0A0G2JGN6	Psmd9	26S proteasome non-ATPase regulatory subunit 9	NaN	0	0	0	0	0	0
Q9CR41	Hypk	Huntingtin-interacting protein K	NaN	0	0	0	0	0	0
Q9CR57;A0A1L1SUF6	Rpl14	60S ribosomal protein L14	NaN	0	0	0	0	0	0
Q9CR86	Carhsp1	Calcium-regulated heat stable protein 1	NaN	0	0	0	0	0	0
Q9CRD2	Emc2	ER membrane protein complex subunit 2	NaN	0	0	0	0	0	0
Q9CU62	Smc1a	Structural maintenance of chromosomes protein 1A	NaN	0	0	0	0	3175400	0
Q9CW03	Smc3	Structural maintenance of chromosomes protein 3	NaN	0	0	0	0	0	0
Q9CWK3;A0A0U1RP07;A0A0	Cd2bp2	CD2 antigen cytoplasmic tail-binding protein 2	NaN	0	0	0	0	0	0
Q9CWL8	Ctnnbl1	Beta-catenin-like protein 1	NaN	0	0	0	0	0	0
Q9CX00	Ist1	IST1 homolog	NaN	0	0	0	0	0	0
Q9CX34	Sugt1	Suppressor of G2 allele of SKP1 homolog	NaN	0	0	0	0	0	0
Q9CX60;F6UVG6	Lbh	Protein LBH	NaN	0	0	0	0	0	0
Q9CX86	Hnrnpa0	Heterogeneous nuclear ribonucleoprotein A0	NaN	0	0	0	0	0	0
Q9CY97-2;Q9CY97	Ssu72	A polymerase II subunit A C-terminal domain phosphatase SSU72	NaN	0	0	0	0	0	0
Q9CYG7;Q9CYG7-2	Tomm34	Mitochondrial import receptor subunit TOM34	NaN	0	0	0	0	0	0

Q9CYH6	Rrs1	Ribosome biogenesis regulatory protein homolog	NaN	0	0	0	0	0	0
Q9CYL5	Glipr2	Golgi-associated plant pathogenesis-related protein 1	NaN	0	0	0	0	0	0
Q9CYN9	Atp6ap2	Renin receptor	NaN	0	0	0	0	0	0
Q9CZ04;Q9CZ04-2	Cops7a	COP9 signalosome complex subunit 7a	NaN	0	0	0	0	0	0
Q9CZ13;A0A0A6YWX6;A0A0.	Uqcrc1	Cytochrome b-c1 complex subunit 1, mitochondrial	NaN	996990	1142500	0	0	0	0
Q9CZ44-2;Q9CZ44;Q9CZ44-3	Nsfl1c	NSFL1 cofactor p47	NaN	0	0	0	0	0	0
Q9ERR1-2;Q9CZA6-3;Q9CZA(	Ndel1;Nde1	:tribution protein nudE-like 1;Nuclear distribution protein nudE homolog 1	NaN	0	0	0	0	0	0
Q9CZC8;A0A0N4SWD0	Scrn1	Secernin-1	NaN	0	0	0	0	0	0
Q9CZE3	Rab32	Ras-related protein Rab-32	NaN	0	0	0	0	0	0
Q9CZJ2;H7BX84;Q8K0U4	Hspa12b	Heat shock 70 kDa protein 12B	NaN	0	0	0	0	0	0
Q9CZR8	Tsfm	Elongation factor Ts, mitochondrial	NaN	0	0	0	0	0	0
Q9CZW5	Tomm70a	Mitochondrial import receptor subunit TOM70	NaN	0	0	0	0	0	0
Q9D051	Pdhb	ruvate dehydrogenase E1 component subunit beta, mitochondrial	NaN	0	0	0	0	0	0
Q9D0F3	Lman1	Protein ERGIC-53	NaN	0	0	0	0	0	0
Q9D0I9	Rars	Arginine--tRNA ligase, cytoplasmic	NaN	0	0	849870	2228000	0	0
Q9D0J4	Arl2	ADP-ribosylation factor-like protein 2	NaN	0	0	0	0	0	0
Q9D0L8-2;Q9D0L8	Rnmt	mRNA cap guanine-N7 methyltransferase	NaN	0	0	0	0	0	0
Q9D0M3-2;Q9D0M3;A0A2R8	Cyc1	Cytochrome c1, heme protein, mitochondrial	NaN	0	0	0	0	0	0
Q9D0R2;A0A2I3BPK8	Tars	Threonine--tRNA ligase, cytoplasmic	NaN	0	0	1054000	4800500	0	0
Q9D0S9	Hint2	Histidine triad nucleotide-binding protein 2, mitochondrial	NaN	0	0	0	0	0	0
Q9D1I2		Bcl10-interacting CARD protein	NaN	0	0	0	0	0	0
Q9D1M0	Sec13	Protein SEC13 homolog	NaN	0	0	0	0	0	0
Q9D1Q4	Dpm3	Dolichol-phosphate mannosyltransferase subunit 3	NaN	0	0	0	0	0	0
Q9D1Q6	Erp44	Endoplasmic reticulum resident protein 44	NaN	0	0	0	0	0	0
Q9D2G2-2;Q9D2G2	Dlst	succinyltransferase component of 2-oxoglutarate dehydrogenase complex, mitochonc	NaN	0	0	0	0	0	0
Q9D2R0	Aacs	Acetoacetyl-CoA synthetase	NaN	0	0	0	0	0	0
Q9D554	Sf3a3	Splicing factor 3A subunit 3	NaN	0	0	0	0	0	0
Q9D662;A2ANA0;A2AN97;A2	Sec23b	Protein transport protein Sec23B	NaN	0	0	0	0	0	0
Q9D6V8	Paip2	Polyadenylate-binding protein-interacting protein 2	NaN	0	0	0	0	0	0
Q9D6Z1;F6USW7;F6U250;F6	Nop56	Nucleolar protein 56	NaN	0	0	1001300	1326900	0	0
Q9D7E4		UPF0449 protein C19orf25 homolog	NaN	0	0	0	0	0	0
Q9D7S7-2;Q9D7S7	Rpl22l1	60S ribosomal protein L22-like 1	NaN	0	0	0	0	0	0
Q9D7S9	Chmp5	Charged multivesicular body protein 5	NaN	0	0	0	0	0	0
Q9D819	Ppa1	Inorganic pyrophosphatase	NaN	0	0	0	0	908640	875110
Q9D880	Timm50	litochondrial import inner membrane translocase subunit TIM50	NaN	0	0	0	0	0	0
Q9D883;A0A494B947;G3UW	U2af1	Splicing factor U2AF 35 kDa subunit	NaN	0	0	0	0	0	0
Q9D8U8	Snx5	Sorting nexin-5	NaN	0	0	0	0	0	0
Q9D8X2	Ccdc124	Coiled-coil domain-containing protein 124	NaN	0	0	0	0	0	0
Q9D967	Mdp1	Magnesium-dependent phosphatase 1	NaN	0	0	0	0	0	0
Q9DAK9	Phpt1	14 kDa phosphohistidine phosphatase	NaN	0	0	0	0	0	0
Q9DB73	Cyb5r1	NADH-cytochrome b5 reductase 1	NaN	0	0	0	0	0	0
Q9DB77;A0A140LI98	Uqcrc2	Cytochrome b-c1 complex subunit 2, mitochondrial	NaN	0	0	0	0	0	0
Q9DBC7;D3Z0V6;P12849	Prkar1a;Prkar1b	ndent protein kinase type I-alpha regulatory subunit, N-terminally processed;cAMP-dep	NaN	0	0	0	0	0	0
Q9DBG5;A0A3B2WCW2	Plin3	Perilipin-3	NaN	0	0	3910600	886150	0	0
Q9DBH5	Lman2	Vesicular integral-membrane protein VIP36	NaN	0	0	0	0	0	0

Q9DBL2;Q9DBL2-2	Gdap2	Ganglioside-induced differentiation-associated protein 2	NaN	0	0	0	1435000	0	1467600
Q9DBR0	Akap8	A-kinase anchor protein 8	NaN	0	0	0	0	0	0
Q9DBR1-2;Q9DBR1	Xrn2	5-3 exoribonuclease 2	NaN	0	0	0	0	0	0
Q9DBS5	Klc4	Kinesin light chain 4	NaN	0	0	0	0	0	0
Q9DC51	Gnai3	Guanine nucleotide-binding protein G(k) subunit alpha	NaN	0	0	0	0	0	0
Q9DCC4	Pycrl	Pyrroline-5-carboxylate reductase 3	NaN	0	0	0	0	0	0
Q9DCD0	Pgd	6-phosphogluconate dehydrogenase, decarboxylating	NaN	0	0	0	0	0	0
Q9DCH4	Eif3f	Eukaryotic translation initiation factor 3 subunit F	NaN	0	0	0	0	0	0
Q9DCS9;D3YUK4	Ndufb10	ADH dehydrogenase [ubiquinone] 1 beta subcomplex subunit 10	NaN	0	0	0	0	0	0
Q9DCT2	Ndufs3	H dehydrogenase [ubiquinone] iron-sulfur protein 3, mitochondrial	NaN	0	0	0	0	0	0
Q9DCW4;A0A0U1RNR3;A0A	Etfb	Electron transfer flavoprotein subunit beta	NaN	0	0	0	0	0	0
Q9DD18	Dtd1	D-tyrosyl-tRNA(Tyr) deacylase 1	NaN	0	0	0	0	0	0
Q9DD24	Wbp5	WW domain-binding protein 5	NaN	0	0	0	0	0	0
Q9EPE9	Atp13a1	Manganese-transporting ATPase 13A1	NaN	0	0	0	0	0	0
Q9EPL8	Ipo7	Importin-7	NaN	0	0	0	0	0	0
Q9EQH3	Vps35	Vacuolar protein sorting-associated protein 35	NaN	0	0	0	0	0	0
Q9EQP2	Ehd4	EH domain-containing protein 4	NaN	0	0	0	0	0	0
Q9QX15;Q9EQR4;A0A0G2JG	Clca3a1;Clca3a2;Clca3b		NaN	0	0	0	0	0	0
Q9ER00	Stx12	Syntaxin-12	NaN	598680	200340	0	0	0	0
Q9ER05	Ctrl		NaN	0	0	0	0	0	0
Q9ER72-2;Q9ER72;A0A140LI	Cars	Cysteine--tRNA ligase, cytoplasmic	NaN	0	0	0	0	0	0
Q9ER73	Elp4	Elongator complex protein 4	NaN	0	0	0	0	0	0
Q9ER81	Tor1aip2	Torsin-1A-interacting protein 2, isoform IFRG15	NaN	0	0	0	0	0	0
Q9ERD7	Tubb3	Tubulin beta-3 chain	NaN	0	0	0	0	0	0
Q9ERG0-2;Q9ERG0	Lima1	LIM domain and actin-binding protein 1	NaN	0	0	0	0	0	0
Q9ERN0	Scamp2	Secretory carrier-associated membrane protein 2	NaN	0	0	0	0	0	0
Q9ET22	Dpp7	Dipeptidyl peptidase 2	NaN	0	0	0	0	0	0
Q9JHF5;F6ZFB8	Tcirg1	V-type proton ATPase subunit a	NaN	0	0	0	0	0	0
Q9JHP7-3;Q9JHP7-2;Q9JHP7	Kdelc1	KDEL motif-containing protein 1	NaN	0	0	0	0	0	0
Q9JHU9;A0A1B0GS86	Isyna1	Inositol-3-phosphate synthase 1	NaN	0	0	0	0	0	0
Q9JIG7	Ccdc22	Coiled-coil domain-containing protein 22	NaN	0	0	0	0	0	0
Q9JII6;B1AXW3	Akr1a1	Alcohol dehydrogenase [NADP(+)]	NaN	0	0	0	0	0	0
Q9JIK5	Ddx21	Nucleolar RNA helicase 2	NaN	0	0	0	0	0	0
Q9JJ28	Flii	Protein flightless-1 homolog	NaN	0	0	0	0	0	0
Q9JKF1	Iqgap1	Ras GTPase-activating-like protein IQGAP1	NaN	0	0	0	0	0	0
Q9JKR6;F6TRP3;A0A1L1SQ3	Hyou1	Hypoxia up-regulated protein 1	NaN	0	0	167420	1363000	0	0
Q9JKY0	Rqcd1	Cell differentiation protein RCD1 homolog	NaN	0	0	0	0	0	0
Q9JMG1	Edf1	Endothelial differentiation-related factor 1	NaN	0	0	0	0	0	0
Q9JMH6-2;Q9JMH6;A0A1W2	Txnrd1	Thioredoxin reductase 1, cytoplasmic	NaN	0	0	0	0	0	0
Q9QUR6	Prep	Prolyl endopeptidase	NaN	0	0	0	0	0	0
Q9QUR7	Pin1	Peptidyl-prolyl cis-trans isomerase NIMA-interacting 1	NaN	0	0	0	0	0	0
Q9QZE5;Q7TNQ1	Copg1	Coatomer subunit gamma-1	NaN	0	0	0	0	0	0
Q9QZM0	Ubqln2	Ubiquilin-2	NaN	0	0	0	0	0	0
Q9QZS8-2;Q9QZS8	Sh2d3c	SH2 domain-containing protein 3C	NaN	0	0	0	0	0	0
Q9QZZ4-3;Q9QZZ4-2;Q9QZZ	Myo15a;Myo15	Unconventional myosin-XV	NaN	0	0	0	0	0	0

Q9R0E1	Plod3	Procollagen-lysine,2-oxoglutarate 5-dioxygenase 3	NaN	0	0	3206100	714420	0	0
Q9R0H0-2;Q9R0H0	Acox1	Peroxisomal acyl-coenzyme A oxidase 1	NaN	0	0	0	0	0	0
Q9R0X4	Acot9	Acyl-coenzyme A thioesterase 9, mitochondrial	NaN	0	0	0	0	0	0
Q9R190	Mta2	Metastasis-associated protein MTA2	NaN	0	0	0	0	0	0
Q9R1E0	Foxo1	Forkhead box protein O1	NaN	0	0	0	0	0	0
Q9R1J0	Nsdhl	Sterol-4-alpha-carboxylate 3-dehydrogenase, decarboxylating	NaN	0	0	0	0	0	0
Q9R1P3	Psmb2	Proteasome subunit beta type-2	NaN	0	0	0	0	0	0
Q9R1P4	Psma1	Proteasome subunit alpha type-1	NaN	0	0	0	0	0	0
Q9R1Q6	Tmem176b	Transmembrane protein 176B	NaN	0	0	0	0	0	0
Q9R1Q7	Plp2	Proteolipid protein 2	NaN	0	0	0	0	0	0
Q9WTL7	Lypla2	Acyl-protein thioesterase 2	NaN	0	0	0	0	2609000	912800
Q9WTP6-2;Q9WTP6;F7BP55	Ak2	;e 2, mitochondrial;Adenylate kinase 2, mitochondrial, N-terminally processed	NaN	0	0	0	0	0	0
Q9WTR5	Cdh13	Cadherin-13	NaN	0	0	0	0	0	0
Q9WTX5	Skp1	S-phase kinase-associated protein 1	NaN	0	0	0	0	0	0
Q9WU78;Q9WU78-3;Q9WU	Pdcd6ip	Programmed cell death 6-interacting protein	NaN	0	0	12016000	2785300	0	0
Q9WUM4	Coro1c	Coronin-1C	NaN	0	0	0	0	0	0
Q9WUU7	Ctsz	Cathepsin Z	NaN	0	0	0	0	0	0
Q9WV66	07-Mar	E3 ubiquitin-protein ligase MARCH7	NaN	0	0	0	0	0	0
Q9WVA2;Q4FZG7	Timm8a1;Timm8a2	anslocase subunit Tim8 A;Putative mitochondrial import inner membrane translocase	NaN	0	0	0	0	0	0
T1ECW4;Q9WVB0;Q9WVB0-	Rbpms	RNA-binding protein with multiple splicing	NaN	0	0	0	0	0	0
Q9Z0F7	Sncg	Gamma-synuclein	NaN	0	0	0	0	0	0
Q9Z0J7	Gdf15	Growth/differentiation factor 15	NaN	0	0	0	0	0	0
Q9Z0P4;Q9Z0P4-2;A0A1W2F	Palm	Paralemmin-1	NaN	0	0	0	0	0	0
Q9Z110-2;Q9Z110;D3Z0B4	Aldh18a1	-carboxylate synthase;Glutamate 5-kinase;Gamma-glutamyl phosphate reductase	NaN	0	0	0	0	2135900	2102600
Q9Z131-3;Q9Z131-2;Q9Z131	Sh3bp5	SH3 domain-binding protein 5	NaN	0	0	0	0	0	0
Q9Z1A1;F6QJV5;B8JJG9;B8JJ	Tfg		NaN	0	0	0	0	0	0
Q9Z1G3	Atp6v1c1	V-type proton ATPase subunit C 1	NaN	0	0	0	0	0	0
Q9Z1M8;A0A494BAW7	Ik	Protein Red	NaN	0	0	0	0	0	0
Q9Z1N5	Ddx39b	Spliceosome RNA helicase Ddx39b	NaN	0	0	0	0	0	0
Q9Z1Q5	Clic1	Chloride intracellular channel protein 1	NaN	0	0	0	0	0	0
Q9Z1Z2	Strap	Serine-threonine kinase receptor-associated protein	NaN	0	0	0	0	0	0
V9GXI0;V9GXT7;Q9Z266	Snapin	SNARE-associated protein Snapin	NaN	0	0	0	0	0	0
Q9Z277-2;Q9Z277	Baz1b	Tyrosine-protein kinase BAZ1B	NaN	0	0	0	0	0	0
Q9Z2A7	Dgat1	Diacylglycerol O-acyltransferase 1	NaN	0	0	0	0	0	0
Q9Z2M7	Pmm2	Phosphomannomutase 2	NaN	0	0	0	0	0	0
Q9Z2N8;A0A0A6YWR1;A0A0	Actl6a	Actin-like protein 6A	NaN	0	0	0	0	0	0
Q9Z2Z6	Slc25a20	Mitochondrial carnitine/acylcarnitine carrier protein	NaN	0	0	0	0	0	0
Q9Z315	Sart1	U4/U6.U5 tri-snRNP-associated protein 1	NaN	0	0	0	0	0	0
