# Supplementary material for: The role of aging on endothelial cell–cell junctions and pulmonary microvascular permeability in male mice
Source: Physiol Rep. 2025 Dec 19;13(24):e70686. doi: 10.14814/phy2.70686 (PMC12717451; doi:10.14814/phy2.70686)
Supplement: Supplementary file 4 — Table S2. [file PHY2-13-e70686-s001.pdf]

Supplementary Table 2 | Differentially enriched proteins from young mice associated with actin cytoskeleton.

Gene name	Protein name
Stmn1	stathmin 1
Dync1h1	dynein cytoplasmic 1 heavy chain 1
Plec	plectin
Dbnl	drebrin-like
Hsp90ab1	heat shock protein 90 alpha (cytosolic), class B member 1
Lmnb1	lamin B1
Tln1	talin 1
Arpc3	actin related protein 2/3 complex, subunit 3
Tmsb4x	thymosin, beta 4, X chromosome
Pacsin2	protein kinase C and casein kinase substrate in neurons 2
Ezr	ezrin
Capza2	capping actin protein of muscle Z-line subunit alpha 2
Csrp1	cysteine and glycine-rich protein 1
Actb	actin, beta
Actr2	actin related protein 2
Capzb	capping actin protein of muscle Z-line subunit beta
Sptbn1	spectrin beta, non-erythrocytic 1
Hspg2	perlecan (heparan sulfate proteoglycan 2)
Gsn	gelsolin
Marcksl1	MARCKS-like 1
Sntb2	syntrophin, basic 2
Vim	vimentin
Tmod3	tropomodulin 3
