# Supplementary material for: The role of aging on endothelial cell–cell junctions and pulmonary microvascular permeability in male mice
Source: Physiol Rep. 2025 Dec 19;13(24):e70686. doi: 10.14814/phy2.70686 (PMC12717451; doi:10.14814/phy2.70686)
Supplement: Supplementary file 5 — Table S3. [file PHY2-13-e70686-s003.pdf]

Supplementary Table 3 | Differentially enriched proteins from aged mice associated with actin cytoskeleton.

Gene name	Protein name
Itga4	integrin alpha 4
Add1	adducin 1
Anxa2	annexin A2
Lmna	lamin A
Cnn2	calponin 2
Tpx2	TPX2, microtubule-associated
Dpysl3	dihydropyrimidinase-like 3
Myo1c	myosin IC
Flnc	filamin C, gamma
Cav1	caveolin 1, caveolae protein
Krt10	keratin 10
Tpm3	tropomyosin 3, gamma
Tagln2	transgelin 2
Fn1	fibronectin 1
Tpm4	tropomyosin 4
Cald1	caldesmon 1
Actn4	actinin alpha 4
Pdlim7	PDZ and LIM domain 7
Pdlim1	PDZ and LIM domain 1 (elfin)
Fus	fused in sarcoma
Gmfb	glia maturation factor, beta
Pfn1	profilin 1
Dstn	destrin
Ran	RAN, member RAS oncogene family
Ehd2	EH-domain containing 2
Cfl1	cofilin 1, non-muscle
Flnb	filamin, beta
Syne1	spectrin repeat containing, nuclear envelope 1
